# Supplementary material for: On the Origin and Propagation of the COVID-19 Outbreak in the Italian Province of Trento, a Tourist Region of Northern Italy
Source: Viruses. 2022 Mar 11;14(3):580. doi: 10.3390/v14030580 (PMC8951735; doi:10.3390/v14030580)
Supplement: Supplementary file 1 [file viruses-14-00580-s001.zip › Supplementary Table S5-merged_acknowledgement.pdf]

We gratefully acknowledge the following Authors from the Originating laboratories responsible for obtaining the specimens, as well as the Submitting laboratories where the genome data were generated and shared via GISAID, on which this research is based.

All Submitters of data may be contacted directly via www.gisaid.org

Authors are sorted alphabetically.

Accession ID	Originating Laboratory	Submitting Laboratory	Authors
EPI_ISL_455453	1. ViroGenetics - BSL3 Laboratory of Virology, Małopolska Centre of Biotechnology, Jagiellonian University; 2. II Department of Internal Medicine, Faculty of Medicine, Jagiellonian University Medical College; 3. Narodowy Instytut Zdrowia Publicznego – Państwowy Zakład Higieny (NIZP-PZH)	1. ViroGenetics - BSL3 Laboratory of Virology, Małopolska Centre of Biotechnology, Jagiellonian University; 2. II Department of Internal Medicine, Faculty of Medicine, Jagiellonian University Medical College; 3. Narodowy Instytut Zdrowia Publicznego – Państwowy Zakład Higieny (NIZP-PZH).	Agnieszka Kołakowska-Kulesza; Aleksandra A. Zasada; Aleksandra Milewska; Ewelina Hallman-Szelińska; Katarzyna Owczarek; Katarzyna Pancer; Katarzyna Zacharczuk; Krzysztof Pyrć; Magdalena Rzeczkowska; Marek Sanak; Natalia Wolaniuk; Paweł P Łabaj; Tomasz Wołkowicz; Wojciech Branicki
EPI_ISL_542187, EPI_ISL_542225, EPI_ISL_542233, EPI_ISL_542412, EPI_ISL_542418	ASST GOM Niguarda	Dep. Of Oncology and Hemato-Oncology University of Milan	Antonio Piralla; Carlo Federico Perno; Chiara Vismara; Claudia Alteri; Elisa Matarazzo; Fausto Baldanti; Federica Giardina; Federica Novazzi; Luna Colagrossi; Maria Antonello; Massimo Puoti; Monica Tallarita; Oscar Massimiliano Epis; Roberto Fumagalli; Silvia Renica; Stefano Gaiarsa; Valentino Costabile; Valeria Cento
EPI_ISL_569866, EPI_ISL_569867, EPI_ISL_569868, EPI_ISL_569872, EPI_ISL_569875, EPI_ISL_569880, EPI_ISL_569886			
see above	Amedeo di savoia	Crosetto lab, Karolinska Institutet, SciLifeLab	Anna Sapino; Luuk Harbers; Maria Grazia Milia; Michele Simonetti; Nicola Crosetto; Ning Zhang; Valeria Ghisetti
EPI_ISL_500614, EPI_ISL_527015	Area of Virology, Serology and Virology Division (SAViD), New South Wales Health Pathology Randwick	Area of Virology, Serology and Virology Division (SAViD), New South Wales Health Pathology Randwick	Rawlinson, W.
EPI_ISL_457825	Army Medical Research Center - Scientific Department	Army Medical and Veterinary Research Center	Anna Anselmo; Antonella Fortunato; Florigio Lista; Francesco Giordani; Giovanni Faggioni; Nino D'Amore; Riccardo De Sanctis; Silvia Fillo; Vanessa Vera Fain
EPI_ISL_475830, EPI_ISL_475832, EPI_ISL_475837, EPI_ISL_475845, EPI_ISL_475854, EPI_ISL_475860, EPI_ISL_475862, EPI_ISL_475863, EPI_ISL_475869, EPI_ISL_475875, EPI_ISL_475879, EPI_ISL_475880, EPI_ISL_475886			
see above	Austrian Agency for Health and Food Safety (AGES)	Bergthaler laboratory, CeMM Research Center for Molecular Medicine of the Austrian Academy of Sciences	Alexander Lercher; Alexandra Popa; Andreas Bergthaler; Benedikt Agerer; Christoph Bock; Daniela Schmid; Dorothee von Laer; Elisabeth Puchhammer-Stoeckl; Franz Allerberger; Gregor Hörmann; Guenter Weiss; Henrique Colaco; Jakob-Wendelin Genger; Jan Laine; Judith Aberle; Kinga Rigler-Hohenwarter; Lukas Endler; Manfred Nairz; Mark Smyth; Martin Senekowitsch; Michael Schuster; Peter Hufnagl; Rainer Gattringer; Stephan Aberle; Thomas Penz; Wegene Borena
EPI_ISL_516079, EPI_ISL_516081, EPI_ISL_516082, EPI_ISL_516083, EPI_ISL_516084, EPI_ISL_516085, EPI_ISL_516086, EPI_ISL_516087, EPI_ISL_516088			
see above	Biomedical Sciences and Public Health, Polytechnic University of Marche	Biomedical Sciences and Public Health, Polytechnic University of Marche	Alessandrini, F.; Bagnarelli, P.; Caucci, S.; Di Sante, L.; Melchionda, F.; Menzo, S.; Onofri, V.; Tagliabracci, A.; Turchi, C.
EPI_ISL_421455, EPI_ISL_421485, EPI_ISL_421486	CH Barreiro Montijo	Instituto Nacional de Saude (INSA)	Guiomar et al
EPI_ISL_453813	CH Leiria	Instituto Nacional de Saude (INSA)	Borges et al
EPI_ISL_454008	CH Porto - H Sto Antonio	Instituto Nacional de Saude (INSA)	Borges et al
EPI_ISL_421453, EPI_ISL_453821, EPI_ISL_453822, EPI_ISL_453826	CHTMAD	Instituto Nacional de Saude (INSA)	Borges et al; Guiomar et al
EPI_ISL_418219	CHU - Hôpital Cavale Blanche - Labo. de Virologie	National Reference Center for Viruses of Respiratory Infections, Institut Pasteur, Paris	Angela Brisebarre; Etienne Simon-Lorière; Fabiana Gambaro; Flora Donati; Léa Pilorge; Marion Barbet; Maud Vanpeene; Mélanie Albert; Méline Bizard; Sylvie Behillil; Sylvie van der Werf; Vincent Enouf
EPI_ISL_525537, EPI_ISL_525542	CNR Virus des Infections Respiratoires - France SUD	CNR Virus des Infections Respiratoires - France SUD	Alexandre Gaymard; Antonin Bal; Bruno Lina; Florence Morfin-Sherpa; Gregory Destras; Gwendolyne Burfin; Laurence Josset; Martine Valette; Maude Bouscambert-Duchamp; Solenne Brun
EPI_ISL_451935	CUB Hopital Erasme Laboratoire d'Anatomie Pathologique	CUB Hopital Erasme Laboratoire d'Anatomie Pathologique	Dr. Nicky D'Haene; Prof. Isabelle Salmon
EPI_ISL_438030, EPI_ISL_438046, EPI_ISL_438068, EPI_ISL_438080, EPI_ISL_438121, EPI_ISL_475811	Center for Virology, Medical University of Vienna	Bergthaler laboratory, CeMM Research Center for Molecular Medicine of the Austrian Academy of Sciences	Alexander Lercher; Alexandra Popa; Andreas Bergthaler; Benedikt Agerer; Christoph Bock; Daniela Schmid; Dorothee von Laer; Elisabeth Puchhammer-Stoeckl; Franz Allerberger; Gregor Hörmann; Guenter Weiss; Henrique Colaco; Jakob-Wendelin Genger; Jan Laine; Judith Aberle; Kinga Rigler-Hohenwarter; Lukas Endler; Manfred Nairz; Mark Smyth; Martin Senekowitsch; Michael Schuster; Peter Hufnagl; Rainer Gattringer; Stephan Aberle; Thomas Penz; Wegene Borena
EPI_ISL_417464, EPI_ISL_425125	Center of Medical Microbiology, Virology, and Hospital Hygiene, University of Duesseldorf	Center of Medical Microbiology, Virology, and Hospital Hygiene, University of Duesseldorf	Alexander Dilthey; Andreas Walker; Björn-Erik Jensen; Daniel Strelow; Detlef Kindgen-Milles; Jörg Timm; Klaus Pfeffer; Malte Kohns Vasconcelos; Marcel Andree; Ortwin Adams; Sandra Hauka; Tina Senff; Tobias Wienemann; Torsten Feldt; Torsten Houwaart
EPI_ISL_486417	Centrala laboratorija	Latvian Biomedical Research and Study Centre	Ivars Silamiķelis; Jana Osīte; Jānis Kloviņš; Kaspars Megnis; Marta Priedīte; Monta Ustinova; Stella Lapiņa; Uga Dumpis; Vita Rovīte; Ņikita Zrelovs
EPI_ISL_418218, EPI_ISL_418220, EPI_ISL_418223, EPI_ISL_429968	Centre Hospitalier Compiègne Laboratoire de Biologie	National Reference Center for Viruses of Respiratory Infections, Institut Pasteur, Paris	Angela Brisebarre; Etienne Simon-Lorière; Fabiana Gambaro; Flora Donati; Marion Barbet; Maud Vanpeene; Mélanie Albert; Méline Bizard; Raulin Olivia; Sylvie Behillil; Sylvie van der Werf; Vincent Enouf
EPI_ISL_450523	Centrālā Laboratorija	Latvian Biomedical Research and Study Centre	Ivars Silamiķelis; Jana Osīte; Jānis Kloviņš; Kaspars Megnis; Marta Priedīte; Monta Ustinova; Stella Lapiņa; Uga Dumpis; Vita Rovīte; Ņikita Zrelovs
EPI_ISL_486411, EPI_ISL_486418, EPI_ISL_486421, EPI_ISL_486437, EPI_ISL_501896	Centrālā laboratorija	Latvian Biomedical Research and Study Centre	Ivars Silamiķelis; Jana Osīte; Jānis Kloviņš; Kaspars Megnis; Marta Priedīte; Monta Ustinova; Stella Lapiņa; Uga Dumpis; Vita Rovīte; Ņikita Zrelovs
EPI_ISL_516641	Charité Universitätsmedizin Berlin, Institut für Virologie/Labor Berlin	Charité Universitätsmedizin Berlin, Institut für Virologie/Labor Berlin	Barbara Mühlemann; Christian Drosten; Julia Schneider; Jörn Beheim-Schwarzbach; Talitha Veith; Terry Jones; Victor M Corman
EPI_ISL_462451, EPI_ISL_462452, EPI_ISL_462453, EPI_ISL_462455, EPI_ISL_462456, EPI_ISL_462457, EPI_ISL_462459, EPI_ISL_462460, EPI_ISL_462462, EPI_ISL_462464, EPI_ISL_462465, EPI_ISL_462469			
see above	Clinical Center, University of Sarajevo	Charite Universitatsmedizin Berlin, Institute of Virology	Almedina Hadzihasanovic-Moro; Amela Dedeic-Ljubovic; Barbara Muehlemann; Christian Drosten; Irma Salimovic-Besic; Jorn Beheim-Schwarzbach; Julia Schneider; Selma Mutevelic; Suzana Arapcic; Talitha Veith; Terry Jones; Victor M Corman
EPI_ISL_528190	Clinical Virology	Clinical Bacteriology	Adrian Egli; Alexander Gensch; Alfredo Mari; Christian Nickel; Hans Hirsch; Hans Pargger; Helena MB Seth-Smith; Julia Bielicki; Karoline Leuzinger; Kirstine K. Soegaard; Madlen Stange; Manuel Battegay; Martin Siegemund; Michael Osthoff; Michael Schweitzer; Myrta Brunner; Rita Schneider-SliFemalea; Roland Bingisser; Sarah Tschudin-Sutter; Simon Fuchs; Stefano Bassetti; Tim Roloff
EPI_ISL_516922, EPI_ISL_516923, EPI_ISL_516924	Department for Molecular Diagnostics, Centre for Medical Microbiology, Institute of Public Health of Montenegro	Charité Universitätsmedizin Berlin, Institut für Virologie	Barbara Muehlemann; Christian Drosten; Julia Schneider; Jörn Beheim-Schwarzbach; Marija Govedarica and Danijela Vujošević; Talitha Veith; Terry Jones; Victor M Corman
EPI_ISL_417004, EPI_ISL_418655, EPI_ISL_421191, EPI_ISL_421192, EPI_ISL_424630, EPI_ISL_424631, EPI_ISL_424649, EPI_ISL_424654, EPI_ISL_447123, EPI_ISL_447128, EPI_ISL_447130			
see above	Department of Clinical Microbiology	GIGA Medical Genomics	Artesi Maria; Bontems Sébastien; Boreux Raphaël; Bours Vincent.; Cécile Meex; Durkin Keith; Hayette Marie-Pierre; Keith Durkin; Maria Artesi; Marie-Pierre Hayette; Meex Cécile; Melin Pierrette; Pierrette Melin; Raphaël Boreux; Sébastien Bontems; Vincent Bours.
EPI_ISL_451991, EPI_ISL_451992, EPI_ISL_451993, EPI_ISL_451995, EPI_ISL_451997, EPI_ISL_451998, EPI_ISL_452085, EPI_ISL_452100			
see above	Department of Clinical Microbiology, Copenhagen University Hospital, Hvidovre, Kettegaard Alle 30, 2650 Hvidovre.	Albertsen lab, Department of Chemistry and Bioscience, Aalborg University, Denmark	Rasmus Kirkegaard
EPI_ISL_457699, EPI_ISL_457700, EPI_ISL_457721, EPI_ISL_457724, EPI_ISL_457728, EPI_ISL_457732, EPI_ISL_457736			
see above	Department of Infectious Diseases, Istituto Superiore di Sanità, Roma , Italy	Army Medical and Veterinary Research Center	Alessandra Lo Presti; Anna Anselmo; Antonella Fortunato; Antonella Marchi; Concetta Fabiani Silvia Fillo; Concetta Fabiani Silvia Fillo; Eleonora Benedetti; Florigio Lista; Francesco Giordani; Giovanni Faggioni; Nino D'Amore; Paola Stefanelli; Riccardo De Sanctis; Stefano Fiore; Vanessa Vera Fain
EPI_ISL_412973	Department of Infectious Diseases, Istituto Superiore di Sanità, Roma , Italy	Virology Laboratory, Scientific Department, Army Medical Center	Andrea Ciammaruconi; Anna Anselmo; Antonella Fortunato; Antonella Marchi; Concetta Fabiani; Eleonora Benedetti; Florigio Lista; Giovanni Faggioni; Paola Stefanelli; Riccardo De Santis; Silvia Fillo; Stefano Fiore; Stefano Palomba
EPI_ISL_463741, EPI_ISL_463742, EPI_ISL_463745, EPI_ISL_463746, EPI_ISL_463748	Department of Molecular Virology, Cyprus Institute of Neurology and Genetics	Department of Molecular Virology, Cyprus Institute of Neurology and Genetics	Christina Christodoulou; Christina Tryfonos; Dana Koptides; George Krashias; Jan Richter; Stavros Bashiardes
EPI_ISL_425318, EPI_ISL_433667, EPI_ISL_433729	Department of Pathology, University of Cambridge	COVID-19 Genomics UK (COG-UK) Consortium	Aminu S. Jahun; Anna Yakovleva; Charlotte J. Houldcroft; Fahad A Khokhar; Grant Hall; Ian Goodfellow; Laura G Caller; Luke W Meredith; M. Estee Torok; Martin D. Curran; Myra Hosmillo; Sarah L. Caddy; Theresa Feltwell; William L. Hamilton
EPI_ISL_438456, EPI_ISL_438462, EPI_ISL_438471, EPI_ISL_438474, EPI_ISL_438475, EPI_ISL_438500, EPI_ISL_438545, EPI_ISL_439436, EPI_ISL_439937, EPI_ISL_440166, EPI_ISL_440541, EPI_ISL_440605, EPI_ISL_441227, EPI_ISL_441547, EPI_ISL_441587, EPI_ISL_441588, EPI_ISL_441612, EPI_ISL_441617, EPI_ISL_441632, EPI_ISL_441642, EPI_ISL_441660, EPI_ISL_441723, EPI_ISL_441734, EPI_ISL_441768,			

EPI_ISL_441830, EPI_ISL_442225, EPI_ISL_442272, EPI_ISL_443053, EPI_ISL_443447, EPI_ISL_443514, EPI_ISL_443574, EPI_ISL_459178, EPI_ISL_470403, EPI_ISL_489430, EPI_ISL_489435, EPI_ISL_489438, EPI_ISL_489457, EPI_ISL_489467, EPI_ISL_489469, EPI_ISL_489473, EPI_ISL_489497, EPI_ISL_489514, EPI_ISL_489545, EPI_ISL_489546, EPI_ISL_489552, EPI_ISL_489562, EPI_ISL_489564, EPI_ISL_524570, EPI_ISL_524573			
see above	Department of Pathology, University of Cambridge	Wellcome Sanger Institute for the COVID-19 Genomics UK (COG-UK) consortium	Alex Alderton; Aminu S. Jahun; Anna Yakovleva; Charlotte J. Houldcroft; Cordelia Langford; David K. Jackson; Dominic Kwiatkowski; Ewan Harrison; Fahad A Khokhar; Grant Hall; Ian Goodfellow; Ian Johnston; John Sillitoe on behalf of the Wellcome Sanger Institute COVID-19 Surveillance Team (http://www.sanger.ac.uk/covid-team); Laura G Caller; Luke W Meredith; M. Estée Török; Martin D. Curran; Myra Hosmillo; Roberto Amato; Sarah L. Caddy; Sonia Goncalves; Theresa Feltwell; William L. Hamilton; and Alex Alderton
EPI_ISL_418391, EPI_ISL_481559, EPI_ISL_481579, EPI_ISL_481623, EPI_ISL_481644, EPI_ISL_481647, EPI_ISL_481658, EPI_ISL_481681, EPI_ISL_481711, EPI_ISL_481722, EPI_ISL_481732			
see above	Department of Virology and Immunology, University of Helsinki and Helsinki University Hospital, Huslab Finland	Department of Virology, Faculty of Medicine, University of Helsinki, Helsinki, Finland	Hannimari Kallio-Kokko; Harri Kangas; Jenni Virtanen; Maija Suvanto; Olli Vapalahti; Pekka Ellonen; Sari Hannula; Teemu Smura
EPI_ISL_429341, EPI_ISL_429346, EPI_ISL_429353, EPI_ISL_429408, EPI_ISL_429414, EPI_ISL_429425, EPI_ISL_429447, EPI_ISL_429484, EPI_ISL_429507, EPI_ISL_429549, EPI_ISL_437658, EPI_ISL_437660, EPI_ISL_437661, EPI_ISL_437672, EPI_ISL_437678, EPI_ISL_444854			
see above	Department of Virus and Microbiological Special Diagnostics, Statens Serum Institut, Copenhagen, Denmark, Artillerivej 5, 2300 Copenahgen S	Albertsen lab, Department of Chemistry and Bioscience, Aalborg University, Denmark	Rasmus Kirkegaard
EPI_ISL_414441, EPI_ISL_414448, EPI_ISL_414456, EPI_ISL_414464, EPI_ISL_422642, EPI_ISL_422758, EPI_ISL_455151, EPI_ISL_455242, EPI_ISL_461069, EPI_ISL_461207, EPI_ISL_461247, EPI_ISL_461255, EPI_ISL_461258, EPI_ISL_461259, EPI_ISL_461260, EPI_ISL_461273, EPI_ISL_461289, EPI_ISL_461291, EPI_ISL_461292, EPI_ISL_461316, EPI_ISL_461321, EPI_ISL_461323, EPI_ISL_461360, EPI_ISL_523410, EPI_ISL_523439, EPI_ISL_523443, EPI_ISL_523603			
see above	Dutch COVID-19 response team	Erasmus Medical Center	Anne van der Linden; Annemiek van der Eijk; Aura Timen; Bas Oude Munnink; Claudia Schapendonk; Corien Swaan; Corine GeurtsvanKessel; David Nieuwenhuijse; Irina Chestakova; Jeroen van Kampen; Jolanda Voermans; Madelief Mollers; Manon Haverkate; Marion Koopmans; Mark Pronk; Mart Stein; Pascal Lexmond; Reina Sikkema; Richard Molenkamp; Sandra Kengne Kamga Mobou; Stefan van Nieuwkoop; Theo Bestebroer; on behalf of the Dutch national COVID-19 response team.
EPI_ISL_426289, EPI_ISL_492993	E. Gulbja Laboratorija	Latvian Biomedical Research and Study Centre	Dmitrijs Perminovs; Ivars Silamiķelis; Jānis Kloviņš; Kaspars Megnis; Mikus Gavars; Monta Ustinova; Uga Dumpis; Vita Rovīte; Ņikita Zrelovs
EPI_ISL_526941	Faroese National Reference Laboratory for Fish and Animal Diseases	Faroese National Reference Laboratory for Fish and Animal Diseases	Debes Hammershaimb Christiansen; Maria Marjunardóttir Dahl; Petra Elisabeth Petersen
EPI_ISL_451963, EPI_ISL_451969	Federal Budget Institution of Science, State Research Center for Applied Microbiology & Biotechnology	Federal Budget Institution of Science, State Research Center for Applied Microbiology & Biotechnology	Abaimova A; Bakhteeva I; Blagodatskikh S; Bogun A; Borzilov A; Chekan L; Chernysh S; Denisenko E; Dentovskaya S; Detushev K; Detusheva E; Dyatlov I; Firstova V; Frolov V; Fursov M; Fursova N; Galkina E; Gapelchenkova T; Goncharova J; Gorbatov A; Hlyntseva A; Ivanov S; Kalmantayev T; Kalmantayeva O; Kanashenko M; Kartsev N; Kartseva A; Khomyakov A; Khramov M; Kislichkina A; Kolchanova A; Koroleva-Ushakova A; Kosilova I; Krasilnikova E; Kuzin V; Kuzina E; Makarova M; Marin M; Novikova T; Platonov M; Podkopaev Y; Ryabko A; Shaikhutdinova R; Shemyakin I; Shishkina L; Silkina M; Sizova A; Skryabin Y; Slukin P; Slukina N; Solomentsev V; Solovieva A; Teymurazov M; Timofeev V; Titareva G; Trunyakova A; Tyurin E; Vagayskaya A; Zeninskaya N; Zhumakaev R
EPI_ISL_549168	Furst Medical Laboratory	Norwegian Institute of Public Health, Department of Virology	Hilde Elshaug; Hilde Synnøve Vollan; Kamilla Heddeland Instefjord; Karoline Bragstad; Kathrine Stene-Johansen; Olav Hungnes; Rasmus Riis Kopperud
EPI_ISL_421481, EPI_ISL_453868, EPI_ISL_453939, EPI_ISL_453958	H Beatriz Angelo	Instituto Nacional de Saude (INSA)	Borges et al; Guiomar et al
EPI_ISL_421447, EPI_ISL_421448	H Guimaraes	Instituto Nacional de Saude (INSA)	Guiomar et al
EPI_ISL_421462, EPI_ISL_421490, EPI_ISL_453832, EPI_ISL_453839, EPI_ISL_453915	H Santarem	Instituto Nacional de Saude (INSA)	Borges et al; Guiomar et al
EPI_ISL_445268, EPI_ISL_445280	HOSPITAL REG.LAUTARO NAVARRO AVARIA	Instituto de Salud Publica de Chile	Alejandra Acevedo; Andrés E Castillo; Bárbara Parra; Carolina Tambley; Gabriel Leal; Jaime Lagos; Jorge Fernandez; Loredana Arata; Patricia Bustos; Paz Tapia; Rodrigo Fasce; Winston Andrade
EPI_ISL_421463	HSE Ilha Terceira - Angra do Heroismo	Instituto Nacional de Saude (INSA)	Guiomar et al
EPI_ISL_475094, EPI_ISL_475116	Halmstad klinisk mikrobiologi	The Public Health Agency of Sweden	Anna Risberg; Anna-Malin Linde; Karin Tegmark-Wisell; Maria Lind Karlberg; Mattias Haukland; Olov Svartstrom; Oskar Karlsson Lindsjo; Petra Edquist; Reza Advani; Sandra Broddesson; Shamam Muradrasoli
EPI_ISL_450498	Health Board Laboratory of Communicable Diseases	Charite Universitatsmedizin Berlin, Institute of Virology	Barbara Mühlemann; Christian Drosten; Julia Schneider; Jörn Beheim-Schwarzbach; Liidia Dotsenko; Natalja Kuznetsova; Talitha Veith; Terry Jones; Victor M Corman
EPI_ISL_420065, EPI_ISL_420067	Health Board Laboratory of Communicable Diseases	Charité Universitätsmedizin Berlin, Institute of Virology	Barbara Mühlemann; Christian Drosten; Jörn Beheim-Schwarzbach; Julia Schneider; Liidia Dotsenko; Natalja Kuznetsova; Talitha Veith; Terry Jones; Victor M Corman
EPI_ISL_430469	Hellenic Pasteur Institute, Public Health Laboratories	Hellenic Pasteur Institute, Public Health Laboratories, Unit of Bioinformatics and Applied Genomics	Andreas Mentis; Androniki Voulgari-Kokota; Antonios Kalliaropoulos; Aspasia Kontou; Athanasios Kossyvakis; Evangelidou Maria; Horefti Elina; Timokratis Karamitros; Vasiliki Pogka
EPI_ISL_451652, EPI_ISL_451653	Hematology Laboratory, Section of Molecular Diagnostics, University Clinical Centre, Medical University of Gdansk	Laboratory of Recombinant Vaccines	Adam Sodol; Aneta Szulc; Boguslaw Szewczyk; Ewa Milosz; Krystyna Bienkowska-Szewczyk; Krzysztof Lewandowski; Lukasz Rabalski; Marlena Robakowska
EPI_ISL_539496	Hospital Nostra Senyora de Meritxell	Instituto de Salud Carlos III	A. Monzón; F. Casas; F. Fernández; I; I. Jiménez; Iglesias-Caballero; M. Camarero; M. Cuesta; M. González-Esguevillas; M. Molinero Calamita; M. Zaballos; P. Jiménez; S. Juliá; S. Pozo; S. Varona
EPI_ISL_530079	Hospital Universitario La Paz	Hospital Universitario La Paz	Elias Dahdouh; Esther Viedma; Fernando Lázaro; Jesús Mingorance; Juan Carlos Galán; Julio García; María Rodríguez; Mª Dolores Folgueira; Natalia Stella; Rafael Cantón; Rafael Delgado; Raúl Recio; Sara González
EPI_ISL_530118	Hospital Universitario Ramón y Cajal	Hospital Universitario La Paz	Elias Dahdouh; Esther Viedma; Fernando Lázaro; Jesús Mingorance; Juan Carlos Galán; Julio García; María Rodríguez; Mª Dolores Folgueira; Natalia Stella; Rafael Cantón; Rafael Delgado; Raúl Recio; Sara González
EPI_ISL_526215, EPI_ISL_526230, EPI_ISL_526232	Hungarian Defence Forces Military Medical Centre	National Laboratory of Virology, Szentágothai Research Centre	Balázs Somogyi; Bálint Eszenyi; Endre Gábor Tóth; Ferenc Jakab; Gábor Kemenesi
EPI_ISL_419255	INMI Lazzaro Spallanzani IRCCS	INMI Lazzaro Spallanzani IRCCS	Antonino Di Caro; Barbara Bartolini; Cesare E. M. Gruber; Concetta Castilletti; Daniele Lapa; Eleonora Lalle; Emanuela Giombini; Fabrizio Carletti; Francesca Colavita; Francesco Messina; Giuseppe Ippolito.; Maria R. Capobianchi; Martina Rueca
EPI_ISL_417921, EPI_ISL_417922, EPI_ISL_417923, EPI_ISL_419254, EPI_ISL_424342, EPI_ISL_424344	INMI Lazzaro Spallanzani IRCCS	Laboratory of Virology, INMI Lazzaro Spallanzani IRCCS	Antonino Di Caro; Barbara Bartolini; Cesare E. M. Gruber; Cesare Ernesto Maria Gruber; Concetta Castilletti; Daniele Lapa; Eleonora Lalle; Emanuela Giombini; Fabrizio Carletti; Francesca Colavita; Francesco Messina; Francesco Vairo; Giulia Matusali; Giuseppe Ippolito; Giuseppe Ippolito.; Licia Bordi; Maria R. Capobianchi; Maria Rosaria Capobianchi; Martina Rueca
EPI_ISL_453924	INMLCF - Sul	Instituto Nacional de Saude (INSA)	Borges et al
EPI_ISL_421452, EPI_ISL_421456, EPI_ISL_453817, EPI_ISL_453834, EPI_ISL_453916	INSA	Instituto Nacional de Saude (INSA)	Borges et al; Guiomar et al
EPI_ISL_492184, EPI_ISL_494771	INT Fondazione Pascale	INT Fondazione Pascale	INT Fondazione Pascale; Pascale
EPI_ISL_475828	Institut für Virologie am Department für Hygiene, Mikrobiologie und Public Health	Bergthaler laboratory, CeMM Research Center for Molecular Medicine of the Austrian Academy of Sciences	Alexander Lercher; Alexandra Popa; Andreas Bergthaler; Benedikt Agerer; Christoph Bock; Daniela Schmid; Dorothee von Laer; Elisabeth Puchhammer-Stoeckl; Franz Allerberger; Gregor Hörmann; Guenter Weiss; Henrique Colaco; Jakob-Wendelin Genger; Jan Laine; Judith Aberle; Kinga Rigler-Hohenwarter; Lukas Endler; Manfred Nairz; Mark Smyth; Martin Senekowitsch; Michael Schuster; Peter Hufnagl; Rainer Gattringer; Stephan Aberle; Thomas Penz; Wegene Borena
EPI_ISL_574792, EPI_ISL_574800, EPI_ISL_574801, EPI_ISL_574802	Institute for Infectious Diseases, University of Bern	Institute for Infectious Diseases, University of Bern	Alban Ramette; Christian Baumann; Cora Sägesser; Franziska Suter-Riniker; Michel C Koch; Miguel A Terrazos Miani; Peter Keller; Stephen L Leib
EPI_ISL_454606, EPI_ISL_455566, EPI_ISL_468656	Institute for Public Health	Laboratory for advanced genomics	Filip Rokić; Igor Jurak; Lovro Trgovec-Greif; Neven Sučić; Oliver Vugrek; Tomislav Rukavina
EPI_ISL_572330	Institute for Virology, University Hospital Duesseldorf, Medical Faculty, Heinrich-Heine-University Duesseldorf	Institute for Virology, University Hospital Duesseldorf, Medical Faculty, Heinrich-Heine-University Duesseldorf	; Alexander Killer; Andreas Walker; Annemarie Mohring; Anselm Kunstein; Ansgar Schulz; Björn Jensen; Caroline Klindt; Edwin Bölke; Gerald Antoch; Heiner Schaal; Jennifer Neubert; Johannes Bode; Johannes C. Fischer; Jörg Timm; Lisa Müller; Maximilian Damagnez; Nadine Lübke; Ortwin Adams; Philipp Albrecht; Philipp Ostermann; Saskia Elben; Tina Senff; Tom Lüdde; Torsten Feldt; Verena Keitel
EPI_ISL_420294, EPI_ISL_420295	Institute of Microbiology and Immunology, Faculty of Medicine, University of Ljubljana	Institute of Microbiology and Immunology, Faculty of Medicine, University of Ljubljana	Lucijan Skubic; Mario Poljak; Miša Korva; Samo Zakotnik; Tatjana Avšič - Županc; Tomaž Mark Zorec
EPI_ISL_511314	Instituto Nacional de Saude (INSA) and Instituto Gulbenkian de Ciencia (IGC)	Instituto Nacional de Saude (INSA) and Instituto Gulbenkian de Ciencia (IGC)	Borges et al
EPI_ISL_477194, EPI_ISL_477202, EPI_ISL_477203	Istituto Zooprofilattico Sperimentale Puglia e Basilicata;	Beaconlab (Bioinformatics, Evolution and Comparative Genomics lab), Dept of Biosciences, University on Mila	Chiara M.; Manzari C.; Parisi A.; Pesole G.
EPI_ISL_477198, EPI_ISL_477200	Istituto Zooprofilattico Sperimentale Puglia e Basilicata;	Beaconlab (Bioinformatics, Evolution and Comparative Genomics lab), Dept of Biosciences, University on Milan	Chiara M.; Manzari C.; Parisi A.; Pesole G.
EPI_ISL_451962	Istituto Zooprofilattico Sperimentale Puglia e Basilicata; Dipartimento di Bioscienze, Biotecnologie e Biofarmaceutica dell'Università degli Studi di Bari "A.Moro"; Istituto di Biomembrane. Bioenergetica e Biotecnologie Molecolari del Consiglio Nazionale delle Ricerche di Bari	Beaconlab (Bioinformatics, Evolution and Comparative Genomics lab), Dept of Biosciences, University on Milan	Chiara M.; Manzari C.; Parisi A.; Pesole G.

EPI_ISL_469016, EPI_ISL_469018, EPI_ISL_469019, EPI_ISL_469021, EPI_ISL_469023, EPI_ISL_525555, EPI_ISL_525556, EPI_ISL_525557, EPI_ISL_525562, EPI_ISL_525563, EPI_ISL_525564, EPI_ISL_525565, EPI_ISL_525566, EPI_ISL_525567, EPI_ISL_525568, EPI_ISL_525571			
see above	Istituto Zooprofilattico Sperimentale Puglia e Basilicata; Dipartimento di Bioscienze, Biotecnologie e Biofarmaceutica dell'Università degli Studi di Bari "A.Moro"; Istituto di Biomembrane. Bioenergetica e Biotecnologie Molecolari del Consiglio Nazionale delle Ricerche di Bari	Beaconlab (Bioinformatics, Evolution and Comparative Genomics lab), Dept of Biosciences, University on Milan	Chiara M; Chiara M.; Manzari C.; Parisi A.; Pesole G.
EPI_ISL_415154	KU Leuven, Clincal and Epidemiological Virology	KU Leuven, Clincal and Epidemiological Virology	Bert Vanmechelen; Joan Marti-Careras; Marc Van Ranst; Piet Maes.; Tony Wawina
EPI_ISL_415157, EPI_ISL_416467, EPI_ISL_418796, EPI_ISL_418797, EPI_ISL_418806, EPI_ISL_418986, EPI_ISL_420332, EPI_ISL_420335, EPI_ISL_420337, EPI_ISL_420341, EPI_ISL_420345, EPI_ISL_420350, EPI_ISL_420366, EPI_ISL_420367			
see above	KU Leuven, Clinical and Epidemiological Virology	KU Leuven, Clinical and Epidemiological Virology	Bert Vanmechelen; Joan Marti-Carreras; Piet Maes; Tony Wawina
EPI_ISL_458157, EPI_ISL_458168, EPI_ISL_462235	KU Leuven, Rega Institute, Clinical and Epidemiological Virology	KU Leuven, Rega Institute, Clinical and Epidemiological Virology	Bert Vanmechelen; Joan Marti-Carerras; Piet Maes; Tony Wawina-Bokalanga
EPI_ISL_444492	Karolinska Universitetslaboratoriet	CTMR, Karolinska Institutet, Stockholm, Sweden	Caroline Bjurnemark; Fredrik Boulund; Jing Wang; Jingkai Ji; Lars Engstrand; Linnéa Påvénius; Marica Hamsten; Shuiqin Li; Stefanie Prast-Nielsen; Vivien Lan Yang Swartz; Yinghua Zha; Yue Hu
EPI_ISL_455887, EPI_ISL_475141	Karolinska Universitetslaboratoriet	The Public Health Agency of Sweden	Anna Risberg; Anna-Malin Linde; Karin Tegmark-Wisell; Maria Lind Karlberg; Mattias Haukland; Olov Svartstrom; Oskar Karlsson Lindsjo; Petra Edquist; Reza Advani; Sandra Broddesson; Shamam Muradrasoli
EPI_ISL_475913	Klinikum Wels-Grieskirchen	Bergthaler laboratory, CeMM Research Center for Molecular Medicine of the Austrian Academy of Sciences	Alexander Lercher; Alexandra Popa; Andreas Bergthaler; Benedikt Agerer; Christoph Bock; Daniela Schmid; Dorothee von Laer; Elisabeth Puchhammer-Stoeckl; Franz Allerberger; Gregor Hörmann; Guenter Weiss; Henrique Colaco; Jakob-Wendelin Genger; Jan Laine; Judith Aberle; Kinga Rigler-Hohenwarter; Lukas Endler; Manfred Nairz; Mark Smyth; Martin Senekowitsch; Michael Schuster; Peter Hufnagl; Rainer Gattringer; Stephan Aberle; Thomas Penz; Wegene Borena
EPI_ISL_475147, EPI_ISL_510818	Klinisk mikrobiologi Vasternorrland	The Public Health Agency of Sweden	Anna Risberg; Anna-Malin Linde; Karin Tegmark-Wisell; Maria Lind Karlberg; Mattias Haukland; Mia Brytting; Olov Svartstrom; Oskar Karlsson Lindsjo; Petra Edquist; Reza Advani; Sandra Broddesson; Shamam Muradrasoli
EPI_ISL_510822	Klinisk mikrobiologi centralsjukhuset Karlstad	The Public Health Agency of Sweden	Anna Risberg; Anna-Malin Linde; Karin Tegmark-Wisell; Maria Lind Karlberg; Mattias Haukland; Mia Brytting; Olov Svartstrom; Oskar Karlsson Lindsjo; Petra Edquist; Reza Advani; Sandra Broddesson
EPI_ISL_428201, EPI_ISL_549024	Klinisk mikrobiologi, Region Västerbotten	Unit for Biological Agents, Department for CBRN Defence and Security, Swedish Defence Research Agency	FOI Bioinformatics team
EPI_ISL_425064	Lab voor klinische biologie	Onderzoeksgroep Virologie	Bruno Verhasselt; Hans Nauwynck; Jozefien De Clercq; Laurens Lambrechts; Linos Vandekerckhove; Marthe Pauwels; Nick Vereecke; Sebastiaan Theuns
EPI_ISL_421762, EPI_ISL_429711, EPI_ISL_429778, EPI_ISL_434493, EPI_ISL_434512	Laboratoire National de Sante, Microbiology, Virology	Laboratoire National de Sante, Microbiology, Epidemiology and Microbial Genomics	Anke Wienecke-Baldacchino; Ardashel Latsuzbaia; Catherine Ragimbeau; Guillaume Fournier; Jessica Tapp; Joel Mossong; Tamir Abdelrahman; Trung Nguyen Nguyen
EPI_ISL_419603	Laboratoire National de Santé, Microbiology, Virology	Laboratoire National de Santé, Microbiology, Epidemiology and Microbial Genomics	Anke Wienecke-Baldacchino; Ardashel Latsuzbaia; Catherine Ragimbeau; Guillaume Fournier; Jessica Tapp; Joel Mossong; Tamir Abdelrahman; Trung Nguyen Nguyen
EPI_ISL_548202, EPI_ISL_548204, EPI_ISL_548209, EPI_ISL_548214, EPI_ISL_548218, EPI_ISL_548223, EPI_ISL_548228, EPI_ISL_548229, EPI_ISL_548240			
see above	Laboratoire de Virologie, HUG	Swiss National Reference Centre for Influenza	LAUBSCHER F.
EPI_ISL_458084	Laboratorio Biologia Molecolare Sars Cov2 - UOC Laboratorio Analisi - Servizio Medicina di Laboratorio, Ospedale "San Francesco" - ATS-ASSL Nuoro	Laboratorio specialistico UOC Ematologia - Ospedale "San Francesco" - ATS-ASSL Nuoro	Aspironi Rosanna; Casu Gavino; Fancello Tatiana; Fiamma Maura; Floris Anna Rita; Lo Maglio Iana; Mameli Giuseppe.; Monne Maria Itria; Palmas Angelo Domenico; Piras Giovanna; Sanna Filomena; Sulis Vincenzo; Toja Alessandro
EPI_ISL_455468	Laboratory for Respiratory Viruses, Cantacuzino National Military-Medical Institute for Research and Development	Cantacuzino Institute	A.Cretu; L.Ustea; M.Lazar; Tim Durfee
EPI_ISL_417447	Laboratory of Infectious Diseases, Department of Biomedical and Clinical Sciences L. Sacco, University of Milan	Laboratory of Infectious Diseases, Department of Biomedical and Clinical Sciences L. Sacco, University of Milan	Agostino Riva; Alessia Lai; Annalisa Bergna; Arianna Gabrieli; Claudia Balotta; Dario Bernacchia; Gianguglielmo Zehender; Giuliano Rizzardini; Luca Meroni; Maciej Tarkowski; Massimo Galli; Spinello Antinori; Stefano Rusconi
EPI_ISL_437876, EPI_ISL_437890, EPI_ISL_437894, EPI_ISL_437901, EPI_ISL_437902, EPI_ISL_437903, EPI_ISL_437911			
see above	Laboratory of Microbiology, Medical School, National and Kapodistrian University of Athens	Laboratory of Biology, Department of Medicine, Democritus University of Thrace	Bampali, M.; Dovrolis, N.; Froukala, E.; Gatzidou, E.; Kassela K.; N. and Karakasiliotis, I.; Spanakis; Stavropoulou, A.; Tsakris, A.; Veletza, S.
EPI_ISL_498558, EPI_ISL_498559, EPI_ISL_498562	Laboratory of Molecular Virology International Center for Genetic Engineering and Biotechnology (ICGEB)	ARGO Open Lab Platform for Genome Sequencing	Dal Monego S; D'Agaro P; Licastro D; Marcello A; Rajasekharan S; Segat L
EPI_ISL_417419, EPI_ISL_417423	Laboratory of Molecular Virology International Center for Genetic Engineering and Biotechnology (ICGEB)	ARGO Open Lab Platform for Genome sequencing	D'Agaro P; Dal Monego S; Licastro D; Marcello A; Rajasekharan; Rajasekharan S; Segat L
EPI_ISL_417418	Laboratory of Molecular Virology International Center fro Genetic Engineering and Biotechnology (ICGEB)	ARGO Open Lab Platform for Genome sequencing	D'Agaro P; Dal Monego S; Licastro D; Marcello A; Rajasekharan S; Segat L
EPI_ISL_479616, EPI_ISL_479618, EPI_ISL_479790, EPI_ISL_479791, EPI_ISL_525495	Laboratory of Molecular Virology of the International Centre for Genetic Engineering and Biotechnology (ICGEB)	ARGO Open Lab Platform for Genome Sequencing	Confalonieri M; Confalonieri M Marcello A; Confalonieri P; D; D'Agaro P; Dal Monego S; Licastro; Licastro D; Marcello A; Rajasekharan S; Salton F; Segat L
EPI_ISL_437089, EPI_ISL_437090, EPI_ISL_486425, EPI_ISL_486428, EPI_ISL_486433, EPI_ISL_486435, EPI_ISL_486436			
see above	Latvijas Infektoloģijas centrs	Latvian Biomedical Research and Study Centre	Ivars Silamiķelis; Jeļena Storoženko; Jānis Kloviņš; Kaspars Megnis; Monta Ustinova; Oksana Savicka; Tatjana Kolupajeva; Uga Dumpis; Vita Rovīte; Ņikita Zrelovs
EPI_ISL_555617, EPI_ISL_567782	Lighthouse Lab in Alderley Park	Wellcome Sanger Institute for the COVID-19 Genomics UK (COG-UK) consortium	Cordelia Langford; David K. Jackson; Dominic Kwiatkowski; Ewan Harrison; Ian Johnston; Jacquelyn Wynn; John Sillitoe on behalf of the Wellcome Sanger Institute COVID-19 Surveillance Team; John Sillitoe on behalf of the Wellcome Sanger Institute COVID-19 Surveillance Team (http://www.sanger.ac.uk/covid-team); Mairead Hyland; Roberto Amato; Sonia Goncalves; The Lighthouse Lab in Alderley Park and Alex Alderton
EPI_ISL_531269, EPI_ISL_532676, EPI_ISL_532897, EPI_ISL_540045, EPI_ISL_540357	Lighthouse Lab in Glasgow	Wellcome Sanger Institute for the COVID-19 Genomics UK (COG-UK) consortium	Anna Dominiczak and Alex Alderton; Carol Clugston; Cordelia Langford; David Gray; David K. Jackson; Dominic Kwiatkowski; Ewan Harrison; Harper VanSteenhouse; Ian Johnston; John Sillitoe; John Sillitoe on behalf of the Wellcome Sanger Institute COVID-19 Surveillance Team; Roberto Amato; Sonia Goncalves; Yumi Kasai
EPI_ISL_554313, EPI_ISL_558242	Lighthouse Lab in Milton Keynes	Wellcome Sanger Institute for the COVID-19 Genomics UK (COG-UK) consortium	Cordelia Langford; David K. Jackson; Dominic Kwiatkowski; Ewan Harrison; Ian Johnston; John Sillitoe on behalf of the Wellcome Sanger Institute COVID-19 Surveillance Team; Roberto Amato; Sonia Goncalves; The Lighthouse Lab in Alderley Park and Alex Alderton
EPI_ISL_490410, EPI_ISL_490415, EPI_ISL_499645, EPI_ISL_499677, EPI_ISL_499679, EPI_ISL_499725, EPI_ISL_500050, EPI_ISL_500056, EPI_ISL_500112, EPI_ISL_517095, EPI_ISL_517196, EPI_ISL_517252, EPI_ISL_517432			
see above	Liverpool Clinical Laboratories	COVID-19 Genomics UK (COG-UK) Consortium	A Alrezaihi; Alessandro Gerada; Alistair Darby; Angela Cowell; Anita Lucaci; Anu Chawla; Cassie Olateju; Catherine Hartley; Charlotte Nelson; Ecaterina Vamos; Elaine O'Toole; Eleanor G Bentley; Ghada T Shawli; Isabel García-Dorival; James Johnson; James P Stewart; Jenifer Manson; Joanne Watts; Jones Benjamin; Jordan J Clark; Julian Hiscox; L Luu; Lucille Rainbow; M Almsaud; Margaret Hughes; Mark Whitehead; Matthew Gemmell; Miren Iturriza-Gomara; Muhannad Alruwaili; N.P Randle; Neil Swainston; PKF Gilmore; Parul Sharma; Rebekah Penrice-Randal; Richard Eccles; Richard Gregory; Sam Haldenby; Steve Paterson; Stuart D Armstrong; Trevor Ian Robinson; Ximeng Han
EPI_ISL_568898, EPI_ISL_568909, EPI_ISL_568924, EPI_ISL_568925, EPI_ISL_568926, EPI_ISL_569031, EPI_ISL_569262, EPI_ISL_569274, EPI_ISL_569294, EPI_ISL_569296, EPI_ISL_569324, EPI_ISL_569345, EPI_ISL_569358, EPI_ISL_569375, EPI_ISL_569403, EPI_ISL_569449, EPI_ISL_569563, EPI_ISL_569590			
see above	MEPHI, Aix Marseille University	MEPHI, Aix Marseille University	Anthony LEVASSEUR
EPI_ISL_413571	MHC Brabant Zuidoost	Erasmus Medical Center	Anne van der Linden; Annemiek van der Eijk; Aura Timen; Bas Oude Munnink; Claudia Schapendonk; Corien Swaan; Corine GeurtsvanKessel; David Nieuwenhuijse; Irina Chestakova; Jeroen van Kampen; Jolanda Voermans; Madelief Mollers; Manon Haverkate; Marion Koopmans; Mark Pronk; Mart Stein; Pascal Lexmond; Reina Sikkema; Richard Molenkamp; Sandra Kengne Kamga Mobou; on behalf of the Dutch national COVID-19 response team.
EPI_ISL_420912	Max von Pettenkofer Institute, Virology, National Reference Center for Retroviruses, LMU Munich	Laboratory for Functional Genome Analysis, Dept. Genomics, Gene Center of the LMU Munich	Alexander Graf; Ashok Varadharajan; Helmut Blum; Max Muenchhoff; Oliver Keppler; Stefan Krebs
EPI_ISL_437242, EPI_ISL_466893, EPI_ISL_466900	Max von Pettenkofer Institute, Virology, National Reference Center for Retroviruses, LMU München	Laboratory for Functional Genome Analysis, Dept. Genomics, Gene Center of the LMU Munich	Alexander Graf; Helmut Blum; Max Muenchhoff; Oliver Keppler; Stefan Krebs
EPI_ISL_549070	Medical Microbiology Unit, Department for Laboratory Medicine, Drammen Hospital, Vestre Viken Health Trust,	Norwegian Institute of Public Health, Department of Virology	Hilde Elshaug; Hilde Synnøve Vollan; Kamilla Heddeland Instefjord; Karoline Bragstad; Kathrine Stene-Johansen; Olav Hungnes; Rasmus Riis Kopperud
EPI_ISL_547437	Microbiology, Department of Pathology, St. Bernard's Hospital, Gibraltar Health Authority	Respiratory Virus Unit, Microbiology Services Colindale, Public Health England	Charlotte Gillborn-Jones (Gibraltar); Dr Nicholas Cortes (Gibraltar); PHE Covid Sequencing Team
EPI_ISL_486646, EPI_ISL_486650, EPI_ISL_486651, EPI_ISL_486653, EPI_ISL_486658, EPI_ISL_486659, EPI_ISL_486664			
see above	Microbiology, Virology and Biemergency Laboratory-ASST FBF Sacco	Microbiology, Virology and Biemergency Laboratory-ASST FBF Sacco	Comandatore F; Mancon A; Micheli V; Rimoldi SG; Romeri F
EPI_ISL_451306, EPI_ISL_451308, EPI_ISL_460081	Molecular Virology Unit, Fondazione IRCCS Policlinico San Matteo , Pavia	Laboratory of Virology, INMI Lazzaro Spallanzani IRCCS	Antonino Di Caro; Antonio Piralla; Barbara Bartolini; Cesare E.M. Gruber; Fausto Baldanti; Maria R. Capobianchi; Martina Rueca
EPI_ISL_426892, EPI_ISL_426893, EPI_ISL_426895	Motol University Hospital	Institute of Applied Biotechnologies a.s.	Adam Novotný; Jan Geryk; Kateřina Kvapilová; Martin Kašný; Milan Macek; Pavel Dřevínek; Petr Brož; Petr Klempt; Petr Kvapil

EPI_ISL_510815	NA	The Public Health Agency of Sweden	Anna Risberg; Anna-Malin Linde; Karin Tegmark-Wisell; Maria Lind Karlberg; Mattias Haukland; Mia Brytting; Olov Svartstrom; Oskar Karlsson Lindsjo; Petra Edquist; Reza Advani; Sandra Broddesson
EPI_ISL_459560, EPI_ISL_459580, EPI_ISL_459581, EPI_ISL_459589, EPI_ISL_459595, EPI_ISL_459598, EPI_ISL_459603, EPI_ISL_459613, EPI_ISL_459635, EPI_ISL_459689, EPI_ISL_459690, EPI_ISL_459703, EPI_ISL_459708, EPI_ISL_459709, EPI_ISL_459711, EPI_ISL_459714, EPI_ISL_469931, EPI_ISL_469945, EPI_ISL_469947, EPI_ISL_469971, EPI_ISL_469984, EPI_ISL_469990, EPI_ISL_470005, EPI_ISL_470009, EPI_ISL_489480, EPI_ISL_489584, EPI_ISL_489603, EPI_ISL_489619, EPI_ISL_489624, EPI_ISL_489656, EPI_ISL_489679, EPI_ISL_489701, EPI_ISL_489704, EPI_ISL_524545, EPI_ISL_524552, EPI_ISL_532563, EPI_ISL_534671			
see above	NHSGGC West of Scotland Specialist Virology Centre / MRC-University of Glasgow Centre for Virus Research	Wellcome Sanger Institute for the COVID-19 Genomics UK (COG-UK) consortium	Alasdair MacLean; Alice Broos; Ana da Silva Filipe; Antonia Ho; Cordelia Langford; Daniel Mair; David K. Jackson; David L Robertson; Dominic Kwiatkowski; Elihu Aranday-Cortes; Emma Thomson and Alex Alderton; Ewan Harrison; Ian Johnston; James Shepherd; Jenna Nichols; John Sillitoe; John Sillitoe on behalf of the Wellcome Sanger Institute COVID-19 Surveillance Team (http://www.sanger.ac.uk/covid-team); Joseph Hughes; Kathy Li; Kathy Smollett; Kirstyn Brunker; Kyriaki Nomikou; Lily Tong; Marc Niebel; Natasha Jesudason; Natasha Johnson; Patawee Asamaphan; Rajiv Shah; Richard Orton; Roberto Amato; Rory Gunson; Sarah McDonald; Sonia Goncalves; Sreenu Vattipally; Stephen Carmichael; Yasmin Parr
EPI_ISL_416742	NRL for Influenza, Centrum Epidemiology and Microbiology of National Institute of Public Health, Czech Republic	Charite Universitaetsmedizin Berlin, Institute of Virology	Akexander Nagy; Barbara Muehlemann; Christian Drosten; Dusan Trnka; Helena Jirincova; Jaromira Vecerova; Jörn Beheim-Schwarzbach; Julia Schneider; Ludmila Novakova; Talitha Veith; Terry Jones; Victor M Corman
EPI_ISL_458696, EPI_ISL_488760, EPI_ISL_488788	NU-OMICS DNA Sequencing research facility, Northumbria University	Wellcome Sanger Institute for the COVID-19 Genomics UK (COG-UK) consortium	Andrew Nelson; Brendan Payne; Chris Duncan; Clive Graham; Cordelia Langford; Darren Smith and Alex Alderton; David K. Jackson; Debra Padgett; Dominic Kwiatkowski; Edward Barton; Emma Swindells; Ewan Harrison; Garren Scott; Gary Black; Gary Eltringham; Greg Young; Ian Johnston; Jane Greenaway; Jennifer Collins; John Allan; John Sillitoe on behalf of the Wellcome Sanger Institute COVID-19 Surveillance Team (http://www.sanger.ac.uk/covid-team); Joshua Loh; Lynn Dover; Matthew Bashton; Paul Baker; Roberto Amato; Sarah Essex; Sheia Waugh; Shirelle Burton-Fanning; Sonia Goncalves; Steve Ligget; Wen Yew; Yusri Taha
EPI_ISL_523960, EPI_ISL_523966, EPI_ISL_523968	National Agency for Public Health, Republic of Moldova	Charite Universitatsmedizin Berlin, Institute of Virology	Ala Halacu; Barbara Mühlemann; Christian Drosten; Jörn Beheim-Schwarzbach; Julia Schneider; Mariana Apostol; Talitha Veith; Terry Jones; Victor M Corman
EPI_ISL_467778	National Influenza Centre Romania	Charite Universitatsmedizin Berlin, Institute of Virology	Barbara Muehlemann; Christian Drosten; Jorn Beheim-Schwarzbach; Julia Schneider; L. Ustea; M. Lazar; N. Paraschiv; Talitha Veith; Terry Jones; Victor M Corman
EPI_ISL_539780, EPI_ISL_539781	National Institute of Public Health (Czech Republic)	State Veterinary Institute Prague	A; D; H; Jirincova; L; Nagy; Novakova; Trnka; Vecerova, J.
EPI_ISL_512597, EPI_ISL_512603, EPI_ISL_512605, EPI_ISL_512606, EPI_ISL_512607, EPI_ISL_512608, EPI_ISL_512609, EPI_ISL_512611, EPI_ISL_512617, EPI_ISL_512618, EPI_ISL_512622, EPI_ISL_512640, EPI_ISL_512641, EPI_ISL_512810			
see above	National Laboratory for Influenza/Virology reference laboratory, Public Health Center of the Ministry of Health of Ukraine	Respiratory Virus Unit, Microbiology Services Colindale, Public Health England	Dr. Iryna Demchyshyna; PHE Covid Sequencing Team
EPI_ISL_480297, EPI_ISL_480299, EPI_ISL_480300, EPI_ISL_480307, EPI_ISL_480309, EPI_ISL_480310	National Reference Laboratory "Influenza and acute respiratory diseases"	NRL-HIV	Ivailo Alexiev; Ivan Ivanov; Ivva Philipova
EPI_ISL_516831	North West London Pathology, Imperial College Healthcare NHS Trust	Wellcome Sanger Institute for the COVID-19 Genomics UK (COG-UK) consortium	Aileen Rowan; Alison Holmes; Anjna Badhan; Carolina Herrera and Alex Alderton; Cordelia Langford; David K. Jackson; David Muir; Dominic Kwiatkowski; Ewan Harrison; Frankie Bolt; Graham Taylor; Ian Johnston; James Price; John Sillitoe on behalf of the Wellcome Sanger Institute COVID-19 Surveillance Team (http://www.sanger.ac.uk/covid-team); Ling Li; Paul Randell; Roberto Amato; Sonia Goncalves
EPI_ISL_507114, EPI_ISL_507117, EPI_ISL_507124, EPI_ISL_572950	Northumbria University / South Tees Hospitals NHS Foundation Trust / North Cumbria Integrated Care NHS Foundation Trust / North Tees and Hartlepool NHS Foundation Trust / Newcastle Hospitals NHS Foundation Trust	COVID-19 Genomics UK (COG-UK) Consortium	Andrew Nelson; Brendan Payne; Clive Graham; Darren L Smith; Debra Padgett; Edward Barton; Emma Swindells; Garren Scott; Gary Black; Gary Eltringham; Giles S Holt; Greg R Young; Jane Greenaway; Jennifer Collins; John Allan; Joshua Loh; Lynn Dover; Matthew Bashton; Mohammad A Tariq; Paul Baker; Sarah Essex; Steve Liggett; Wen C Yew; Yusri Taha
EPI_ISL_569741, EPI_ISL_569780, EPI_ISL_569788	Omsk Research Institute of Natural Focal Infections	WHO National Influenza Centre Russian Federation	Aleksei Vasilenko; Andrey Komissarov; Artem Fadeev; Daria Nashatyreva; Ekaterina Gradoboeva; Ekaterina Savkina; Elena Poleshchuk; Valery Yakimenko
EPI_ISL_493352	Oslo University Hospital, Department of Medical Microbiology	Norwegian Institute of Public Health, Department of Virology	Hilde Elshaug; Kamilla Heddeland Instefjord; Karoline Bragstad; Kathrine Stene-Johansen; Olav Hungnes; Rasmus Riis Kopperud
EPI_ISL_529014	Ospedale "Ss. Annunziata"	Istituto Zooprofilattico Sperimentale dell'Abruzzo e Molise "G.Caporale"	Ancora M; Cammà C; Curini V; Di Domenico M; Di Pasquale A; Lorusso A; Mangone I; Marcacci M; Puglia I; Rinaldi A; Savini G.
EPI_ISL_420565	Ospedale Civile Giuseppe Mazzini	Istituto Zooprofilattico Sperimentale dell'Abruzzo e Molise "G.Caporale"	Ancora M; Cammà C; Curini V; Di Domenico M; Di Pasquale A; Lorusso A; Mangone I; Marcacci M; Puglia I; Rinaldi A; Savini G
EPI_ISL_418257	Ospedale Civile Giuseppe Mazzini, Teramo	Istituto Zooprofilattico Sperimentale dell'Abruzzo e Molise "G.Caporale"	Ancora M; Cammà C; Curini V; Di Domenico M; Di Pasquale A; Lorusso A; Mangone I; Marcacci M; Puglia I; Rinaldi A; Savini G.
EPI_ISL_528990	Ospedale Civile Maria SS. dello Splendore	Istitituto Zooprofilattico Sperimentale dell'Abruzzo e Molise "G.Caporale"	Ancora M; Cammà C; Curini V; Di Domenico M; Di Pasquale A; Lorusso A; Mangone I; Marcacci M; Puglia I; Rinaldi A; Savini G.
EPI_ISL_429236	Ospedale Civile S. Liberatore di Atri	Istituto Zooprofilattico Sperimentale dell'Abruzzo e Molise "G. Caporale"	Ancora M; Camma C; Curini V; Di Domenico M; Di Pasquale A; Lorusso A; Mangone I; Marcacci M; Puglia I; Rinaldi A; Savini G
EPI_ISL_436719	Ospedale Civile S. Liberatore di Atri	Istituto Zooprofilattico Sperimentale dell'Abruzzo e Molise "G.Caporale"	Ancora M; Cammà C; Curini V; Di Domenico M; Di Pasquale A; Lorusso A; Mangone I; Marcacci M; Puglia I; Rinaldi A; Savini G
EPI_ISL_529009	Ospedale Civile S. Liberatore-Atri	Istitituto Zooprofilattico Sperimentale dell'Abruzzo e Molise "G.Caporale"	Ancora M; Cammà C; Curini V; Di Domenico M; Di Pasquale A; Lorusso A; Mangone I; Marcacci M; Puglia I; Rinaldi A; Savini G.
EPI_ISL_436718	Ospedale Regionale San Salvatore	Istituto Zooprofilattico Sperimentale dell'Abruzzo e Molise "G.Caporale"	Ancora M; Cammà C; Curini V; Di Domenico M; Di Pasquale A; Lorusso A; Mangone I; Marcacci M; Puglia I; Rinaldi A; Savini G
EPI_ISL_435148	Ospedale SS Annunziata	Istituto Zooprofilattico Sperimentale dell'Abruzzo e Molise "G.Caporale"	Ancora M; Cammà C; Curini V; Di Domenico M; Di Pasquale A; Lorusso A; Mangone I; Marcacci M; Puglia I; Rinaldi A; Savini G
EPI_ISL_442665, EPI_ISL_442745, EPI_ISL_443752, EPI_ISL_443757, EPI_ISL_443783, EPI_ISL_443855, EPI_ISL_443891, EPI_ISL_443909, EPI_ISL_488388, EPI_ISL_492536, EPI_ISL_492548, EPI_ISL_492678, EPI_ISL_492729, EPI_ISL_492764, EPI_ISL_495098, EPI_ISL_495103, EPI_ISL_495116			
see above	PHE South West Regional Laboratory, National Infection Service	Wellcome Sanger Institute for the COVID-19 Genomics UK (COG-UK) consortium	Alex Alderton; Barry Vipond; Cordelia Langford; David K. Jackson; Dominic Kwiatkowski; Dr Peter Muir; Ewan Harrison; Hannah Pymont; Ian Johnston; John Sillitoe on behalf of the Wellcome Sanger Institute COVID-19 Surveillance Team (http://www.sanger.ac.uk/covid-team); Rich Hopes; Roberto Amato; Sonia Goncalves; Stephanie Hutchings; and Alex Alderton
EPI_ISL_528920	Presidio Ospedaliero "Santo Spirito"-Pescara	Istitituto Zooprofilattico Sperimentale dell'Abruzzo e Molise "G.Caporale"	Ancora M; Cammà C; Curini V; Di Domenico M; Di Pasquale A; Lorusso A; Mangone I; Marcacci M; Puglia I; Rinaldi A; Savini G.
EPI_ISL_449017, EPI_ISL_449097, EPI_ISL_453550, EPI_ISL_453572	Quadram Institute Bioscience	COVID-19 Genomics UK (COG-UK) Consortium	Alexander J Trotter; Alison E. Mather; Alp Aydin; Ana P. Tedim; Anastasia Kolyva; Andrew Bell; Andrew J. Page; Claire Stuart; Dave J. Baker; Gemma L. Kay; John Wain; Justin O'Grady; Leonardo de Oliveira Martins; Lizzie Meadows; Maria Diaz; Mark Webber; Muhammed Yasir; Nabil-Fareed Alikhan; Ngozi Elumogo; Nicholas M. Thomson; Rachael Stanley; Rachel Gilroy; Reenesh Prakash; Samir Dervisevic; Samuel Bloomfield; Steven Rudder; Thanh Le-Viet
EPI_ISL_425570	Queens Medical Centre, Clinical Microbiology Department / DeepSeq Nottingham	COVID-19 Genomics UK (COG-UK) Consortium	Christopher Moore; Fei Sang; Gemma Clark; Hannah Howson-Wells; Johnny Debebe; Jonathan Ball; Joseph Chappell; Manjinder Khakh; Matthew Carlisle; Matthew Loose; Nadine Holmes; Patrick McClure; Theocharis Tsoleridis; Victoria Wright; Wendy Smith
EPI_ISL_441381	Regional Virus Laboratory, Belfast Health and Social Care Trust	COVID-19 Genomics UK (COG-UK) Consortium	Alison Watt; Ciara Cox; Conall McCaughey; David Simpson; Derek Fairley; James McKenna; Mairead Connor; Susan Feeney; Tanya Curran; Zoltan Molnar
EPI_ISL_441693, EPI_ISL_441696, EPI_ISL_441697, EPI_ISL_441708, EPI_ISL_441730, EPI_ISL_441733, EPI_ISL_459326, EPI_ISL_459341, EPI_ISL_469854, EPI_ISL_469857, EPI_ISL_469862, EPI_ISL_469872, EPI_ISL_469880, EPI_ISL_470016, EPI_ISL_470024, EPI_ISL_470034, EPI_ISL_470050, EPI_ISL_470072, EPI_ISL_470076, EPI_ISL_470077, EPI_ISL_470084, EPI_ISL_482041, EPI_ISL_482052, EPI_ISL_489196, EPI_ISL_489201, EPI_ISL_489202, EPI_ISL_489212, EPI_ISL_489301, EPI_ISL_489311, EPI_ISL_489314, EPI_ISL_489356, EPI_ISL_500974, EPI_ISL_501036, EPI_ISL_501038			
see above	Regional Virus Laboratory, Belfast Health and Social Care Trust	Wellcome Sanger Institute for the COVID-19 Genomics UK (COG-UK) consortium	Alex Alderton; Alison Watt; Ciara Cox; Conall McCaughey; Cordelia Langford; David K. Jackson; David Simpson; Derek Fairley; Dominic Kwiatkowski; Ewan Harrison; Ian Johnston; James McKenna; John Sillitoe on behalf of the Wellcome Sanger Institute COVID-19 Surveillance Team (http://www.sanger.ac.uk/covid-team); Mairead Connor; Roberto Amato; Sonia Goncalves; Susan Feeney; Tanya Curran; Zoltan Molnar; and Alex Alderton
EPI_ISL_414523, EPI_ISL_414526, EPI_ISL_417255, EPI_ISL_418702, EPI_ISL_464194, EPI_ISL_464215, EPI_ISL_464450, EPI_ISL_464456, EPI_ISL_464997			
see above	Respiratory Virus Unit, Microbiology Services Colindale, Public Health England	Respiratory Virus Unit, Microbiology Services Colindale, Public Health England	Angie Lackenby; Joanna Ellis; Jonathan Hubb; Kirstin Edwards; Leena Bhaw; Maria Zambon; Monica Galiano; Omolola Akinbami; PHE Covid Sequencing Team; Richard Myers; Shahjahan Miah; Steven Platt; Tiina Talts
EPI_ISL_457722, EPI_ISL_457727, EPI_ISL_457729	SYNLAB Eesti OÜ	Charite Universitatsmedizin Berlin, Institute of Virology	Barbara Mühlemann; Christian Drosten; Jorn Beheim-Schwarzbach; Julia Schneider; Paul Naaber; Talitha Veith; Terry Jones; Victor M Corman
EPI_ISL_455733	Servicio de Microbiologia. Hospital Clinico Universitario de Valencia	Sequencing and Bioinformatics Service and Molecular Epidemiology Research Group. FISABIO-Public Health, and SeqCOVID-Spain Consortium	David Navarro; Eliseo Albert; Fernando Gonzalez-Candelas; Giuseppe 'Auria; Inma Galán Vendrell; Ivan Ansari; Lidia Ruiz Roldan; Llúcia Martínez-Priego; Loreto Ferrús Abad; Maria Alma Bracho; Mariana Reyes-Prieto; Neris Garcia-Gonzalez; Paula Ruiz-Hueso; Sandra Carbo; Vicente Soriano Chirona
EPI_ISL_541949	Servicio de Microbiología, Hospital Universitario Son Espases	SeqCOVID-SPAIN consortium/IBV(CSIC)	Antonio Oliver and SeqCOVID-SPAIN consortium; Carla López-Causapé; Jordi Reina
EPI_ISL_510506	Servicio de Microbiología. Hospital Universitario Donostia. OSI Donostialdea. Área de Enfermedades Infecciosas, Grupo de Infección Respiratoria y Resistencia Antimicrobiana. Instituto de Investigación Sanitaria Biodonostia	SeqCOVID-SPAIN consortium/IBV(CSIC)	Gustavo Cilla; Jose Maria Marimón and SeqCOVID-SPAIN consortium; Luis Piñeiro; Milagrosa Montes
EPI_ISL_528994, EPI_ISL_528995, EPI_ISL_528996, EPI_ISL_528997, EPI_ISL_529000, EPI_ISL_529003, EPI_ISL_529005			
see above	Servizio di igiene epidemiologia e sanità pubblica (SIESP)-Chieti	Istitituto Zooprofilattico Sperimentale dell'Abruzzo e Molise "G.Caporale"	Ancora M; Cammà C; Curini V; Di Domenico M; Di Pasquale A; Lorusso A; Mangone I; Marcacci M; Puglia I; Rinaldi A; Savini G.
EPI_ISL_475106, EPI_ISL_475107	Skovde/Unilabs	The Public Health Agency of Sweden	Anna Risberg; Anna-Malin Linde; Karin Tegmark-Wisell; Maria Lind Karlberg; Mattias Haukland; Olov Svartstrom; Oskar Karlsson Lindsjo; Petra Edquist; Reza Advani; Sandra Broddesson; Shamam Muradrasoli
EPI_ISL_451655, EPI_ISL_451656, EPI_ISL_451658, EPI_ISL_451659, EPI_ISL_451660, EPI_ISL_451662, EPI_ISL_451664			
see above	State Sanitary Inspectorate	Laboratory of Recombinant Vaccines	Boguslaw Szewczyk; Jaroslaw Pinkas; Krystyna Bienkowska-Szewczyk; Lukasz Rabalski
EPI_ISL_491046, EPI_ISL_491058, EPI_ISL_491060, EPI_ISL_491067, EPI_ISL_491072, EPI_ISL_491087	Suceava County Emergency Hospital	"Stefan cel Mare" University Metagenomics Lab	Antoniadis Panagiotis et al.; Lobiuc Andrei; Lobiuc Andrei et al.
EPI_ISL_475564	Surbrunns VC	The Public Health Agency of Sweden	Anna Risberg; Anna-Malin Linde; Karin Tegmark-Wisell; Maria Lind Karlberg; Mattias Haukland; Mia Brytting; Olov Svartstrom; Oskar Karlsson Lindsjo; Reza Advani; Sandra Broddesson
EPI_ISL_491092, EPI_ISL_530347, EPI_ISL_530348	The National Institute of Public Health	State Veterinary Institute Prague	A; D; H; J; Jirincova; L; Nagy; Novakova; Trnka; Vecerova

EPI_ISL_471528, EPI_ISL_471530, EPI_ISL_471540, EPI_ISL_471544, EPI_ISL_471553, EPI_ISL_476067	The National Institute of Public Health	State Veterinary Institute Prague and The National Institute of Public Health	A; D; H; J; Jirincova; L; Nagy; Novakova; Trnka; Vecerova
EPI_ISL_489709, EPI_ISL_490112, EPI_ISL_491095, EPI_ISL_491117, EPI_ISL_491118, EPI_ISL_513511	The National Institute of Public Health	The National Institute of Public Health and State Veterinary Institute Prague	A; D; H; J; Jirincova; L; Nagy; Novakova; Trnka; Vecerova
EPI_ISL_417589, EPI_ISL_417815, EPI_ISL_417831, EPI_ISL_424384, EPI_ISL_424617	The National University Hospital of Iceland	deCODE genetics	Agnar Helgason; Alma Moller; Arna B Agustsdottir; Arnaldur Gylfason; Asgeir Sigurdsson; Aslaug Jonasdottir; Berglind Eiriksdottir; Bjarni Thorbjornsson; Brynjar O Jensson; Daniel F Gudbjartsson; Droplaug N Magnusdottir; Elisabet E Gardarsdottir; Emil A Thorarensen; Gardar Sveinbjornsson; Gisli Masson; Gudmundur Georgsson; Gudmundur L Norddahl; Gudrun Sigmundsdottir; Hakon Jonsson; Hilma Holm; Ingileif Jonsdottir; Jona Saemundsdottir; Kamilla S Josefsdottir; Kari Stefansson; Karl G Kristinsson; Kjartan R Gudmundsson; Kristin E Sveinsdottir; Louise le Roux; Maney Sveinsdottir; Olafia S Gretarsdottir; Olafur T Magnusson; Pall Melsted; Patrick Sulem; Run Fridriksdottir; Thora R Gunnarsdottir; Thordur Kristjansson; Thorolfur Gudnason; Unnur Thorsteinsdottir
EPI_ISL_419693	The Republican Research and Practical Center for Epidemiology and Microbiology	Charité Universitätsmedizin Berlin, Institute of Virology	Barbara Mühlemann; Christian Drosten; Jörn Beheim-Schwarzbach; Julia Schneider; Natallia Shmialiova; Natallia Sivets; Talitha Veith; Terry Jones; Victor M Corman
EPI_ISL_414586, EPI_ISL_418581, EPI_ISL_418584	UCD National Virus Reference Laboratory	UCD National Virus Reference Laboratory	Alison Murphy; Brendan Loftus; Cillian F De Gascun; Gabriel Gonzalez; Jeff Connell; Jonathan Dean; Ken Wolfe; Kevin Byrne; Michael Carr; Suzie Coughlan
EPI_ISL_453851	ULS Litoral Alentejano	Instituto Nacional de Saude (INSA)	Borges et al
EPI_ISL_452182, EPI_ISL_452184, EPI_ISL_452189, EPI_ISL_522855, EPI_ISL_522860, EPI_ISL_522864, EPI_ISL_522865, EPI_ISL_522866			
see above	ULSS9 Distretto di Bussolengo	Istituto Zooprofilattico Sperimentale delle Venezie	Adelaide Milani; Alessia Schivo; Alice Fusaro; Ambra Pastori; Annalisa Salviato; Antonia Ricci; Bianca Zecchin; Calogero Terregino; Erika Giorgia Quaranta; Isabella Monne
EPI_ISL_522858	ULSS9 Distretto di San Bonifacio	Istituto Zooprofilattico Sperimentale delle Venezie	Adelaide Milani; Alessia Schivo; Alice Fusaro; Ambra Pastori; Annalisa Salviato; Antonia Ricci; Bianca Zecchin; Calogero Terregino; Erika Giorgia Quaranta; Isabella Monne
EPI_ISL_522859	ULSS9 Scaligera	Istituto Zooprofilattico Sperimentale delle Venezie	Adelaide Milani; Alessia Schivo; Alice Fusaro; Ambra Pastori; Annalisa Salviato; Antonia Ricci; Bianca Zecchin; Calogero Terregino; Erika Giorgia Quaranta; Isabella Monne
EPI_ISL_510530	UMR 190 - Faculte de medecine, UMR 'Emergence des Pathologies Virales' (EPV: Aix-Marseille University - IRD 190 - Inserm 1207 - EHE	UMR 190 - Faculte de medecine, UMR 'Emergence des Pathologies Virales' (EPV: Aix-Marseille University - IRD 190 - Inserm 1207 - EHE	De Laval, F.; Durand; F.-X.; G.A.; Geulen, M.; Gilles, M.; Le Flem; Leparc Goffart, I.; Peduzzi, F.; Piorkowski, G.; Pommier De Santi, V.
EPI_ISL_511160	UNILABS	Instituto Nacional de Saude (INSA)	Borges et al
EPI_ISL_475122, EPI_ISL_475126	Umea klinisk mikrobiologi	The Public Health Agency of Sweden	Anna Risberg; Anna-Malin Linde; Karin Tegmark-Wisell; Maria Lind Karlberg; Mattias Haukland; Olov Svartstrom; Oskar Karlsson Lindsjo; Petra Edquist; Reza Advani; Sandra Broddesson; Shamam Muradrasoli
EPI_ISL_440989, EPI_ISL_441016, EPI_ISL_441036, EPI_ISL_444253, EPI_ISL_449676, EPI_ISL_449698, EPI_ISL_461763, EPI_ISL_478405, EPI_ISL_478421, EPI_ISL_478453, EPI_ISL_478464, EPI_ISL_478466			
see above	University College London, Great Ormond Street Hospital for Children NHS Foundation Trust, Imperial College Healthcare NHS Trust	COVID-19 Genomics UK (COG-UK) Consortium	Alison Holmes; Charlotte Williams; Helena Tutill; Jacqueline Findlay; James Price; Judith Breuer; Julianne Brown; Kathryn Harris; Leysa Forrest; Mark Kristiansen; Paola Niola; Paola Resende Silva; Patricia Dyal; Paul Randell; Rachel Williams; Sam Weeks; Samuel Weeks; Sergi Castellano; Sunando Roy; Tony Brooks; Yasmin Panchbhaya
EPI_ISL_487630	University College London, Great Ormond Street Hospital for Children NHS Foundation Trust, Imperial College Healthcare NHS Trust	Wellcome Sanger Institute for the COVID-19 Genomics UK (COG-UK) consortium	Alison Holmes; Charlotte Williams; Cordelia Langford; David K. Jackson; Dominic Kwiatkowski; Ewan Harrison; Helena Tutill; Ian Johnston; Jacqueline Findlay; James Price; John Sillitoe on behalf of the Wellcome Sanger Institute COVID-19 Surveillance Team (http://www.sanger.ac.uk/covid-team); Judith Breuer and Alex Alderton; Julianne Brown; Kathryn Harris; Leysa Forrest; Mark Kristiansen; Paola Niola; Paola Resende Silva; Patricia Dyal; Paul Randell; Rachel Williams; Roberto Amato; Sam Weeks; Sergi Castellano; Sonia Goncalves; Sunando Roy; Tony Brooks; Yasmin Panchbhaya
EPI_ISL_454581, EPI_ISL_454583	University Hospital for Infectious Diseases "Dr. Fran Mihaljević", Research Unit	University of Zagreb, Centre for research and knowledge transfer in biotechnology	Anamarija Slovic; Ivan-Christian Kurolt; Jelena Ivancic Jelecki
EPI_ISL_473436	University of Birmingham	COVID-19 Genomics UK (COG-UK) Consortium	Alex Richter; Andrew Bosworth; Andrew Bosworth. Queen Elizabeth Hospital: Anna Casey; Andrew D Beggs PHE Heartlands Lab: Husam Osman; Charlotte Poxon; Institute of Microbiology; Joanne Stockton; Josh Quick; Kasun Wanigasooriya; Mike Kidd; Nicholas Loman; Oliver Pickles; Radoslaw Poplawski; Samuel Nicholls; University of Birmingham Testing Laboratory: Celina M Whalley; University of Birmingham: Claire McMurray; Will Rowe
EPI_ISL_457200, EPI_ISL_457214, EPI_ISL_457251	University of Exeter	COVID-19 Genomics UK (COG-UK) Consortium	Aaron Jeffries; Audrey Farbos; Ben Temperton; Jane Masoli; Joanna Warwick-Dugdale; Michelle Michelsen; Robyn Manley; Stephen Michell
EPI_ISL_477619, EPI_ISL_477623, EPI_ISL_477624	University of Szeged, Institute of Clinical Microbiology	National Laboratory of Virology, Szentágothai Research Centre	Balázs Somogyi; Brigitta Zana; Endre Gábor Tóth; Ferenc Jakab; Gábor Kemenesi; Terhes Gabriella
EPI_ISL_475516	Uppsala Narakut Aleris	The Public Health Agency of Sweden	Anna Risberg; Anna-Malin Linde; Karin Tegmark-Wisell; Maria Lind Karlberg; Mattias Haukland; Mia Brytting; Olov Svartstrom; Oskar Karlsson Lindsjo; Reza Advani; Sandra Broddesson
EPI_ISL_475118	Uppsala klinisk mikrobiologi	The Public Health Agency of Sweden	Anna Risberg; Anna-Malin Linde; Karin Tegmark-Wisell; Maria Lind Karlberg; Mattias Haukland; Olov Svartstrom; Oskar Karlsson Lindsjo; Petra Edquist; Reza Advani; Sandra Broddesson; Shamam Muradrasoli
EPI_ISL_549173	Vestfold Hospital, Toensberg Department of Microbiology	Norwegian Institute of Public Health, Department of Virology	Hilde Elshaug; Hilde Synnøve Vollan; Kamilla Heddeland Instefjord; Karoline Bragstad; Kathrine Stene-Johansen; Olav Hungnes; Rasmus Riis Kopperud
EPI_ISL_451676, EPI_ISL_451730, EPI_ISL_466958, EPI_ISL_466959, EPI_ISL_512016, EPI_ISL_523924	Viollier AG	Department of Biosystems Science and Engineering, ETH Zürich	Christian Beisel; Christiane Beckmann; Christoph Noppen; Elodie Burcklen; Ina Nissen; Ivan Topolsky; Maurice Redondo; Natascha Santacroce; Niko Beerenwinkel; Noemie Santamaria de Souza; Olivier Kobel; Pedro Ferreira; Philipp Jablonski; Sarah Nadeau; Sophie Seidel; Susana Posada-Céspedes; Tanja Stadler; Tobias Schär
EPI_ISL_418183, EPI_ISL_435403, EPI_ISL_435418, EPI_ISL_435426, EPI_ISL_435427, EPI_ISL_435428, EPI_ISL_435430			
see above	Virological Research Group, Szentágothai Research Centre	Bioinformatics Research Group, Szentágothai Research Centre	Attila Gyenesei; Endre Gábor Tóth; Ferenc Jakab; Gábor Kemenesi; Péter Urbán; Róbert Herczeg
EPI_ISL_416426	Virological Research Group, Szentágothai Research Centre, University of Pécs	Bioinformatics Research Group, Szentágothai Research Centre, University of Pécs	Attila Gyenesei; Endre Gábor Tóth; Ferenc Jakab; Gábor Kemenesi; Péter Urbán; Róbert Herczeg
EPI_ISL_487648, EPI_ISL_487652, EPI_ISL_487697, EPI_ISL_487698, EPI_ISL_487707, EPI_ISL_487711, EPI_ISL_487715, EPI_ISL_487788, EPI_ISL_487805, EPI_ISL_487810, EPI_ISL_487874, EPI_ISL_487883, EPI_ISL_487886, EPI_ISL_487888, EPI_ISL_487933, EPI_ISL_487934, EPI_ISL_487967, EPI_ISL_488905, EPI_ISL_488943, EPI_ISL_488961, EPI_ISL_489028, EPI_ISL_489045			
see above	Virology Department, Royal Infirmary of Edinburgh, NHS Lothian / School of Biological Sciences, University of Edinburgh	Wellcome Sanger Institute for the COVID-19 Genomics UK (COG-UK) consortium	Colquhoun R; Cordelia Langford; David K. Jackson; Dewar R; Dominic Kwiatkowski; Ewan Harrison; Hill V; Ian Johnston; Jackson B; John Sillitoe on behalf of the Wellcome Sanger Institute COVID-19 Surveillance Team (http://www.sanger.ac.uk/covid-team); McCrone JT; McHugh M; O'Toole Á; Rambaut A; Roberto Amato; Rooke S; Scher E; Sonia Goncalves; Templeton K and Alex Alderton; Yu X
EPI_ISL_425836, EPI_ISL_425902, EPI_ISL_425966, EPI_ISL_433200, EPI_ISL_433430, EPI_ISL_433460, EPI_ISL_439345, EPI_ISL_453106			
see above	Virology Department, Royal Infirmary of Edinburgh, NHS Lothian / School of Biological Sciences, University of Edinburgh / Institute of Genetics and Molecular Medicine, University of Edinburgh	COVID-19 Genomics UK (COG-UK) Consortium	Balcaza C; Colquhoun R; Dewar R; Gallagher M; Hill V; Jackson B; McCrone JT; McHugh M; O'Toole A; O’Toole Á; O,ÄôToole √Å; Rambaut A; Rooke S; Scher E; Templeton K; Williams TC; Yu X
EPI_ISL_416732, EPI_ISL_416734, EPI_ISL_420161, EPI_ISL_420242	Virology Department, Sheffield Teaching Hospitals NHS Foundation Trust	Department of Infection, Immunity and Cardiovascular Disease, The Florey Institute, The Medical School, University of Sheffield	Adri Angyal; Alex Keeley; Benjamin Lindsey; Cariad Evans; Danielle Groves; Dave Partridge; Luke Green; Matthew Parker; Matthew Wyles; Mehmet Yavuz; Mohammad Raza; Paul Parsons; Rachel Tucker; Rebecca Brown; Thushan de Silva
EPI_ISL_441984, EPI_ISL_453713, EPI_ISL_453744, EPI_ISL_453769, EPI_ISL_453771	Virology Department, Sheffield Teaching Hospitals NHS Foundation Trust/Department of Infection, Immunity and Cardiovascular Disease, The Medical School, University of Sheffield	COVID-19 Genomics UK (COG-UK) Consortium	Adri Angyal; Alex Keeley; Benjamin Lindsey; Cariad Evans; Danielle Groves; Dave Partridge; Katie Johnson; Laura Carrilero; Luke Green; Matthew Parker; Matthew Wyles; Mehmet Yavuz; Mohammad Raza; Nikki Smith; Paul Parsons; Rachel Tucker; Rebecca Brown; Thushan de Silva
EPI_ISL_417491	Virology Laboratory, Department of Biomedical Sciences and Public Health, University Politecnica delle Marche	Virology and Legal Medicine Laboratories, Department of Biomedical Sciences and Public Health, University Politecnica delle Marche	Alessandrini, F.; Bagnarelli, P.; Caucci, S.; Di Sante, L.; Menzo, S.; Onofri, V.; Tagliabracci, A.; Turchi, C.
EPI_ISL_450257, EPI_ISL_450263, EPI_ISL_450264, EPI_ISL_507216, EPI_ISL_507227, EPI_ISL_507228, EPI_ISL_507233, EPI_ISL_507246, EPI_ISL_507259, EPI_ISL_507271			
see above	WHO National Influenza Centre Russian Federation	WHO National Influenza Centre Russian Federation	Andrey Komissarov; Anna Ivanova; Artem Fadeev; Daria Danilenko; Ksenia Komissarova; Mariia Pisareva; Mariia Sergeeva; Mariia Timofeeva; Tamila Musaeva; Veronica Eder
EPI_ISL_413555, EPI_ISL_415655, EPI_ISL_415656, EPI_ISL_432188, EPI_ISL_432280, EPI_ISL_445688	Wales Specialist Virology Centre	Public Health Wales Microbiology Cardiff	Alec Birchley; Alexander Adams; Amy Gaskin; Bree Gatica-Wilcox; Catherine Moore; Cen Sabu; Jason Coombes; Joanne Watkins; Johnathan Evans; Laura Gifford; Lauren Gilbert; Lee Graham; Malorie Perry; Matthew Bull; Nicole Pacchiarini; Sally Corden; Sara Kumziene-Summerhayes; Sara Rey; Sarah Taylor; Simon Cottrell; Sophie Jones; Tom Connor
EPI_ISL_574228	Wales Specialist Virology Centre Sequencing lab: Pathogen Genomics Unit	COVID-19 Genomics UK (COG-UK) Consortium	Alec Birchley; Alexander Adams; Amy Gaskin; Angela Marchbank; Bree Gatica-Wilcox; Catherine Moore; Jason Coombes; Joanne Watkins; Joel Southgate; Johnathan Evans; Laura Gifford; Lauren Gilbert; Lee Graham; Malorie Perry; Matthew Bull; Nicole Pacchiarini; Sally Corden; Sara Kumziene-Summerhayes; Sara Rey; Sarah Taylor; Simon Cottrell; Sophie Jones; Tom Connor
EPI_ISL_425674, EPI_ISL_425783, EPI_ISL_425785, EPI_ISL_425790, EPI_ISL_425803, EPI_ISL_433286, EPI_ISL_433535, EPI_ISL_433600, EPI_ISL_438776, EPI_ISL_438913, EPI_ISL_448214, EPI_ISL_461642, EPI_ISL_484614, EPI_ISL_490652			
see above	West of Scotland Specialist Virology Centre, NHSGGC / MRC-University of Glasgow Centre for Virus Research	COVID-19 Genomics UK (COG-UK) Consortium	Alasdair MacLean; Alice Broos; Ana da Silva Filipe; Antonia Ho; Daniel Mair; David L Robertson; Elihu Aranday-Cortes; Emma Thomson; James Shepherd; Jenna Nichols; Joseph Hughes; Kathy Li; Kathy Smollett; Kirstyn Brunker; Kyriaki Nomikou; Lily Tong; Marc Niebel; Natasha Jesudason; Natasha Johnson; Patawee Asamaphan; Rajiv Shah; Richard Orton; Rory Gunson; Rory Gunson.; Sarah McDonald; Sreenu Vattipally;

			Stephen Carmichael; Yasmin Parr
EPI_ISL_475891, EPI_ISL_475893, EPI_ISL_475895	Zentralinstitut für medizinische und chemische Labordiagnostik, Universitätskliniken Insbruck	Bergthaler laboratory, CeMM Research Center for Molecular Medicine of the Austrian Academy of Sciences	Alexander Lercher; Alexandra Popa; Andreas Bergthaler; Benedikt Agerer; Christoph Bock; Daniela Schmid; Dorothee von Laer; Elisabeth Puchhammer-Stoeckl; Franz Allerberger; Gregor Hörmann; Guenter Weiss; Henrique Colaco; Jakob-Wendelin Genger; Jan Laine; Judith Aberle; Kinga Rigler-Hohenwarter; Lukas Endler; Manfred Nairz; Mark Smyth; Martin Senekowitsch; Michael Schuster; Peter Hufnagl; Rainer Gattringer; Stephan Aberle; Thomas Penz; Wegene Borena
EPI_ISL_468135	[Romania, Bucharest] National Institute for Infectious Diseases "Prof. Dr. Matei Balş"	[Romania, Bucharest] National Institute for Infectious Diseases "Prof. Dr. Matei Balş"	Corina Casangiu; Leontina Banica; Marius Cotic; Marius Surleac; Simona Paraschiv
EPI_ISL_417662	deCODE genetics	deCODE genetics	Agnar Helgason; Alma Moller; Arna B Agustsdottir; Arnaldur Gylfason; Asgeir Sigurdsson; Aslaug Jonasdottir; Berglind Eiriksdottir; Bjarni Thorbjornsson; Brynjar O Jensson; Daniel F Gudbjartsson; Droplaug N Magnusdottir; Elisabet E Gardarsdottir; Emil A Thorarensen; Gardar Sveinbjornsson; Gisli Masson; Gudmundur Georgsson; Gudmundur L Norddahl; Gudrun Sigmundsdottir; Hakon Jonsson; Hilma Holm; Ingileif Jonsdottir; Jona Saemundsdottir; Kamilla S Josefsdottir; Kari Stefansson; Karl G Kristinsson; Kjartan R Gudmundsson; Kristin E Sveinsdottir; Louise le Roux; Maney Sveinsdottir; Olafia S Gretarsdottir; Olafur T Magnusson; Pall Melsted; Patrick Sulem; Run Fridriksdottir; Thora R Gunnarsdottir; Thordur Kristjansson; Thorolfur Gudnason; Unnur Thorsteinsdottir
EPI_ISL_509028	genXone SA, Molecular Diagnostics Laboratory / NZOZ	genXone SA, Research & Development Laboratory	Grzegorz Nowicki; Jakub Grabowski; Maciej Sykulski; Michał Kaszuba; Monika Mańkowska-Woźniak; Natalia Drwęska-Matelska; Łukasz Krych
EPI_ISL_454574	nstitute for Public Health	Laboratory for advanced genomics	Filip Rokić; Igor Jurak; Lovro Trgovec-Greif; Neven Sučić; Oliver Vugrek; Tomislav Rukavina
EPI_ISL_447636, EPI_ISL_447637, EPI_ISL_447638, EPI_ISL_447639, EPI_ISL_447640, EPI_ISL_447641, EPI_ISL_447642, EPI_ISL_447643, EPI_ISL_447644, EPI_ISL_447645, EPI_ISL_447646, EPI_ISL_447647, EPI_ISL_447648, EPI_ISL_447649, EPI_ISL_447650, EPI_ISL_447651, EPI_ISL_447652, EPI_ISL_447653, EPI_ISL_447833, EPI_ISL_447835			
see above	unknown	Department of Medicine	Bampali, M.; Dovrolis, N.; Froukala, E.; Gatzidou, E.; Kassela, K.; N. and Karakasiliotis, I.; Spanakis; Stavropoulou, A.; Tsakris, A.; Veletza, S.
EPI_ISL_462434, EPI_ISL_462436, EPI_ISL_462437	unknown	Laboratory Diagnostic	Afonso; Banovic Djeri, B.; C.L.; Dmitric, M.; Jankovic, M.; Knezevic, A.; Petrovic, T.; Sekler, M.; Tesovic, B.; Vidanovic, D.; Volkening, J.

We gratefully acknowledge the following Authors from the Originating laboratories responsible for obtaining the specimens, as well as the Submitting laboratories where the genome data were generated and shared via GISAID, on which this research is based.

All Submitters of data may be contacted directly via www.gisaid.org

Authors are sorted alphabetically.

Accession ID	Originating Laboratory	Submitting Laboratory	Authors
EPI_ISL_768758, EPI_ISL_768759	AIID	Irish Coronavirus Sequencing Consortium - National Virus Reference Laboratory	Alejandro Abner Garcia Leon; Gabriel Gonzalez; Michael Carr; Patrick Mallon
EPI_ISL_806474	Alberta Precision Labs (APL)	Alberta Precision Labs (APL)	Berenger B; Bernier F; Chui L; Croxen M; Gordon P; Kellner J; Lam LG; Li V; Ma R; Melin A; Pabbaraju K; Tipples G; Wong A; Zelyas N
EPI_ISL_816860	Bioinformatics and Biostatistics Lab, Advanced Sequencing Facility	COVID-19 Genomics UK (COG-UK) Consortium	Aengus Stewart; Chelsea Sawyer; Harshil Patel; Jerome Nicod; Laura Cubitt; Margaret Crawford
EPI_ISL_644270, EPI_ISL_644277, EPI_ISL_644319, EPI_ISL_644333	CEPHR / Vincent's Hospital	Irish Coronavirus Sequencing Consortium - National Virus Reference Laboratory	Alejandro Abner Garcia Leon; Gabriel Gonzalez; Michael Carr; Patrick Mallon
EPI_ISL_663278	CHU Nantes	CNR Virus des Infections Respiratoires - France SUD	Antonin Bal; Bruno Lina; Celine Bressollette; Gregory Destras; Gwendolyne Burfin; Hadrien Règue; Laurence Josset; Louise Castain; Martine Valette; Quentin Semanas; Virginie Ferré
EPI_ISL_692737	CNR Virus des Infections Respiratoires - France SUD	CNR Virus des Infections Respiratoires - France SUD	Antonin Bal; Bruno Lina; Gregory Destras; Gwendolyne Burfin; Laurence Josset; Martine Valette; Solenne Brun
EPI_ISL_583569, EPI_ISL_583714	Center for Virology, Medical University of Vienna	Bergthaler laboratory, CeMM Research Center for Molecular Medicine of the Austrian Academy of Sciences	Adi Steinrigl; Alexander Lercher; Alexandra Popa; Andreas Bergthaler; Benedikt Agerer; Christian Paar; Christoph Bock; Daniela Schmid; Dorothee von Laer; Elisabeth Puchhammer-Stoeckl; Franz Allerberger; Gernot Walder; Gregor Hörmann; Guenter Weiss; Gunther Vogl; Henrique Colaco; Jakob-Wendelin Genger; Jan Laine; Judith Aberle; Kinga Rigler-Hohenwarter; Lukas Endler; Manfred Nairz; Mark Smyth; Martin Senekowitsch; Michael Schuster; Peter Hufnagl; Peter Obrist; Rainer Gattringer; Sabine Sussitz-Rack; Stephan Aberle; Thomas Penz; Wegene Borena
EPI_ISL_639739	Centre of Nanotechnologies, INCD IMT-Bucuresti (National Institute for Research and Development in Microtechnologies - Bucharest)	Centre of Nanotechnologies, INCD IMT-Bucuresti (National Institute for Research and Development in Microtechnologies - Bucharest)	Gogianu; L. and Baisan, M.; Salceanu, A.
EPI_ISL_732812	Centro de Investigación Biomédica de La Rioja - Hospital San Pedro Logroño	SeqCOVID-SPAIN consortium/IBV(CSIC)	José Manuel Azcona Gutiérrez; María Pilar Bea Escudero; María de Toro; Miriam Blasco Alberdi and SeqCOVID-SPAIN consortium
EPI_ISL_677716, EPI_ISL_677724	Clinical Hospital - Shtip	Research Center for Genetic Engineering and Biotechnology "Georgi D. Efremov" , Macedonian Academy of Sciences and Arts	RCGEB - MASA
EPI_ISL_581622, EPI_ISL_626248	Department of Clinical Microbiology	GIGA Medical Genomics	Bouchra Boujemla; Cécile Meex; Keith Durkin; Maria Artesi; Marie-Pierre Hayette; Pierrette Melin; Raphaël Boreux; Sébastien Bontems; Vincent Bours
EPI_ISL_605793	Department of Experimental Modeling and Pathogenesis of Infectious Diseases	WHO National Influenza Centre Russian Federation	Alekseev A.Yu.; Andrey Komissarov; Anna Ivanova; Artem Fadeev; Chepurnov A.A.; Kononova Yu.V.; Kseniya Komissarova; Shestopalov A.M.; Sobolev I.A.
EPI_ISL_803882	Department of Medical Biotechnologies, University of Siena	Laboratory of Infectious Diseases, Department of Biomedical and Clinical Sciences L. Sacco, University of Milan	Alessia Lai; Annalisa Bergna; Carla Della Ventura; Claudia Balotta; Filippo Dragoni; Gianguglielmo Zehender on behalf of SARS-CoV-2 ITALIAN RESEARCH ENTERPRISE-(SCIRE) Collaborative Group; Ilaria Vicenti; Maria Grazia Cusi; Massimo Galli; Maurizio Zazzi
EPI_ISL_635136, EPI_ISL_708106	Department of Medical Microbiology, St. Olavs hospital	Norwegian Institute of Public Health, Department of Virology	Hilde Elshaug; Hilde Vollan; Kamilla Heddeland Instefjord; Karoline Bragstad; Kathrine Stene-Johansen; Marie Paulsen Madsen; Olav Hungnes; Rasmus Riis Kopperud
EPI_ISL_812968	Department of Molecular Medicine, University of Padova	Department of Molecular Medicine, University of Padova	Abate, D.; Barzon, L.; Besutti, V.; De Canale, E.; Del Vecchio, C.; Franchin, E.; Lavezzo, E.; Loregian, A.; M.C.; Manganelli, R.; Manuto, L.; Masi, G.; Onelia, F.; Pacenti, M.; Parisi; Rossi, L.; S. and Crisanti, A.; S.G.; Saluzzo, F.; Sciro, M.; Toppo; Trevisan, M.; Vanuzzo
EPI_ISL_614561, EPI_ISL_614652, EPI_ISL_614787, EPI_ISL_614841, EPI_ISL_614856, EPI_ISL_617972, EPI_ISL_619983, EPI_ISL_622510			
see above	Department of Virus and Microbiological Special Diagnostics, Statens Serum Institut, Denmark	Albertsen lab, Department of Chemistry and Bioscience, Aalborg University, Denmark	Danish Covid-19 Genome Consortia
EPI_ISL_708044	Dept. of Medical Microbiology, Stavanger University Hospital, Helse Stavanger HF	Norwegian Institute of Public Health, Department of Virology	Hilde Elshaug; Hilde Vollan; Kamilla Heddeland Instefjord; Karoline Bragstad; Kathrine Stene-Johansen; Marie Paulsen Madsen; Olav Hungnes; Rasmus Riis Kopperud
EPI_ISL_591328, EPI_ISL_591335	Dipartimento di Biotecnologie Mediche, University of Siena	Dipartimento di Biotecnologie Mediche, University of Siena	Anichini, G.; Cusi; Gandolfo, C.; M.G.; Pinzauti, D.; Pozzi, G.; Santoro, F.
EPI_ISL_583731, EPI_ISL_583744, EPI_ISL_583782, EPI_ISL_583786, EPI_ISL_583797, EPI_ISL_583818, EPI_ISL_583852			
see above	Dr. Gernot Walder GmbH	Bergthaler laboratory, CeMM Research Center for Molecular Medicine of the Austrian Academy of Sciences	Adi Steinrigl; Alexander Lercher; Alexandra Popa; Andreas Bergthaler; Benedikt Agerer; Christian Paar; Christoph Bock; Daniela Schmid; Dorothee von Laer; Elisabeth Puchhammer-Stoeckl; Franz Allerberger; Gernot Walder; Gregor Hörmann; Guenter Weiss; Gunther Vogl; Henrique Colaco; Jakob-Wendelin Genger; Jan Laine; Judith Aberle; Kinga Rigler-Hohenwarter; Lukas Endler; Manfred Nairz; Mark Smyth; Martin Senekowitsch; Michael Schuster; Peter Hufnagl; Peter Obrist; Rainer Gattringer; Sabine Sussitz-Rack; Stephan Aberle; Thomas Penz; Wegene Borena
EPI_ISL_763101, EPI_ISL_763102, EPI_ISL_763103, EPI_ISL_763110, EPI_ISL_763112, EPI_ISL_763116, EPI_ISL_763118, EPI_ISL_763119			
see above	Dutch COVID-19 response team	Erasmus Medical Center	Anne van der Linden; Annemiek van der Eijk; Bas Oude Munnink; Corine GeurtsvanKessel; David Nieuwenhuijse; Emmanuelle Munger; Irina Chestakova; Marion Koopmans; Marjan Boter; Reina Sikkema; Richard Molenkamp; on behalf of the Dutch national COVID-19 response team.
EPI_ISL_735263, EPI_ISL_735281, EPI_ISL_735284, EPI_ISL_735285, EPI_ISL_735288, EPI_ISL_735324, EPI_ISL_735347			
see above	Genomic Laboratory (GLAB) (Conjoint lab of Health Directorate of Istanbul and Istanbul Technical University)	Genomic Laboratory (GLAB), Istanbul Technical University	Arzu Irvem; Ayse Serra Ozel; Betsi Kose; Gizem Alkurt; Gizem Dinler Doganay; Ilker Karacan; Jale Yildiz; Levent Doganay; Mehtap Aydin; Nihat Bugra Agaoglu; Nilsun Altunal; Nisan Denizce Can; Ozlem Akgun Dogan; Payam Zolfagharian; Tugba Kizilboga Akgun; Yasemin Kendir Demirkol
EPI_ISL_602336, EPI_ISL_602337, EPI_ISL_602340, EPI_ISL_602343, EPI_ISL_602367, EPI_ISL_602450, EPI_ISL_733062, EPI_ISL_733074, EPI_ISL_733096, EPI_ISL_733151			
see above	HELIX LLC	WHO National Influenza Centre Russian Federation	Andrey Komissarov; Anna Ivanova; Artem Fadeev; Daria Danilenko; Dmitry Bazhenov; Dmitry Lioznov; Elena Nabieva; Georgii Bazykin; Ksenia Safina; Kseniya Komissarova
EPI_ISL_654294, EPI_ISL_780070, EPI_ISL_780074	Hospital General Universitario Gregorio Marañón	SeqCOVID-SPAIN consortium/IBV(CSIC)	Darío García de Viedma; Jon Sicilia; Julia Suárez; Laura Pérez-Lago; Marta Herranz; Patricia Muñoz and SeqCOVID-SPAIN consortium; Pilar Catalán
EPI_ISL_819297, EPI_ISL_819302, EPI_ISL_819303, EPI_ISL_819304, EPI_ISL_819305, EPI_ISL_819306, EPI_ISL_819310, EPI_ISL_819312, EPI_ISL_819313, EPI_ISL_819315, EPI_ISL_819316, EPI_ISL_819317, EPI_ISL_819318, EPI_ISL_819319, EPI_ISL_819324, EPI_ISL_819350, EPI_ISL_819355, EPI_ISL_819356, EPI_ISL_819359, EPI_ISL_819360, EPI_ISL_824418, EPI_ISL_824445, EPI_ISL_824465			
see above	Hospital Universitari Vall d'Hebron - Vall d'Hebron Institut de Recerca	Hospital Universitari Vall d'Hebron - Vall d'Hebron Institut de Recerca	Andrés Antón; Ariadna Rando; Carla Castillo; Cristina Andrés; Damir Garcia-Cehic; Josep F Abril; Josep Quer; Juliana Esperalba; Maria Carmen Martin; Maria Gema Codina; Maria Piñana; Tomàs Pumarola
EPI_ISL_833197	Hôpital Bichat Claude Bernard, Laboratoire de Virologie	IAME UMR1137 Inserm, Université de Paris, Hôpital Bichat	Alexandre Storto; Amélie Recoing; Antoine Bridier; Benoit Visseaux; Charlotte Charpentier; Diane Descamps; Gilles Collin; Lena Daniel; Mélanie Bertine; Nadhira Houhou-Fidouh; Quentin Le Hingrat; Siham Hamri
EPI_ISL_649785, EPI_ISL_653771, EPI_ISL_653773, EPI_ISL_653801, EPI_ISL_653806	I.R.C.C.S. "S. De Bellis" - Ente Ospedaliero	Istituto Zooprofilattico Sperimentale della Puglia e della Basilicata	Bianco A.; Capozzi L.; Cipolletta D.; Del Sambro L.; Galante D.; Lippolis A.; Manzulli V; Notarnicola M.; Pace L.; Parisi A.; Rondinone V.
EPI_ISL_603178, EPI_ISL_609995	INMI Lazzaro Spallanzani IRCCS	INMI Lazzaro Spallanzani IRCCS	A Di Caro; Antonino Di Caro; B Bartolini; Barbara Bartolini; C.E.M Gruber; Cesare E.M. Gruber; E Giombini; Emanuela Giombini; F Messina; Francesco Messina; M Rueca; MR Capobianchi; Maria R. Capobianchi; Martina Rueca; Simone Lanini
EPI_ISL_751400, EPI_ISL_751426	IRCCS Sacro Cuore Don Calabria Hospital, Department of Infectious, Tropical Diseases & Microbiology	University of Verona, Department of Biotechnology	Antonio Mori; Chiara Degli Esposti; Chiara Piubelli; Cristina Beltrami; Elena Pomari; Emanuela Cosentino; Giulia Lopatriello; Luca Marcolungo; Massimo Delledonne; Michela Deiana
EPI_ISL_738122, EPI_ISL_738129, EPI_ISL_738131, EPI_ISL_738132	IZSM-U.O.C. Virologia	Istituto Zooprofilattico Sperimentale del Mezzogiorno	Giovanna Fusco; Lorena Cardillo; Maurizio Viscardi
EPI_ISL_583614	Institut für Virologie am Department für Hygiene, Mikrobiologie und Public Health	Bergthaler laboratory, CeMM Research Center for Molecular Medicine of the Austrian Academy of Sciences	Adi Steinrigl; Alexander Lercher; Alexandra Popa; Andreas Bergthaler; Benedikt Agerer; Christian Paar; Christoph Bock; Daniela Schmid; Dorothee von Laer; Elisabeth Puchhammer-Stoeckl; Franz Allerberger; Gernot Walder; Gregor Hörmann; Guenter Weiss; Gunther Vogl; Henrique Colaco; Jakob-Wendelin Genger; Jan Laine; Judith Aberle; Kinga Rigler-Hohenwarter; Lukas Endler; Manfred Nairz; Mark Smyth; Martin Senekowitsch; Michael Schuster; Peter Hufnagl; Peter Obrist; Rainer Gattringer; Sabine Sussitz-Rack; Stephan Aberle; Thomas Penz; Wegene Borena
EPI_ISL_574804, EPI_ISL_574812, EPI_ISL_574815, EPI_ISL_574816, EPI_ISL_574822, EPI_ISL_574830, EPI_ISL_574834, EPI_ISL_574853			

see above	Institute for Infectious Diseases, University of Bern	Institute for Infectious Diseases, University of Bern	Alban Ramette; Christian Baumann; Cora Sägesser; Franziska Suter-Riniker; Michel C Koch; Miguel A Terrazos Miani; Peter Keller; Stephen L Leib
EPI_ISL_602464, EPI_ISL_602465, EPI_ISL_602476, EPI_ISL_602488, EPI_ISL_602489, EPI_ISL_602500, EPI_ISL_626230, EPI_ISL_717977			
see above	Institute for Virology, University Hospital Essen	Center of Medical Microbiology, Virology, and Hospital Hygiene, University of Duesseldorf	Alexander Dilthey; Andreas Walker; Daniel Strelow; Jessica Nicolai; Jörg Timm; Klaus Pfeffer; Lisanna Hülse; Malte Kohns Vasconcelos; Maximilian Damagnez; Nadine Lübke; Olympia E. Anastasiou; Tobias Wienemann; Torsten Houwaart; Ulf Dittmer
EPI_ISL_635209, EPI_ISL_635210, EPI_ISL_635212, EPI_ISL_635213, EPI_ISL_635214, EPI_ISL_635215, EPI_ISL_635216, EPI_ISL_635218, EPI_ISL_635219, EPI_ISL_635220, EPI_ISL_635221, EPI_ISL_635223, EPI_ISL_635224, EPI_ISL_635227, EPI_ISL_635229, EPI_ISL_635237, EPI_ISL_635253, EPI_ISL_635257, EPI_ISL_635262, EPI_ISL_635286			
see above	Institute of Microbiology and Immunology, Faculty of Medicine, University of Ljubljana	Institute of Microbiology and Immunology, Faculty of Medicine, University of Ljubljana	Mario Poljak; Miša Korva; Samo Zakotnik; Tatjana Avšič - Županc; Tatjana Avšič – Županc; Tomaž Mark Zorec
EPI_ISL_583481, EPI_ISL_583486, EPI_ISL_583488, EPI_ISL_718258, EPI_ISL_718259, EPI_ISL_788980	Institute of Virology, Biomedical Research Center of the Slovak Academy of Sciences, Bratislava	Faculty of Natural Sciences, Comenius University, Bratislava	Boris Klempa; Broňa Brejová; Dominika Fričová; Edita Staroňová; Elena Tichá; Jozef Nosek; Juraj Kopáček; Kristína Boršová; Martina Ličková; Martina Neboháčová; Monika Sláviková; Sabina Fumačová Havlíková; Tomáš Vinař; Viktória Hodorová; Viktória Čabanová; Ľubomíra Lukáčiková
EPI_ISL_824408	Istituto Nazionale Malattie Infettive Lazzaro Spallanzani IRCCS	Istituto Nazionale Malattie Infettive Lazzaro Spallanzani IRCCS	Antonino Di Caro; Barbara Bartolini; Cesare E.M. Gruber; Concetta Castilletti; Emanuela Giombini; Francesca Colavita; Francesco Messina; Maria R. Capobianchi; Martina Rueca; Ornella Butera; Silvia Meschi
EPI_ISL_736835, EPI_ISL_776879, EPI_ISL_833537, EPI_ISL_833547, EPI_ISL_833551, EPI_ISL_833555, EPI_ISL_833561, EPI_ISL_837483			
see above	Istituto Zooprofilattico Sperimentale del Mezzogiorno	TIGEM	Andrea Ballabio; Anna Manfredi; Antonio Grimaldi; Antonio Limone; Biancamaria Pierri; Chiara Colantuono; Davide Cacchiarelli.; Denise Di Concilio; Francesco Panariello; Lucio Di Filippo; Marcello Salvi; Maria Concetta Cuomo; Patrizia Annunziata; Pellegrino Cerino; Valentina Bouche
EPI_ISL_763095, EPI_ISL_763330	Istituto Zooprofilattico Sperimentale dell' Umbria e delle Marche -Togo Rosati	Istituto Superiore di Sanità	Gabriele Vaccari; Giovanni Ianiro; Ilaria Di Bartolo; Luca De Sabato; Massimo Biagetti; Monica Giammarioli
EPI_ISL_722877, EPI_ISL_722878, EPI_ISL_722886, EPI_ISL_722889, EPI_ISL_722891, EPI_ISL_722904, EPI_ISL_722907, EPI_ISL_722913, EPI_ISL_722915, EPI_ISL_722924, EPI_ISL_794752, EPI_ISL_794792, EPI_ISL_794808			
see above	Istituto Zooprofilattico Sperimentale della Puglia e della Basilicata	Istituto Zooprofilattico Sperimentale della Puglia e della Basilicata	Bianco A.; Capozzi L.; Cipolletta D.; Del Sambro L.; Galante D.; Manzulli V; Pace L.; Parisi A.; Rondinone V.
EPI_ISL_728292, EPI_ISL_763086, EPI_ISL_763087, EPI_ISL_763089, EPI_ISL_763090, EPI_ISL_763091, EPI_ISL_763092			
see above	Jena University Hospital, Institute for Infectious Diseases and Infection Control	Institute of infectious medicine & hospital hygiene, CaSe-Group	Brandt; Christian; Marquet; Matthias W.; Mike; Pletz; Riccardo; Spott
EPI_ISL_739733, EPI_ISL_739883, EPI_ISL_739934, EPI_ISL_740062, EPI_ISL_744246, EPI_ISL_744496, EPI_ISL_744688			
see above	Laboratoire national de santé, Microbiology, Virology	Laboratoire national de santé, Microbiology, Microbial Genomics Platform	Anke Wienecke-Baldacchino; Catherine Ragimbeau; Fatu Djabi; Jessica Tapp; Lise Pignon; Raoul Salmon; Tamir Abdelrahman
EPI_ISL_613706, EPI_ISL_613955, EPI_ISL_614396, EPI_ISL_614398, EPI_ISL_614889, EPI_ISL_637108, EPI_ISL_637109			
see above	Laboratorio Biologia Molecolare Sars Cov2 - UOC Laboratorio Analisi - Servizio Medicina di Laboratorio, Ospedale "San Francesco" - ATS-ASSL Nuoro	Laboratorio specialistico UOC Ematologia - Ospedale "San Francesco" - ATS-ASSL Nuoro	Aspronì Rosanna; Casu Gavino; Fancello Tatiana; Fiamma Maura; Floris Anna Rita; Lo Maglio Iana; Mameli Giuseppe; Monne Maria Itria; Palmas Angelo Domenico; Piras Giovanna; Sanna Filomena; Sulis Vincenzo; Toja Alessandro
EPI_ISL_710503	Laboratorio specialistico UOC Ematologia - Ospedale "San Francesco" - ATS-ASSL Nuoro	Laboratorio specialistico UOC Ematologia - Ospedale "San Francesco" - ATS-ASSL Nuoro	Giovanna Piras
EPI_ISL_754911, EPI_ISL_754913	Laboratory Diagnostics and Clinical Immunology of Developmental Age, Medical University of Warsaw	genXone SA, Research & Development Laboratory; The Faculty of Mathematics, Informatics and Mechanics of the University of Warsaw	Anna Gambin; Grzegorz Nowicki; Jakub Grabowski; Maciej Sykulski; Michał Kaszuba; Monika Mańkowska-Woźniak; Natalia Drwęska-Matelska; Urszula Demkow; Łukasz Krych
EPI_ISL_779704, EPI_ISL_779709, EPI_ISL_779712, EPI_ISL_779713, EPI_ISL_779714	Laboratory of Infectious Diseases, Department of Biomedical and Clinical Sciences L. Sacco, University of Milan	Laboratory of Infectious Diseases, Department of Biomedical and Clinical Sciences L. Sacco, University of Milan	Alessia Lai; Annalisa Bergna; Carla Della Ventura; Claudia Balotta; Gianguglielmo Zehender on behalf of SARS-CoV-2 ITALIAN RESEARCH ENTERPRISE-(SCIRE) Collaborative Group; Massimo Galli
EPI_ISL_701861, EPI_ISL_703185, EPI_ISL_708919, EPI_ISL_721477, EPI_ISL_761806, EPI_ISL_767135, EPI_ISL_826506			
see above	Lighthouse Lab in Alderley Park	Wellcome Sanger Institute for the COVID-19 Genomics UK (COG-UK) Consortium	Cordelia Langford; David K. Jackson; Dominic Kwiatkowski; Ewan Harrison; Ian Johnston; Jacquelyn Wynn; John Sillitoe on behalf of the Wellcome Sanger Institute COVID-19 Surveillance Team; Mairead Hyland; Roberto Amato; Sonia Goncalves; The Lighthouse Lab in Alderley Park and Alex Alderton
EPI_ISL_589391, EPI_ISL_607209	Lighthouse Lab in Alderley Park	Wellcome Sanger Institute for the COVID-19 Genomics UK (COG-UK) consortium	Cordelia Langford; David K. Jackson; Dominic Kwiatkowski; Ewan Harrison; Ian Johnston; Jacquelyn Wynn; John Sillitoe on behalf of the Wellcome Sanger Institute COVID-19 Surveillance Team; Mairead Hyland; Roberto Amato; Sonia Goncalves; The Lighthouse Lab in Alderley Park and Alex Alderton
EPI_ISL_702070, EPI_ISL_783390	Lighthouse Lab in Cambridge	Wellcome Sanger Institute for the COVID-19 Genomics UK (COG-UK) Consortium	Cordelia Langford; David K. Jackson; Dominic Kwiatkowski; Ewan Harrison; Ian Johnston; John Sillitoe on behalf of the Wellcome Sanger Institute COVID-19 Surveillance Team; Rob Howes; Roberto Amato; Sonia Goncalves; The Lighthouse Lab in Cambridge and Alex Alderton
EPI_ISL_597021	Lighthouse Lab in Cambridge	Wellcome Sanger Institute for the COVID-19 Genomics UK (COG-UK) consortium	Cordelia Langford; David K. Jackson; Dominic Kwiatkowski; Ewan Harrison; Ian Johnston; John Sillitoe on behalf of the Wellcome Sanger Institute COVID-19 Surveillance Team (http://www.sanger.ac.uk/covid-team); Rob Howes; Roberto Amato; Sonia Goncalves; The Lighthouse Lab in Cambridge and Alex Alderton
EPI_ISL_731353, EPI_ISL_733954, EPI_ISL_760514, EPI_ISL_762055	Lighthouse Lab in Glasgow	Wellcome Sanger Institute for the COVID-19 Genomics UK (COG-UK) Consortium	Anna Dominiczak and Alex Alderton; Carol Clugston; Cordelia Langford; David Gray; David K. Jackson; Dominic Kwiatkowski; Ewan Harrison; Harper VanSteenhouse; Ian Johnston; John Sillitoe on behalf of the Wellcome Sanger Institute COVID-19 Surveillance Team; Roberto Amato; Sonia Goncalves; Yumi Kasai
EPI_ISL_590443, EPI_ISL_597272	Lighthouse Lab in Glasgow	Wellcome Sanger Institute for the COVID-19 Genomics UK (COG-UK) consortium	Anna Dominiczak and Alex Alderton; Carol Clugston; Cordelia Langford; David Gray; David K. Jackson; Dominic Kwiatkowski; Ewan Harrison; Harper VanSteenhouse; Ian Johnston; John Sillitoe on behalf of the Wellcome Sanger Institute COVID-19 Surveillance Team (http://www.sanger.ac.uk/covid-team); Roberto Amato; Sonia Goncalves; Yumi Kasai
EPI_ISL_673965, EPI_ISL_709609, EPI_ISL_709689, EPI_ISL_720556, EPI_ISL_735718, EPI_ISL_821323	Lighthouse Lab in Milton Keynes	Wellcome Sanger Institute for the COVID-19 Genomics UK (COG-UK) Consortium	Cordelia Langford; David K. Jackson; Dominic Kwiatkowski; Ewan Harrison; Ian Johnston; John Sillitoe on behalf of the Wellcome Sanger Institute COVID-19 Surveillance Team; Roberto Amato; Sonia Goncalves; The Lighthouse Lab in Milton Keynes and Alex Alderton
EPI_ISL_598522, EPI_ISL_598611, EPI_ISL_606722, EPI_ISL_630033, EPI_ISL_634271	Lighthouse Lab in Milton Keynes	Wellcome Sanger Institute for the COVID-19 Genomics UK (COG-UK) consortium	Cordelia Langford; David K. Jackson; Dominic Kwiatkowski; Ewan Harrison; Ian Johnston; John Sillitoe on behalf of the Wellcome Sanger Institute COVID-19 Surveillance Team; John Sillitoe on behalf of the Wellcome Sanger Institute COVID-19 Surveillance Team (http://www.sanger.ac.uk/covid-team); Roberto Amato; Sonia Goncalves; The Lighthouse Lab in Milton Keynes and Alex Alderton
EPI_ISL_636846, EPI_ISL_770511, EPI_ISL_770515, EPI_ISL_770521, EPI_ISL_770531	Lithuanian University of Health Sciences Hospital, Department of Laboratory Medicine	Lithuanian University of Health Sciences, Molecular cardiology lab.	Arnoldas Pautienius; Astra Vitkauskiene; Dovydas Gecys; Ingrida Olendraitė; Kamile Tamusauskaite; Laura Pareckaite; Lukas Zemaitis; Vaiva Lesauskaite
EPI_ISL_612411	Liverpool Clinical Laboratories	COVID-19 Genomics UK (COG-UK) Consortium	A Alrezaihi; Alessandro Gerada; Alistair Darby; Angela Cowell; Anita Lucaci; Anu Chawla; Cassie Olateju; Catherine Hartley; Charlotte Nelson; Ecaterina Vamos; Elaine O'Toole; Eleanor G Bentley; Ghada T Shawli; Isabel García-Dorival; James Johnson; James P Stewart; Jenifer Manson; Joanne Watts; Jones Benjamin; Jordan J Clark; Julian Hiscox; L Luu; Lucille Rainbow; M Almsaud; Margaret Hughes; Mark Whitehead; Matthew Gemmell; Miren Iturriza-Gomara; Muhannad Alruwaili; N.P Randle; Neil Swainston; PKF Gilmore; Parul Sharma; Rebekah Penrice-Randal; Richard Eccles; Richard Gregory; Sam Haldenby; Steve Paterson; Stuart D Armstrong; Trevor Ian Robinson; Ximeng Han
EPI_ISL_644433	MEPHI, Aix Marseille University	MEPHI, Aix Marseille University	Anthony LEVASSEUR
EPI_ISL_668400	Medical Microbiology Unit, Department for Laboratory Medicine, Drammen Hospital, Vestre Viken Health Trust,	Norwegian Institute of Public Health, Department of Virology	Hilde Elshaug; Hilde Vollan; Kamilla Heddeland Instefjord; Karoline Bragstad; Kathrine Stene-Johansen; Marie Paulsen Madsen; Olav Hungnes; Rasmus Riis Kopperud
EPI_ISL_738147, EPI_ISL_738194, EPI_ISL_738243	Microbiology and Virology Unit, Florence Careggi University Hospital	Microbiology and Virology Unit, Florence Careggi University Hospital	Alberto Antonelli; Gian Maria Rossolini; Marco Coppi; Simona Pollini; Vincenzo Di Pilato
EPI_ISL_613637, EPI_ISL_613958, EPI_ISL_632818, EPI_ISL_632821, EPI_ISL_637196, EPI_ISL_637219, EPI_ISL_671271, EPI_ISL_671296, EPI_ISL_671297, EPI_ISL_671300, EPI_ISL_671321			
see above	Microbiology, Department of Pathology, St. Bernard's Hospital, Gibraltar Health Authority	Respiratory Virus Unit, Microbiology Services Colindale, Public Health England	Charlotte Gillborn-Jones (Gibraltar); Dr Nicholas Cortes (Gibraltar); PHE Covid Sequencing Team
EPI_ISL_709983	Microbiology, Department of Pathology, St. Bernard's Hospital, Gibraltar Health Authority	Respiratory Virus Unit, National Infection Service, Public Health England	Charlotte Gillborn-Jones (Gibraltar); Dr Nicholas Cortes (Gibraltar); PHE Covid Sequencing Team
EPI_ISL_632312	NU-sjukvården	Clinical microbiology, Sahlgrenska	Hedvig Engström Jakobsson; Johan Ringlander; Josefin Olausson; Magnus Lindh

		University Hospital	
EPI_ISL_718262, EPI_ISL_746835	National Institute for Infectious Diseases, INMI, "L. Spallanzani" IRCCS	National Institute for Infectious Diseases, INMI, "L. Spallanzani" IRCCS	A Di Caro; B Bartolini; C.E.M Gruber; E Giombini; F Messina; M Rueca; MR Capobianchi
EPI_ISL_666697, EPI_ISL_666701, EPI_ISL_666709	National Virus Reference Lab (NVRL)	Irish Coronavirus Sequencing Consortium- Teagasc Grange	Aljandro Abner Garcia Leon; Calum Walsh; Fiona Crispie; Matthew McCabe; Michael Carr; Paul Cotter
EPI_ISL_848155	National Virus Reference Laboratory	Irish Coronavirus Sequencing Consortium - National University of Ireland Galway	Grainne Mc Andrew; Kate Reddington; Simone Coughlan
EPI_ISL_671378, EPI_ISL_671402, EPI_ISL_681899, EPI_ISL_681911, EPI_ISL_681918, EPI_ISL_681927, EPI_ISL_767736, EPI_ISL_767751, EPI_ISL_767768, EPI_ISL_767815, EPI_ISL_767830, EPI_ISL_767831, EPI_ISL_767833, EPI_ISL_791313			
see above	National Virus Reference Laboratory	Irish Coronavirus Sequencing Consortium - Teagasc Moorepark	Alejandro Abner Garcia Leon; Calum Walsh; Fiona Crispie; Genuity Ireland; John Kenny; Matthew McCabe; Paddy Mallon; Paul Cotter
EPI_ISL_639940	Omsk Research Institute of Natural Focal Infections	WHO National Influenza Centre Russian Federation	Aleksei Vasilenko; Andrey Komissarov; Artem Fadeev; Daria Nashatyreva; Ekaterina Gradoboeva; Ekaterina Savkina; Elena Poleshchuk; Valery Yakimenko
EPI_ISL_841369	Originating lab: Wales Specialist Virology Centre Sequencing lab: Pathogen Genomics Unit	Public Health Wales Microbiology Cardiff Wales Specialist Virology Centre	Alec Birchley; Alexander Adams; Amy Gaskin; Angela Marchbank; Bree Gatica-Wilcox; Catherine Moore; Jason Coombes; Joanne Watkins; Joel Southgate; Johnathan Evans; Laura Gifford; Lauren Gilbert; Lee Graham; Malorie Perry; Matthew Bull; Nicole Pacchiarini; Sally Corden; Sara Kumziene-Summerhayes; Sara Rey; Sarah Taylor; Simon Cottrell; Sophie Jones; Tom Connor
EPI_ISL_590914, EPI_ISL_668401	Oslo University Hospital, Department of Medical Microbiology	Norwegian Institute of Public Health, Department of Virology	Hilde Elshaug; Hilde Vollan; Kamilla Heddeland Instefjord; Karoline Bragstad; Kathrine Stene-Johansen; Marie Paulsen Madsen; Olav Hungnes; Rasmus Riis Kopperud
EPI_ISL_788962	Ospedale "Di Venere"	Beaconlab (Bioinformatics, Evolution and Comparative Genomics lab), Dept of Biosciences, University on Milan	Chiara M; Iacobellis M; Manzari C; Parisi A; Pesole G; Piluscio R; d'Avenia M
EPI_ISL_747467	Ospedale Mater Salutis	Istituto Zooprofilattico Sperimentale delle Venezie	Adelaide Milani; Alessia Schivo; Alice Fusaro; Ambra Pastori; Annalisa Salviato; Antonia Ricci; Bianca Zecchin; Calogero Terregino; Erika Giorgia Quaranta; Isabella Monne
EPI_ISL_635116	Ostfold Hospital Trust - Kalnes, Centre for Laboratory Medicine, Section for gene technology and infection serology	Norwegian Institute of Public Health, Department of Virology	Hilde Elshaug; Hilde Vollan; Kamilla Heddeland Instefjord; Karoline Bragstad; Kathrine Stene-Johansen; Marie Paulsen Madsen; Olav Hungnes; Rasmus Riis Kopperud
EPI_ISL_596327, EPI_ISL_733208, EPI_ISL_733220	Pathogenic Microorganisms Variability Laboratory	WHO National Influenza Centre Russian Federation	Alexander Gintsburg; Alexey Shchetinin; Andrey Komissarov; Andrey Pochtovyy; Anna Ignatieva; Anna Ivanova; Artem Fadeev; Artem Tkachuk; Daria Danilenko; Dmitry Bazhenov; Dmitry Lioznov; Ekaterina Milashenko; Elena Burtseva; Elena Nabieva; Elena Shidlovskaya; Elena Vokalova; Elizaveta Divisenko; Evgeniya Mukasheva; Evgeny Usachev; Evgeny Usachev; Georgii Bazykin; Kirill Krasnoslobotsev; Ksenia Safina; Kseniya Komissarova; Ludmila Kolobukhina; Maria Nikiforova; Maxim Rubalsky; Nadezhda Kuznetsova; Oleg Rubalsky; Olga Burgasova; Sergey Alkhovsky; Svetlana Smetanina; Svetlana Trushakova; Tatyana Vishnevskaya; Valeria Bacalin; Vladimir Gushchin
EPI_ISL_595914	Quadram Institute Bioscience	COVID-19 Genomics UK (COG-UK) Consortium	Alexander J Trotter; Alison E. Mather; Alp Aydin; Ana P. Tedim; Anastasia Kolyva; Andrew Bell; Andrew J. Page; Claire Stuart; Dave J. Baker; Gemma L. Kay; John Wain; Justin O'Grady; Leonardo de Oliveira Martins; Lizzie Meadows; Maria Diaz; Mark Webber; Muhammed Yasir; Nabil-Fareed Alikhan; Ngozi Elumogo; Nicholas M. Thomson; Rachael Stanley; Rachel Gilroy; Reenesh Prakash; Samir Dervisevic; Samuel Bloomfield; Steven Rudder; Thanh Le-Viet
EPI_ISL_594972	Queens Medical Centre, Clinical Microbiology Department / DeepSeq Nottingham	COVID-19 Genomics UK (COG-UK) Consortium	Christopher Moore; Fei Sang; Gemma Clark; Hannah Howson-Wells; Johnny Debebe; Jonathan Ball; Joseph Chappell; Manjinder Khakh; Matthew Carlisle; Matthew Loose; Michelle M Lister; Nadine Holmes; Patrick McClure; Theocharis Tsoleridis; Vicki M Fleming; Victoria Wright; Wendy Smith
EPI_ISL_639746, EPI_ISL_639797	Queensland Health Forensic and Scientific Services	Queensland Health Forensic and Scientific Services	Son Nguyen et al
EPI_ISL_585130	Regional Virus Laboratory, Belfast Health and Social Care Trust	COVID-19 Genomics UK (COG-UK) Consortium	Alison Watt; Ciara Cox; Conall McCaughey; David Simpson; Derek Fairley; James McKenna; Mairead Connor; Susan Feeney; Tanya Curran; Zoltan Molnar
EPI_ISL_686587	Respiratory Virus Unit, Microbiology Services Colindale, Public Health England	COVID-19 Genomics UK (COG-UK) Consortium	PHE Covid Sequencing Team
EPI_ISL_661189, EPI_ISL_661200, EPI_ISL_676577, EPI_ISL_676593	Scientific Veterinary Institute Novi Sad	Veterinary Specialized Institute "Kraljevo", Serbia	Afonso, C.; Banovic Djeri, B.; Jankovic, M.; Jovanovic, T.; Knezevic, A.; Petrovic, T.; Sekler, M.; Tesovic, B.; Vidanovic, D.; Volkening, J.
EPI_ISL_654651	Servicio de Microbiología, Hospital Universitario Central de Asturias	SeqCOVID-SPAIN consortium/IBV(CSIC)	Cristián Castelló Abietar; Jose A. Boga; Marta Elena Álvarez-Argüelles; Santiago Melón and SeqCOVID-SPAIN consortium; Susana Rojo-Alba
EPI_ISL_691678, EPI_ISL_779860	Servicio de Microbiología, Hospital Universitario Son Espases	SeqCOVID-SPAIN consortium/IBV(CSIC)	Antonio Oliver and SeqCOVID-SPAIN consortium; Carla López-Causapé; Jordi Reina
EPI_ISL_683612	Servicio de Microbiología, Laboratori Clínic Metropolitana Nord. Hospital Universitari Germans Trias i Pujol. Institut d'Investigació en Ciències de la Salut Germans Trias i Pujol (IGTP)	SeqCOVID-SPAIN consortium/IBV(CSIC)	Adrián Antuori; Anabel Fernández; Anna Not; Antoni E. Bordoy; Cristina Casañ and SeqCOVID-SPAIN consortium; Elisa Martró; Nona Romaní; Verónica Saludes
EPI_ISL_577629, EPI_ISL_577632, EPI_ISL_584073, EPI_ISL_584079, EPI_ISL_626593	The National Institute of Public Health	State Veterinary Institute Prague	A; D; H; J; Jirincova; L; Nagy; Novakova; Trnka; Vecerova
EPI_ISL_827075, EPI_ISL_827413, EPI_ISL_827577, EPI_ISL_828775, EPI_ISL_828776, EPI_ISL_828779, EPI_ISL_828811, EPI_ISL_828940, EPI_ISL_829721			
see above	The National University Hospital of Iceland	deCODE genetics	Agnar Helgason; Alma Moller; Arna B Agustsdottir; Arnaldur Gylfason; Asgeir Sigurdsson; Aslaug Jonasdottir; Berglind Eiriksdottir; Bjarni Thorbjornsson; Brynjar O Jensson; Daniel F Gudbjartsson; Droplaug N Magnusdottir; Elisabet E Gardarsdottir; Emil A Thorarensen; Gardar Sveinbjornsson; Gisli Masson; Gudmundur Georgsson; Gudmundur L Norddahl; Gudrun Sigmundsdottir; Hakon Jonsson; Hannes Eggertsson; Hilma Holm; Ingileif Jonsdottir; Jona Saemundsdottir; Kamilla S Josefsdottir; Kari Stefansson; Karl G Kristinsson; Kjartan R Gudmundsson; Kristin E Sveinsdottir; Louise le Roux; Maney Sveinsdottir; Olafia S Gretarsdottir; Olafur T Magnusson; Pall Melsted; Patrick Sulem; Run Fridriksdottir; Solvi Rognvaldsson; Thora R Gunnarsdottir; Thordur Kristjansson; Thorolfur Gudnason; Unnur Thorsteinsdottir
EPI_ISL_754235	The Republican Research and Practical Center for Epidemiology and Microbiology (RRPCEM)	WHO National Influenza Centre Russian Federation	Anatoly Krasko; Andrey Komissarov; Anna Ivanova; Artem Fadeev; Daria Danilenko; Dmitry Bazhenov; Dmitry Lioznov; Elena Gasich; Elena Nabieva; Georgii Bazykin; Kirill Bulda; Ksenia Safina; Kseniya Komissarova
EPI_ISL_636467, EPI_ISL_636470, EPI_ISL_636474	ULSS6 Distretto Padova Terme Colli	Istituto Zooprofilattico Sperimentale delle Venezie	Adelaide Milani; Alessia Schivo; Alice Fusaro; Ambra Pastori; Annalisa Salviato; Antonia Ricci; Bianca Zecchin; Calogero Terregino; Erika Giorgia Quaranta; Isabella Monne
EPI_ISL_636462	ULSS6 Euganea	Istituto Zooprofilattico Sperimentale delle Venezie	Adelaide Milani; Alessia Schivo; Alice Fusaro; Ambra Pastori; Annalisa Salviato; Antonia Ricci; Bianca Zecchin; Calogero Terregino; Erika Giorgia Quaranta; Isabella Monne
EPI_ISL_733225	UMMC-Health	WHO National Influenza Centre Russian Federation	Andrey Komissarov; Anna Ivanova; Artem Fadeev; Daria Danilenko; Dmitry Bazhenov; Dmitry Lioznov; Elena Nabieva; Georgii Bazykin; Ksenia Safina; Kseniya Komissarova; Tatiana Platonova
EPI_ISL_583953, EPI_ISL_583954, EPI_ISL_583955, EPI_ISL_583958, EPI_ISL_583959, EPI_ISL_583960, EPI_ISL_583962			
see above	UOC Microbiologia e Virologia, Azienda Ospedaliera Universitaria Senese, Siena, Italy	Dipartimento di Biotecnologie Mediche	Claudia Gandolfo; David Pinzauti; Francesco Santoro; Gabriele Anichini; Gianni Pozzi; Maria Grazia Cusi
EPI_ISL_734505, EPI_ISL_734509, EPI_ISL_734510, EPI_ISL_734517, EPI_ISL_734518, EPI_ISL_734544, EPI_ISL_734728, EPI_ISL_734744, EPI_ISL_734827, EPI_ISL_734927, EPI_ISL_734929, EPI_ISL_734933, EPI_ISL_735171, EPI_ISL_738201, EPI_ISL_738204, EPI_ISL_738210, EPI_ISL_738219, EPI_ISL_738242			
see above	UZ Leuven, National Reference Laboratory for Coronaviruses, Laboratory Medicine, Leuven, Belgium	KU Leuven, Rega Institute, Clinical and Epidemiological Virology	Bert Vanmechelen; Joan Marti-Carerras; Piet Maes; Tony Wawina-Bokalanga
EPI_ISL_679436	University College London Hospital	COVID-19 Genomics UK (COG-UK) Consortium	Catherine Houlihan; Dan Frampton; Judith Heaney; Matthew Byott; Moira Spyer and Eleni Nastouli; Stuart Kirk
EPI_ISL_710555, EPI_ISL_710557, EPI_ISL_710572	University Hospital Dubrava	Ruđer Boškovic Institute; Forensic Science Centre Ivan Vučetić; University of Zagreb Faculty of Science	Ana Livun; Antonela Blažeković; Boris Maček; Danilo Licastro; Dunja Glavaš; Fran Borovečki; Fuad Čosović; Gordana Maravić Vlahoviček; Ivan Šamija; Ivana Ćelap; Jasna Kašman; Josipa Skelin; Katarina Marija Tupek; Kristian Vlahoviček; Kristina Gotovac Jerečić; Lidija Cvetko-Krajinović; Lucija Basić; Lucija Markulin; Maja Kuzman; Marina Korolija; Mario Štefanović; Mirjana Domazet-Lošo; Paula Štancl; Petra Vrabec; Robert Belužić; Rosa Karlić; Sanja Tadinac; Senčica Pejša; Tomislav Domazet-Lošo; Valentina Đumlijan-Combaj; Vjekoslav Tomaić; Vladimir Krajinović; Željka Mačak Šafranko
EPI_ISL_681708, EPI_ISL_681716, EPI_ISL_681769, EPI_ISL_681773	University Hospital Limerick	Irish Coronavirus Sequencing Consortium - Teagasc Moorepark	Amy Fitzpatrick; Calum Walsh; Carolyn Meaney; Elaine Lawton; Fiona Crispie; John Kenny; Patrick Stapleton; Paul Cotter
EPI_ISL_847953	University Hospitals of Geneva, Laboratory of Virology	HUG, Laboratory of Virology and the Health2030 Genome Center	Ana Rita Goncalves; Emmanouil Dermitzakis; Henri Pegeot; Ioannis Xenarios; Keith Harshman; Laurent Kaiser; Lorenzo Cerutti; Melyssa Elies; Samuel Cordey
EPI_ISL_775929, EPI_ISL_775943, EPI_ISL_775945, EPI_ISL_775948, EPI_ISL_775949, EPI_ISL_775952, EPI_ISL_775953, EPI_ISL_775957, EPI_ISL_775960, EPI_ISL_775967, EPI_ISL_775968, EPI_ISL_775969, EPI_ISL_775972, EPI_ISL_775973, EPI_ISL_775975, EPI_ISL_775976, EPI_ISL_775985, EPI_ISL_775987, EPI_ISL_776099, EPI_ISL_776202, EPI_ISL_776210, EPI_ISL_776216, EPI_ISL_776217, EPI_ISL_776221, EPI_ISL_776228, EPI_ISL_776286, EPI_ISL_776313, EPI_ISL_776345, EPI_ISL_776352, EPI_ISL_776358, EPI_ISL_776376, EPI_ISL_776394, EPI_ISL_776398, EPI_ISL_776399, EPI_ISL_776408, EPI_ISL_776409, EPI_ISL_776415, EPI_ISL_776423, EPI_ISL_776433, EPI_ISL_776498, EPI_ISL_776502, EPI_ISL_776504, EPI_ISL_776511, EPI_ISL_776515, EPI_ISL_776561			
see above	University Medical Center Hamburg Eppendorf	Heinrich Pette Institute, Leibniz Institute for Experimental Virology	Adam Grundhoff; Alexis Robitaille; Johannes Knobloch; Martin Aepfelbacher; Nicole Fischer; Thomas Günther
EPI_ISL_767043, EPI_ISL_826520	University of Bari Biomedical Sciences and Human Oncology	University of Bari Biomedical Sciences and Human Oncology	Accogli M.; Anna Sallustio; Chironna M; Daniela Loconsole; Loconsole D.; Maria Chironna; Marisa Accogli; Sallustio A.
EPI_ISL_679040	University of Birmingham	COVID-19 Genomics UK (COG-UK)	Alex Richter; Andrew Bosworth; Andrew Bosworth. Queen Elizabeth Hospital: Anna Casey; Andrew D Beggs PHE Heartlands Lab: Husam Osman; Charlotte Poxon; Institute of Microbiology; Joanne Stockton; Josh Quick; Kasun Wanigasooriya; Mike Kidd; Nicholas

		Consortium	Loman. University of Birmingham Testing Laboratory: Celina M Whalley; Oliver Pickles; Radoslaw Poplawski; Samuel Nicholls; University of Birmingham: Claire McMurray; Will Rowe
EPI_ISL_676525	Uppsala klinisk mikrobiologi	The Public Health Agency of Sweden	Department of Microbiology; The Public Health Agency of Sweden
EPI_ISL_574905, EPI_ISL_574927, EPI_ISL_574949, EPI_ISL_729125, EPI_ISL_729190, EPI_ISL_729234, EPI_ISL_729235, EPI_ISL_729236, EPI_ISL_729248, EPI_ISL_737803, EPI_ISL_796522			
see above	Viollier AG	Department of Biosystems Science and Engineering, ETH Zürich	Andrea Patrignani; Andreia Cabral de Gouvea; Catharine Aquino; Chaoran Chen; Christian Beisel; Christiane Beckmann; Christoph Noppen; David Dreifuss; Doris Popovic; Elodie Burcklen; Griffin White; Ina Nissen; Ivan Topolsky; Jay Tracy; Katharina Jahn; Lara Fuhrmann; Laura Neff; Lennart Opitz; Maria Domenica Moccia; Maurice Redondo; Natascha Santacroce; Niko Beerenwinkel; Noemie Santamaria de Souza; Olivier Kobel; Pedro Ferreira; Philipp Jablonski; Ralph Schlapbach; Sarah Nadeau; Simon Grüter; Sophie Seidel; Susana Posada-Céspedes; Tanja Stadler; Timothy Sykes; Tobias Schär
EPI_ISL_705741, EPI_ISL_705785	Virology Department, Royal Infirmary of Edinburgh, NHS Lothian / School of Biological Sciences, University of Edinburgh / Institute of Genetics and Molecular Medicine, University of Edinburgh	COVID-19 Genomics UK (COG-UK) Consortium	Balcaza C; Colquhoun R; Dewar R; Gallagher M; Hill V; Jackson B; McCrone JT; McHugh M; O'Toole Á; Rambaut A; Rooke S; Scher E; Templeton K; Williams TC; Yu X
EPI_ISL_627253	Virology Department, Sheffield Teaching Hospitals NHS Foundation Trust/Department of Infection, Immunity and Cardiovascular Disease, The Medical School, University of Sheffield	COVID-19 Genomics UK (COG-UK) Consortium	Adri Angyal; Alex Keeley; Benjamin Lindsey; Cariad Evans; Danielle Groves; Dave Partridge; Katie Johnson; Laura Carrilero; Luke Green; Matthew Parker; Matthew Wyles; Mehmet Yavuz; Mohammad Raza; Nikki Smith; Paul Parsons; Rachel Tucker; Rebecca Brown; Thushan de Silva
EPI_ISL_613096, EPI_ISL_726260, EPI_ISL_727537, EPI_ISL_727549, EPI_ISL_742374, EPI_ISL_764799, EPI_ISL_764800, EPI_ISL_823565, EPI_ISL_823783			
see above	Wales Specialist Virology Centre Sequencing lab: Pathogen Genomics Unit	COVID-19 Genomics UK (COG-UK) Consortium	Alec Birchley; Alexander Adams; Amy Gaskin; Angela Marchbank; Bree Gatica-Wilcox; Catherine Moore; Jason Coombes; Joanne Watkins; Joel Southgate; Johnathan Evans; Laura Gifford; Lauren Gilbert; Lee Graham; Malorie Perry; Matthew Bull; Nicole Pacchiarini; Sally Corden; Sara Kumziene-Summerhayes; Sara Rey; Sarah Taylor; Simon Cottrell; Sophie Jones; Tom Connor
EPI_ISL_841161	Wales Specialist Virology Centre Sequencing lab: Pathogen Genomics Unit	Public Health Wales Microbiology Cardiff Wales Specialist Virology Centre	Alec Birchley; Alexander Adams; Amy Gaskin; Angela Marchbank; Bree Gatica-Wilcox; Catherine Moore; Jason Coombes; Joanne Watkins; Joel Southgate; Johnathan Evans; Laura Gifford; Lauren Gilbert; Lee Graham; Malorie Perry; Matthew Bull; Nicole Pacchiarini; Sally Corden; Sara Kumziene-Summerhayes; Sara Rey; Sarah Taylor; Simon Cottrell; Sophie Jones; Tom Connor
EPI_ISL_612289, EPI_ISL_814659	West of Scotland Specialist Virology Centre, NHSGGC / MRC-University of Glasgow Centre for Virus Research	COVID-19 Genomics UK (COG-UK) Consortium	Alasdair MacLean; Alice Broos; Ana da Silva Filipe; Antonia Ho; Daniel Mair; David L Robertson; Elihu Aranday-Cortes; Emma Thomson; Guy Mollett; Igor Starinskij; James Shepherd; Jenna Nichols; Joseph Hughes; Kathy Li; Kathy Smollett; Kyriaki Nomikou; Lily Tong; Marc Niebel; Matthew Holden; Natasha Jesudason; Natasha Johnson; Patawee Asamaphan; Rachel Blacow; Rajiv Shah; Richard Orton; Rory Gunson; Sarah McDonald; Sharif Shaaban; Sreenu Vattipally; Stephen Carmichael
EPI_ISL_826771, EPI_ISL_826902, EPI_ISL_827600, EPI_ISL_827602, EPI_ISL_827604, EPI_ISL_827605, EPI_ISL_827610, EPI_ISL_828610, EPI_ISL_828763, EPI_ISL_828767, EPI_ISL_828769, EPI_ISL_828770, EPI_ISL_828778, EPI_ISL_828781, EPI_ISL_828788, EPI_ISL_828799, EPI_ISL_829137			
see above	deCODE genetics	deCODE genetics	Agnar Helgason; Alma Moller; Arna B Agustsdottir; Arnaldur Gylfason; Asgeir Sigurdsson; Aslaug Jonasdottir; Berglind Eiriksdottir; Bjarni Thorbjornsson; Brynjar O Jensson; Daniel F Gudbjartsson; Droplaug N Magnusdottir; Elisabet E Gardarsdottir; Emil A Thorarensen; Gardar Sveinbjornsson; Gisli Masson; Gudmundur Georgsson; Gudmundur L Norddahl; Gudrun Sigmundsdottir; Hakon Jonsson; Hannes Eggertsson; Hilma Holm; Ingileif Jonsdottir; Jona Saemundsdottir; Kamilla S Josefsdottir; Kari Stefansson; Karl G Kristinsson; Kjartan R Gudmundsson; Kristin E Sveinsdottir; Louise le Roux; Maney Sveinsdottir; Olafia S Gretarsdottir; Olafur T Magnusson; Pall Melsted; Patrick Sulem; Run Fridriksdottir; Solvi Rognvaldsson; Thora R Gunnarsdottir; Thordur Kristjansson; Thorolfur Gudnason; Unnur Thorsteinsdottir
EPI_ISL_732885, EPI_ISL_732888, EPI_ISL_732922	genXone SA, Molecular Diagnostics Laboratory / NZOZ	genXone SA, Research & Development Laboratory	Grzegorz Nowicki; Jakub Grabowski; Maciej Sykulski; Michał Kaszuba; Monika Mańkowska-Woźniak; Natalia Drwęska-Matelska; Łukasz Krych

We gratefully acknowledge the following Authors from the Originating laboratories responsible for obtaining the specimens, as well as the Submitting laboratories where the genome data were generated and shared via GISAID, on which this research is based.

All Submitters of data may be contacted directly via www.gisaid.org

Authors are sorted alphabetically.

Accession ID	Originating Laboratory	Submitting Laboratory	Authors
EPI_ISL_699657	1-Laboratory of Microbiology, National Reference Lab, Charles Nicolle Hospital; 2-University of Tunis ElManar, Faculty of Medicine of Tunis, LR99ES09, Tunis, Tunisia	1-Clinical and Experimental Pharmacology Lab, LR16SP02, National Center of Pharmacovigilance, University of Tunis El Manar, Tunis, Tunisia. 2-Neurodegenerative diseases and psychiatric troubles, LR18SP03, Razi Hospital, University of Tunis El Manar, Tunis, Tunisia. 3- Ministry of Health, National Observatory of New and Emerging Diseases, 1006, Tunis, Tunisia	Alia Ben Kahla; Asma Ferjani; Gaies Emna; Guedi Berrabeh; Hanen ElJebari; Ilhem Boutiba-Ben Boubaker; Jalila Ben Khelil; Maher Kharrat; Mouna Ben Sassi; Mouna Safer; Nissaf Ben Alaya; Riadh Daghfous; Riadh Gouider.; Salma Abid; Salwa Mrabet; Sameh Trabelsi; Sana Ferjani
EPI_ISL_542179, EPI_ISL_542225, EPI_ISL_542237	ASST GOM Niguarda	Dep. Of Oncology and Hemato-Oncology University of Milan	Antonio Piralla; Carlo Federico Perno; Chiara Vismara; Claudia Alteri; Elisa Matarazzo; Fausto Baldanti; Federica Giardina; Federica Novazzi; Luna Colagrossi; Maria Antonello; Massimo Puoti; Monica Tallarita; Oscar Massimiliano Epis; Roberto Fumagalli; Silvia Renica; Stefano Gaiarsa; Valentino Costabile; Valeria Cento
EPI_ISL_909962	Apollo Hospitals	CSIR-Centre for Cellular and Molecular Biology	Divya Tej Sowpati; Irawathy Goud; Karthik Bharadwaj Tallapaka; Lamuk Zaveri; Onkar Kulkarni; Payel Mukherjee; Sofia Banu; Suneetha Narreddy
EPI_ISL_509505	Area of Virology, Serology and Virology Division (SAViD), New South Wales Health Pathology Randwick	Area of Virology, Serology and Virology Division (SAViD), New South Wales Health Pathology Randwick	Rawlinson, W.
EPI_ISL_457826	Army Medical Center - Scientific Department	Army Medical and Veterinary Research Center	Anna Anselmo; Antonella Fortunato; Florigio Lista; Francesco Giordani; Giovanni Faggioni; Nino D'Amore; Riccardo De Sanctis; Silvia Fillo; Vanessa Vera Fain
EPI_ISL_457825	Army Medical Research Center - Scientific Department	Army Medical and Veterinary Research Center	Anna Anselmo; Antonella Fortunato; Florigio Lista; Francesco Giordani; Giovanni Faggioni; Nino D'Amore; Riccardo De Sanctis; Silvia Fillo; Vanessa Vera Fain
EPI_ISL_1008158	Austrian Agency for Health and Food Safety (AGES)	Bergthaler laboratory, CeMM Research Center for Molecular Medicine of the Austrian Academy of Sciences	Andreas Bergthaler; Anna Schedl; Bekir Erguner; Benedikt Agerer; Christoph Bock; Jan Laine; Lukas Endler; Maelle Le Moing; Martin Senekowitsch; Michael Schuster; Thomas Penz
EPI_ISL_976824	BCCDC Public Health Laboratory	BCCDC Public Health Laboratory	Ana Pacagnella; Corrinne Ng; Dan Fornika; John Tyson; Kim Macdonald; Kimia Kamelian; Linda Hoang; Loretta Janz; Mel Krajden; Prystajecky Natalie; Robert Azana Terry Snutch; Shannon Russell
EPI_ISL_859907	BTC, Khalifa University	BTC, Khalifa University	Al Safar et al
EPI_ISL_529213	Beijing Institute of Microbiology and Epidemiology	Beijing Institute of Microbiology and Epidemiology	Cui, Y.; Fan; Guo, Y.; Hang; Hou, J.; Li, B.; Mi, Z.; Mu, J.; Qin, E.; Song; Teng; Wu, Y.; Xu, Z.; Yajun.; Yang, R.; Yong, Y.; Yue; Zhang, X.
EPI_ISL_539531	C.H.U Nuestra Señora de Candelaria	Instituto de Salud Carlos III	A. Monzón; F. Casas; I; I. Jiménez; Iglesias-Caballero; M. Camarero; M. Cuesta; M. González-Esguevillas; M. Molinero Calamita; M. Zaballos; O. Díez; P. Jiménez; S. Juliá; S. Pozo; S. Varona
EPI_ISL_644291	CEPHR / Vincent's Hospital	Irish Coronavirus Sequencing Consortium - National Virus Reference Laboratory	Alejandro Abner Garcia Leon; Gabriel Gonzalez; Michael Carr; Patrick Mallon
EPI_ISL_693563	CH Barreiro Montijo	Instituto Nacional de Saude (INSA)	Borges et al
EPI_ISL_644681, EPI_ISL_660706	CHU Montpellier	CNR Virus des Infections Respiratoires - France SUD	Antonin Bal; Bruno Lina; Gregory Destras; Gwendolyne Burfin; Hadrien Règue; Laurence Josset; Martine Valette; Michel Segondy; Quentin Semanas; Vincent Foulongne
EPI_ISL_678541	CNR Virus des Infections Respiratoires - France SUD	CNR Virus des Infections Respiratoires - France SUD	Antonin Bal; Bruno Lina; Gregory Destras; Gwendolyne Burfin; Laurence Josset; Martine Valette; Solenne Brun
EPI_ISL_418235	Cabinet médical	National Reference Center for Viruses of Respiratory Infections, Institut Pasteur, Paris	Angela Brisebarre; Etienne Simon-Lorière; Flora Donati; Marion Barbet; Maud Vanpeene; Mélanie Albert; Méline Bizard; Sylvie Behillil; Sylvie van der Werf; Vincent Enouf
EPI_ISL_438002, EPI_ISL_475798, EPI_ISL_583718	Center for Virology, Medical University of Vienna	Bergthaler laboratory, CeMM Research Center for Molecular Medicine of the Austrian Academy of Sciences	Adi Steinrigl; Alexander Lercher; Alexandra Popa; Andreas Bergthaler; Benedikt Agerer; Christian Paar; Christoph Bock; Daniela Schmid; Dorothee von Laer; Elisabeth Puchhammer-Stoeckl; Franz Allerberger; Gernot Walder; Gregor Hörmann; Guenter Weiss; Gunther Vogl; Henrique Colaco; Jakob-Wendelin Genger; Jan Laine; Judith Aberle; Kinga Rigler-Hohenwarter; Lukas Endler; Manfred Nairz; Mark Smyth; Martin Senekowitsch; Michael Schuster; Peter Hufnagl; Peter Obrist; Rainer Gattringer; Sabine Sussitz-Rack; Stephan Aberle; Thomas Penz; Wegene Borena
EPI_ISL_693480	Central Public Health Laboratory	National Public Health Laboratory, National Centre for Infectious Diseases	Esorom Daoni; Lin Cui; Raymond Tzer Pin Lin; Sophie Octavia; Theresa Palou; Tze Minn Mak; Zhenyang Zhou
EPI_ISL_537828	Centro de Investigación Biomédica de La Rioja - Hospital San Pedro Logroño	SeqCOVID-SPAIN consortium/IBV(CSIC)	José Manuel Azcona Gutiérrez; María Pilar Bea Escudero; María de Toro; Miriam Blasco Alberdi and SeqCOVID-SPAIN consortium
EPI_ISL_753914, EPI_ISL_753983	Charité Universitätsmedizin Berlin, Institut für Virologie/Labor Berlin	Charité Universitätsmedizin Berlin, Institut für Virologie	Barbara Mühlemann; Christian Drosten; Julia Schneider; Jörn Beheim-Schwarzbach; Talitha Veith; Terry Jones; Victor M Corman
EPI_ISL_877693	Clinical Molecular Microbiology Laboratory, UNC Hospital	Dirk Dittmer	Angelica Juarez; Blossom Damania.; Brent A. Eason; Cameroon Grant; Carolina Caro-Vegas; Cecilia Thompson; Dirk Dittmer; Evelyn Hoffman; Jason Wong; Justin T. Landis; Linda Pluta; Melissa B. Miller; Patricio Cano; Razia Moorad
EPI_ISL_527917, EPI_ISL_581806	Clinical Virology	Clinical Bacteriology	Adrian Egli; Alexander Gensch; Alfredo Mari; Christian Nickel; Hans Hirsch; Hans Pargger; Helena MB Seth-Smith; Julia Bielicki; Karoline Leuzinger; Kirstine K. Soegaard; Madlen Stange; Manuel Battegay; Martin Siegemund; Michael Osthoff; Michael Schweitzer; Myrta Brunner; Rita Schneider-SliFemalea; Roland Bingisser; Sarah Tschudin-Sutter; Simon Fuchs; Stefano Bassetti; Tim Roloff
EPI_ISL_745448	DOHMH Crown Heights	New York City Public Health Laboratory	Jade Wang; et al.
EPI_ISL_427385	Department of Clinical Microbiology	GIGA Medical Genomics	Cécile Meex; Keith Durkin; Maria Artesi; Marie-Pierre Hayette; Pierrette Melin; Raphaël Boreux; Sébastien Bontems; Vincent Bours.
EPI_ISL_452075	Department of Clinical Microbiology, Copenhagen University Hospital, Hvidovre, Kettegaard Alle 30, 2650 Hvidovre.	Albertsen lab, Department of Chemistry and Bioscience, Aalborg University, Denmark	Rasmus Kirkegaard
EPI_ISL_487276	Department of Food Safety, Nutrition and Veterinary public health, Istituto Superiore di Sanita'	Department of Biomedical, Surgical and Dental Sciences and Department of Biomedical Sciences for Health	Anselmi, G.; Basilico, N.; Binda, S.; D'Alessandro, S.; Delbue, S.; Ferrante, P.; Galli, C.; Parapini, S.; Pariani, E.; Primache, V.; Signorini, L.
EPI_ISL_457699, EPI_ISL_457700, EPI_ISL_457721, EPI_ISL_457724, EPI_ISL_457728, EPI_ISL_457732, EPI_ISL_457736, EPI_ISL_457749			
see above	Department of Infectious Diseases, Istituto Superiore di Sanità, Roma , Italy	Army Medical and Veterinary Research Center	Alessandra Lo Presti; Anna Anselmo; Antonella Fortunato; Antonella Marchi; Concetta Fabiani Silvia Fillo; Concetta Fabiani Silvia Fillo; Eleonora Benedetti; Florigio Lista; Francesco Giordani; Giovanni Faggioni; Nino D'Amore; Paola Stefanelli; Riccardo De Sanctis; Stefano Fiore; Vanessa Vera Fain
EPI_ISL_412973	Department of Infectious Diseases, Istituto Superiore di Sanità, Roma , Italy	Virology Laboratory, Scientific Department, Army Medical Center	Andrea Ciammaruconi; Anna Anselmo; Antonella Fortunato; Antonella Marchi; Concetta Fabiani; Eleonora Benedetti; Florigio Lista; Giovanni Faggioni; Paola Stefanelli; Riccardo De Santis; Silvia Fillo; Stefano Fiore; Stefano Palomba
EPI_ISL_412974	Department of Infectious Diseases, Istituto Superiore di Sanità, Rome, Italy	Virology Laboratory, Scientific Department, Army Medical Center	Andrea Ciammaruconi; Antonella Fortunato; Antonella Marchi; Concetta Fabiani; Eleonora Benedetti; Filippo Molinari; Florigio Lista; Giancarlo Petralito; Giovanni Faggioni; Paola Stefanelli; Riccardo De Santis; Silvia Fillo; Stefano Fiore
EPI_ISL_668441	Department of Medical Microbiology, St. Olavs hospital	Norwegian Institute of Public Health, Department of Virology	Hilde Elshaug; Hilde Vollan; Kamilla Heddeland Instefjord; Karoline Bragstad; Kathrine Stene-Johansen; Marie Paulsen Madsen; Olav Hungnes; Rasmus Riis Kopperud
EPI_ISL_454733	Department of Medical, Biotechnologies University of Siena	Department of Medical, Biotechnologies University of Siena	Anichini, G.; Cusi; G. and Santoro, F.; Gandolfo, C.; M.G.; Pinzauti, D.; Pozzi
EPI_ISL_425313	Department of Pathology, University of Cambridge	COVID-19 Genomics UK (COG-UK) Consortium	Aminu S. Jahun; Anna Yakovleva; Charlotte J. Houldcroft; Ian Goodfellow; Luke W Meredith; M. Estee Torok; Martin D. Curran; Myra Hosmillo; Sarah L. Caddy; Theresa Feltwell; William L. Hamilton
EPI_ISL_450200, EPI_ISL_450208	Department of Virology	Department of Virology	Ackermann, N.; Antwerpen, M.; Bengs, K.; Berger, A.; Boehm, S.; Boehmer; Boender; Buchholz, U.; Cai, W.; Corman; D.V.; Dangel, A.; Drosten, C.; Eberle, U.; Fingerle, V.; Grahl, A.; Haas, W.; Hamouda, O.; Hoch, M.; Hoermansdorfer, S.; Ippisch, S.; Jones; Katz, K.; Konrad, R.; Liebl, B.; M.M.; Marosevic; Muehlemann, B.; Muller, N.; Poertner, K.; Protzer, U.; Reich, A.; Rexroth, U.; Schneider, J.; Sing, A.; T.C.; T.S.; Treis, B.; V.M.; Veith, T.; Walter, M.; Wicklein, B.; Woelfel, R.; Woudenberg, T.; Zapf, A.; Zeitlmann, N.; an der Heiden, M.
EPI_ISL_413602, EPI_ISL_759776, EPI_ISL_995956, EPI_ISL_1229390, EPI_ISL_1240177	Department of Virology and Immunology, University of Helsinki and Helsinki University Hospital, Huslab Finland	Department of Virology, Faculty of Medicine, University of Helsinki, Helsinki, Finland	Essi Korhonen; Fathiah Zakham; Hanna Jarva; Hanna Liimatainen; Hannimari Kallio-Kokko; Harri Kangas; Hussein Alburkat; Jenni Virtanen; Maija Lappalainen; Maija Suvanto; Mert Erdin; Olli Vapalahti; Pekka Ellonen; Phuoc Truong; Ravi Kant; Sari Hannula; Satu Kurkela; Teemu Smura
EPI_ISL_1015046, EPI_ISL_1015053	Department of Virology, Pitié-Salpêtrière hospital	Department of Virology, Pitié-Salpêtrière hospital	Anne-Geneviève Marcelin; Aude Jary; Karen Zafilaza; Stéphane Marot; Valentin Leducq; Vincent Calvez
EPI_ISL_971085, EPI_ISL_973299	Department of Virus and Microbiological Special Diagnostics, Statens Serum Institut, Copenhagen, Denmark	Aalborg University	Danish Covid-19 Genome Consortium
EPI_ISL_682528, EPI_ISL_682864, EPI_ISL_750815, EPI_ISL_844033	Department of Virus and Microbiological Special Diagnostics, Statens Serum Institut, Copenhagen, Denmark	Albertsen Lab, Department of Chemistry and Bioscience, Aalborg University, Denmark	Danish Covid-19 Genome Consortium
EPI_ISL_444960	Department of Virus and Microbiological Special Diagnostics, Statens Serum Institut, Copenhagen, Denmark, Artillerivej 5, 2300 Copenahgen S	Albertsen lab, Department of Chemistry and Bioscience, Aalborg University, Denmark	Rasmus Kirkegaard
EPI_ISL_416144	Department of Virus and Microbiological Special diagnostics, Statens Serum Institut, Copenhagen, Denmark.	ViFU	Anders Fomsgaard; Maiken Worsoe Rosenstierne; Morten Rasmussen
EPI_ISL_528748	Dinkes Provinsi Jawa Barat	School of Life Sciences and Technology & School of Pharmacy-Institut Teknologi Bandung; Molecular Genetics Laboratory-Faculty of Medicine-Universitas Padjadjaran; Laboratorium Kesehatan Provinsi Jawa Barat	Adelina Khristiani Rahayu; Agung Eru Wibowo; Azzania Fibriani; Catur Riani; Cut Nur Cinthia Alamanda; Ema Rahmawati; Gusti Ayu Prani Pradani; Hammam Riza; Hesti Lina Wiraswati; Husna Nugrahapraja; Irvan Faizal; Lia Faridah; Marselina Irasonia Tan; Rifky Waluyajati Rachman; Ryan Bayusantika Ristandi; Savira Ekawardhani; Sony Solistia Wirawan; Tarwadi; Yunia Sribudiani
EPI_ISL_722858	Dipartimento di Scienze Biomediche e Oncologia Umana - Azienda Ospedaliero Universitaria Consorziale Policlinico	Istituto Zooprofilattico Sperimentale della Puglia e della Basilicata	Bianco A.; Capozzi L.; Chironna M.; Del Sambro L.; Loconsole D.; Parisi A.
EPI_ISL_939672	Division of Infectious Diseases, University Hospital Zürich	Institute of Medical Virology, University of Zurich	Alexandra Trkola; Annette Audigé; Cyril Shah; Guido Bloemberg; Jon Huder; Jürg Böni; Kevin Steiner; Maria Grünberg; Maryam Zaheri; Michael Huber; Riccarda Capaul; Stefan Schmutz; Verena Kufner

EPI_ISL_422796, EPI_ISL_460999, EPI_ISL_722645	Dutch COVID-19 response team	Erasmus Medical Center	Anne van der Linden; Annemiek van der Eijk; Aura Timen; Bas Oude Munnink; Claudia Schapendonk; Corien Swaan; Corine GeurtsvanKessel; David Nieuwenhuijse; Emmanuelle Munger; Irina Chestakova; Jeroen van Kampen; Jolanda Voermans; Madelief Mollers; Manon Haverkate; Marion Koopmans; Marjan Boter; Mark Pronk; Mart Stein; Pascal Lexmond; Reina Sikkema; Richard Molenkamp; Sandra Kengne Kamga Mobou; Stefan van Nieuwkoop; Theo Bestebroer; on behalf of the Dutch national COVID-19 response team.
EPI_ISL_547511, EPI_ISL_905379	Dutch COVID-19 response team	National Institute for Public Health and the Environment (RIVM)	Adam Meijer; AnneMarie van den Brandt; Bas van der Veer; Chantal Reusken; Dennis Schmitz; Dirk Eggink; Florian Zwagemaker; Harry Vennema; Jeroen Cremer; Sharon van den Brink; on behalf of the national COVID-19 response team
EPI_ISL_683702	Essentia Health-St. Mary's Medical Center	Minnesota Department of Health, Public Health Laboratory	Alexandra Lorentz; Jacob Garfin; Matt Plumb; and Xiong Wang
EPI_ISL_594380	Florida Bureau of Public Health Laboratories	Florida Bureau of Public Health Laboratories	Jason Blanton; Sarah Schmedes
EPI_ISL_411060	Fujian Center for Disease Control and Prevention	Fujian Center for Disease Control and Prevention	Chen Wei; He Wenxiang; Weng Yuwei; Zhang Yanhua
EPI_ISL_900539	Gen-Bio	CNR Virus des Infections Respiratoires - France SUD	Antonin Bal; Bruno Lina; Gregory Destras; Gwendolyne Burfin; Hadrien Règue; Laurence Josset; Martine Valette; Quentin Semanas
EPI_ISL_406801	General Hospital of Central Theater Command of People's Liberation Army of China	BGI & Institute of Microbiology, Chinese Academy of Sciences & Shandong First Medical University & Shandong Academy of Medical Sciences & General Hospital of Central Theater Command of People's Liberation Army of China	Weifeng Shi and Zhenhong Hu; Weijun Chen; Yuhai Bi
EPI_ISL_444969	Guangzhou Eighth People's Hospital (Jiahe Sector)	Institute of Human Virology, Zhongshan School of Medicine, Sun Yat-sen University	Bingfeng Liu; Fang Li; Fei Yu; Feng Huang; Fengyu Hu; Hui Zhang; Huimin Fan; Jun Liu; Junsong Zhang; Kai Deng; Mang Shi; Ruosu Ying; Ting Pan; Xu Zhang; Yiwen Zhang
EPI_ISL_700512	Guguletu CHC wc GDH	NHLS/UCT	Arash Iranzadeh; Bruna Galvao; Carolyn Williamson; Deelan Doolabh; Diana Hardie; Innocent Mudau; Kruger Marais; Lynn Tyers; Marvin Hsiao; Stephen Korsman
EPI_ISL_872988	HELIX LLC	WHO National Influenza Centre Russian Federation	Andrey Komissarov; Anna Ivanova; Artem Fadeev; Daria Danilenko; Dmitry Bazhenov; Dmitry Lioznov; Elena Nabieva; Georgii Bazykin; Ksenia Safina; Kseniya Komissarova; Mikhail Bakaev
EPI_ISL_482577	Hangzhou Center for Diseases Control and Prevention	Hangzhou Center for Diseases Control and Prevention	Haoqiu Wang; Hua Yu; Jun Li; Junfang Chen; Lingfeng Mao; Shuchang Chen; Xin Qian; Xinfen Yu; Xuchu Wang; Zhou Sun
EPI_ISL_832138	Hospital	National Reference Center for Viruses of Respiratory Infections, Institut Pasteur, Paris	Angela Brisebarre; Camille Capel; Etienne Simon-Lorière; Laurent Andreoletti; Marion Barbet; Maud Vanpeene; Méline Bizard; Sylvie Behillil; Sylvie van der Werf; Vincent Enouf
EPI_ISL_539548	Hospital Clínic	Instituto de Salud Carlos III	A. Monzón; F. Casas; I; I. Jiménez; Iglesias-Caballero; M. Camarero; M. Cuesta; M. González-Esguevillas; M. Molinero Calamita; M. Zaballos; M.A Marcos; P. Jiménez; S. Juliá; S. Pozo; S. Varona
EPI_ISL_871988	Hospital Clínico San Carlos	Instituto de Salud Carlos III	A. Monzón; F. Casas; I. Jiménez; I. Rodríguez, I.; Iglesias-Caballero; M. Camarero; M. Cuesta; M. González-Esguevillas; M. Pozo; M. Zaballos; P. Jiménez; S. Juliá; S. Molinero Calamita; S. Varona
EPI_ISL_481094, EPI_ISL_654339	Hospital General Universitario Gregorio Marañón	SeqCOVID-SPAIN consortium/IBV(CSIC)	Darío García de Viedma; Darío García de Viedma and SeqCOVID-SPAIN consortium; Jon Sicilia; Julia Suárez; Laura Pérez-Lago; Marta Herranz; Patricia Muñoz; Patricia Muñoz and SeqCOVID-SPAIN consortium; Pilar Catalán
EPI_ISL_412964	Hospital Israelita Albert Einstein	Instituto Adolfo Lutz Interdisciplinary Procedures Center Strategic Laboratory	Andrew Rambaut; Claudia Regina Gonçalves; Claudio Tavares Sacchi; Daniela Bernardes Borges da Silva; Ester Cerdeira Sabino; Flávia Cristina da Silva Sales; Ingra Morales Claro; Jaqueline Goes de Jesus; Joshua Quick; Maria do Carmo; Nicholas James Loman; Nuno Rodrigues Faria; Sampaio Tavares Timenetsky
EPI_ISL_444989	Hospital Universitari Vall d'Hebron - Vall d'Hebron Institut de Recerca	Hospital Universitari Vall d'Hebron - Vall d'Hebron Institut de Recerca	Andrés Antón; Ariadna Rando; Cristina Andrés; Damir Garcia-Cehic; Josep Quer; Juliana Esperalba; Maria Gema Codina; Maria Piñana; Mercedes Guerrero-Murillo; Tomàs Pumarola
EPI_ISL_452728	Hospital Universitario Araba. Vitoria-Gasteiz,	SeqCOVID-SPAIN consortium/IBV(CSIC)	Amaia Aguirre Quiñonero; Andrés Canut Blasco and SeqCOVID-SPAIN consortium; Carmen Gómez González; Maria Concepción Lecaroz Agara; Maria Rosario Almela Ferrer; Marina Fernández Torres; Silvia Hernáez Crespo
EPI_ISL_530084	Hospital Universitario La Paz	Hospital Universitario La Paz	Elias Dahdouh; Esther Viedma; Fernando Lázaro; Jesús Mingorance; Juan Carlos Galán; Julio García; María Rodríguez; Mª Dolores Folgueira; Natalia Stella; Rafael Cantón; Rafael Delgado; Raúl Recio; Sara González
EPI_ISL_831265	Hospital Universitario La Paz (Madrid)	SeqCOVID-SPAIN consortium/IBV(CSIC)	Elias Dahdouh; Fernando Lázaro-Perona; Jesús Mingorance and SeqCOVID-SPAIN consortium; María Rodríguez-Tejedor
EPI_ISL_544641, EPI_ISL_545133, EPI_ISL_786478, EPI_ISL_787988, EPI_ISL_788610, EPI_ISL_1075581	Houston Methodist Hospital	Houston Methodist Hospital	Chia-Wei Chou; Concepcion C. Cantu; Daniel Boutz; David W. Bernard; Ghazaleh Eskandari; Heather Hendrickson; Hoang A. T. Nguyen; Hung-Che Kuo; Ilya J. Finkelstein; J. Hunter Long; James J. Davis; Jason S. McLellan; Jessica Cambric; Jimmy Gollihar; Jule Goike; Kamyab Javanmardi; Kristina Reppond; Layne Pruitt; Madison N. Shyer; Marcus Nguyen; Matthew Ojeda Saavedra; Maulik Shukla; Muthiah Kumaraswami; Paul A. Christensen; Prasanti Yerramilli; Randall J. Olsen; Robert Olson; S. Wesley Long; Sishir Subedi; and James M. Musser
EPI_ISL_940210	Hôpital Bichat Claude Bernard, Laboratoire de Virologie	IAME UMR1137 Inserm, Université de Paris, Hôpital Bichat	Alexandre Storto; Amélie Recoing; Antoine Bridier-Nahmias; Benoit Visseaux; Charlotte Charpentier; Diane Descamps; Gilles Collin; Lena Daniel; Mélanie Bertine; Nadhira Houhou-Fidouh; Quentin Le Hingrat; Siham Hamri
EPI_ISL_455660	ICMR-National Institute of Cholera and Enteric Diseases	National Institute of Biomedical Genomics	Ananya Chatterjee; Arindam Maitra; Hasina Banu; Mamta Chawla Sarkar; Saumitra Das; Shanta Dutta; Sreedhar Chinnaswamy
EPI_ISL_419255	INMI Lazzaro Spallanzani IRCCS	INMI Lazzaro Spallanzani IRCCS	Antonino Di Caro; Barbara Bartolini; Cesare E. M. Gruber; Concetta Castilletti; Daniele Lapa; Eleonora Lalle; Emanuela Giombini; Fabrizio Carletti; Francesca Colavita; Francesco Messina; Giuseppe Ippolito.; Maria R. Capobianchi; Martina Rueca
EPI_ISL_410545, EPI_ISL_410546, EPI_ISL_417921, EPI_ISL_417922, EPI_ISL_417923, EPI_ISL_419254, EPI_ISL_424342, EPI_ISL_424343, EPI_ISL_424344			
see above	INMI Lazzaro Spallanzani IRCCS	Laboratory of Virology, INMI Lazzaro Spallanzani IRCCS	Antonino Di Caro; Barbara Bartolini; Cesare E. M. Gruber; Cesare Ernesto Maria Gruber; Concetta Castilletti; Daniele Lapa; Eleonora Lalle; Emanuela Giombini; Emanuele Nicastri; Fabrizio Carletti; Francesca Colavita; Francesco Messina; Francesco Vairo; Giulia Matusali; Giuseppe Ippolito; Giuseppe Ippolito.; Licia Bordi; Maria R. Capobianchi; Maria Rosaria Capobianchi; Martina Rueca
EPI_ISL_437932, EPI_ISL_437977	Institut für Virologie am Department für Hygiene, Mikrobiologie und Public Health	Bergthaler laboratory, CeMM Research Center for Molecular Medicine of the Austrian Academy of Sciences	Alexander Lercher; Alexandra Popa; Andreas Bergthaler; Benedikt Agerer; Christoph Bock; Dorothee von Laer; Elisabeth Puchhammer-Stoeckl; Guenter Weiss; Henrique Colaco; Jakob-Wendelin Genger; Jan Laine; Judith Aberle; Lukas Endler; Manfred Nairz; Mark Smyth; Martin Senekowitsch; Michael Schuster; Stephan Aberle; Thomas Penz; Wegene Borena
EPI_ISL_626220	Institute for Virology, University Hospital Essen	Center of Medical Microbiology, Virology, and Hospital Hygiene, University of Duesseldorf	Alexander Dilthey; Andreas Walker; Daniel Strelow; Jessica Nicolai; Jörg Timm; Klaus Pfeffer; Lisanna Hülse; Malte Kohns Vasconcelos; Maximilian Damagnez; Nadine Lübke; Olympia E. Anastasiou; Tobias Wienemann; Torsten Houwaart; Ulf Dittmer
EPI_ISL_728204	Institute of Microbiology, Universidad San Francisco de Quito	Institute of Microbiology, Universidad San Francisco de Quito	Belén Prado-Vivar; Bernardo Gutiérrez; Gabriel Trueba; Juan José Guadalupe; Michelle Grunauer; Monica Becerra-Wong; Patricio Reyes; Patricio Rojas-Silva; Paúl Cárdenas; Sully Márquez; Tania Guayasamin; Verónica Barragán
EPI_ISL_402123	Institute of Pathogen Biology, Chinese Academy of Medical Sciences & Peking Union Medical College	Institute of Pathogen Biology, Chinese Academy of Medical Sciences & Peking Union Medical College	Chao Wu; Jianwei Wang; Lili Ren; Qi Jin; Yiwei Liu; Zhiqiang Wu; Zichun Xiang
EPI_ISL_852733	Institute of Virology, Medical Center, University of Freiburg, Freiburg, Germany	Institute of Virology, Clinial Virus Genomics, Medical Center, University of Freiburg, Freiburg, Germany	Hajo Grundmann; Jonas Fuchs; Lisa Kern; Marcus Panning; Sandra Reuter
EPI_ISL_755646	Instituto Adolfo Lutz - Regional de Santo Andre	Instituto Adolfo Lutz, Interdiciplinary Procedures Center, Strategic Laboratory	Claudia Regina Gonçalves; Claudio Tavares Sacchi; Erica Valessa Ramos Gomes; Karoline Rodrigues Campos
EPI_ISL_491230	Instituto Gulbenkian de Ciência	Instituto Gulbenkian de Ciência	Cathy Paulino; Joao Sobral; João Costa; Ricardo Leite; Susana Ladeiro
EPI_ISL_477193, EPI_ISL_477194, EPI_ISL_477202, EPI_ISL_477203	Istituto Zooprofilattico Sperimentale Puglia e Basilicata;	Beaconlab (Bioinformatics, Evolution and Comparative Genomics lab), Dept of Biosciences, University on Mila	Chiara M.; Manzari C.; Parisi A.; Pesole G.
EPI_ISL_477195, EPI_ISL_477196, EPI_ISL_477197, EPI_ISL_477198, EPI_ISL_477199, EPI_ISL_477200, EPI_ISL_477201			
see above	Istituto Zooprofilattico Sperimentale Puglia e Basilicata;	Beaconlab (Bioinformatics, Evolution and Comparative Genomics lab), Dept of Biosciences, University on Milan	Chiara M.; Manzari C.; Parisi A.; Pesole G.
EPI_ISL_451961	Istituto Zooprofilattico Sperimentale Puglia e Basilicata; Dipartimento di Bioscienze, Biotecnologie e Biofarmaceutica dell'Università degli Studi di Bari "A.Moro"; Istituto di Biomembrane. Bioenergetica e Biotecnologie Molecolari del Consiglio Nazionale delle Ricerche di Bari	Beaconlab (Bioinformatics Evolution and Comparative Genomics lab), Dept of Biosciences, University of Milan	Chiara M.; Manzari C.; Parisi A.; Pesole G.
EPI_ISL_451962	Istituto Zooprofilattico Sperimentale Puglia e Basilicata; Dipartimento di Bioscienze, Biotecnologie e Biofarmaceutica dell'Università degli Studi di Bari "A.Moro"; Istituto di Biomembrane. Bioenergetica e Biotecnologie Molecolari del Consiglio Nazionale delle Ricerche di Bari	Beaconlab (Bioinformatics, Evolution and Comparative Genomics lab), Dept of Biosciences, University on Milan	Chiara M.; Manzari C.; Parisi A.; Pesole G.
EPI_ISL_468914, EPI_ISL_469016, EPI_ISL_469018, EPI_ISL_469019, EPI_ISL_469020, EPI_ISL_469021, EPI_ISL_469022, EPI_ISL_469023, EPI_ISL_469050			
see above	Istituto Zooprofilattico Sperimentale Puglia e Basilicata; Dipartimento di Bioscienze, Biotecnologie e Biofarmaceutica dell'Università degli Studi di Bari "A.Moro"; Istituto di Biomembrane. Bioenergetica e Biotecnologie Molecolari del Consiglio Nazionale delle Ricerche di Bari	Beaconlab (Bioinformatics, Evolution and Comparative Genomics lab), Dept of Biosciences, University on Milan	Chiara M; Chiara M.; Manzari C.; Parisi A.; Pesole G.
EPI_ISL_415159	KU Leuven, Clinical and Epidemiological Virology	KU Leuven, Clinical and Epidemiological Virology	Bert Vanmechelen; Joan Marti-Carreras; Piet Maes; Tony Wawina
EPI_ISL_458215, EPI_ISL_896481	KU Leuven, Rega Institute, Clinical and Epidemiological Virology	KU Leuven, Rega Institute, Clinical and Epidemiological Virology	Bert Vanmechelen; Joan Marti-Carerras; Piet Maes; Tony Wawina-Bokalanga
EPI_ISL_915362	Keio University School of Medicine	Keio University School of Medicine	Haruhiko Siomi; Hirotsugu Ishizu; Kenjiro Kosaki; Kodai Abe; Yuka Iwasaki
EPI_ISL_700464	Khayelethu Clinic wc KLC	NHLS/UCT	Arash Iranzadeh; Bruna Galvao; Carolyn Williamson; Deelan Doolabh; Diana Hardie; Houriiyah Tegally; Innocent Mudau; Kruger Marais; Lynn Tyers; Marvin Hsiao; Stephen Korsman
EPI_ISL_419296	Kochi Prefectural Institute of Public Health	Pathogen Genomics Center, National Institute of Infectious Diseases	Akihiko Tokaji; Hajime Kamiya; Kentaro Itokawa; Makoto Kuroda; Masanori Hashino; Motoi Suzuki; Rina Tanaka; Tsuyoshi Sekizuka
EPI_ISL_918504	LACEN - Laboratório Central de Saúde Pública do Amazonas	Evandro Chagas Institute	A.M.; Barbagelata; E.C.; E.M.A.; Ferreira; J.A.; Junior; K.C.; L.C.; L.S.; M.C.; P.S.; Pinheiro; Santos; Silva; Sousa; Sousa Junior; W.D.C.; da Silva
EPI_ISL_653377	LSUHS Emerging Viral Threat Laboratory	Microbial Genome Sequencing Center	Andrew D. Yurochko; Christopher G. Kevil; Daniel J. Snyder; Jeremy P. Kamil; John A. Vanchiere; Katarzyna Zwolinska; Maarten Van Diest; Malgorzata Bienkowska-Haba; Martin J. Sapp; Rona S. Scott; Vaughn S. Cooper
EPI_ISL_755631	LabPLUS	Institute of Environmental Science and Research (ESR)	Anja Werno; Antje van der Linden; Arlo Upton; Chris Mansell; David Hammer; Dragana Drinkovic; Erasmus Smit; Gary McAuliffe; Hana Sofia Andersson; Hermes Perez; James Ussher; Jill Sherwood; Jing Wang; Joep de Ligt; Josh Freeman; Julia Howard; Juliet Elvy; Lauren Jelly; Mary DeAlmeida; Matt Blakiston; Matt Storey; Matthew Rogers; Max Bloomfield; Michael Addidle; Michelle Balm; Muhammad Faisal; Nikki Freed; Olin Silander; Sally Roberts; Sarah Jefferies; Sharmini Muttaiyah; Susan Morpeth; Susan Taylor; Timothy Blackmore; Vani Sathyendran; Veronica Playle; Virginia Hope; Xiaoyun Ren
EPI_ISL_413996	Laboratoire de Virologie, HUG	Swiss National Reference Centre for Influenza	LAUBSCHER Florian et al.
EPI_ISL_890119	Laboratoire de santé publique du Québec	Laboratoire de santé publique du Québec	Guillaume Bourque; Ioannis Ragoussis; Jesse Shapiro; Mark Lathrop and Michel Roger on behalf of the CoVSeQ research group; Sandrine Moreira

EPI_ISL_629084	Laboratoire du Centre Hospitalier Annecy Genevois	CNR Virus des Infections Respiratoires - France SUD	Antonin Bal; Bruno Chanzy; Bruno Lina; Gregory Destras; Gwendolyne Burfin; Hadrien Règue; Hélène Petitprez; Laurence Josset; Martine Valette; Quentin Semanas
EPI_ISL_458085	Laboratorio Biologia Molecolare Sars Cov2 - UOC Laboratorio Analisi - Servizio Medicina di Laboratorio , Ospedale "San Francesco" - ATS- ASSL Nuoro	Laboratorio specialistico UOC Ematologia - Ospedale "San Francesco" - ATS-ASSL Nuoro	Asproni Rosanna; Casu Gavino; Fancello Tatiana; Fiamma Maura; Floris Anna Rita; Lo Maglio Iana; Mameli Giuseppe.; Monne Maria Itria; Palmas Angelo Domenico; Piras Giovanna; Sanna Filomena; Sulis Vincenzo; Toja Alessandro
EPI_ISL_458084	Laboratorio Biologia Molecolare Sars Cov2 - UOC Laboratorio Analisi - Servizio Medicina di Laboratorio, Ospedale "San Francesco" - ATS-ASSL Nuoro	Laboratorio specialistico UOC Ematologia - Ospedale "San Francesco" - ATS-ASSL Nuoro	Asproni Rosanna; Casu Gavino; Fancello Tatiana; Fiamma Maura; Floris Anna Rita; Lo Maglio Iana; Mameli Giuseppe.; Monne Maria Itria; Palmas Angelo Domenico; Piras Giovanna; Sanna Filomena; Sulis Vincenzo; Toja Alessandro
EPI_ISL_413489	Laboratorio di Microbiologia e Virologia, Università Vita-Salute San Raffaele, Milano	Laboratorio di Microbiologia e Virologia, Università Vita-Salute San Raffaele, Milano	C. Di Resta; E. Boeri; E. Criscuolo; G. Lo Raso; I. Negri; M. Castelli; M. Clementi; M. Sampaolo; N. Mancini & N. Clementi; R. Burioni; R. Ferrarese; R.A Diotti; V. Amato; V. Caputo
EPI_ISL_417445, EPI_ISL_417447	Laboratory of Infectious Diseases, Department of Biomedical and Clinical Sciences L. Sacco, University of Milan	Laboratory of Infectious Diseases, Department of Biomedical and Clinical Sciences L. Sacco, University of Milan	Agostino Riva; Alessia Lai; Annalisa Bergna; Arianna Gabrieli; Claudia Balotta; Dario Bernacchia; Gianguglielmo Zehender; Giuliano Rizzardini; Luca Meroni; Maciej Tarkowski; Massimo Galli; Spinello Antinori; Stefano Rusconi
EPI_ISL_434468	Laboratory of Microbiology, Medical School, National and Kapodistrian University of Athens	Laboratory of Biology, Department of Medicine, Democritus University of Thrace	Bampali, M.; Dovrolis, N.; Froukala, E.; Gatzidou, E.; Kassela K.; N. and Karakasiliotis, I.; Spanakis; Stavropoulou, A.; Tsakris, A.; Veletza, S.
EPI_ISL_428853	Laboratory of Molecular Virology International Center for Genetic Engineering and Biotechnology (ICGEB)	ARGO Open Lab Platform for Genome Sequencing	D'Agaro P; Dal Monego S; Licastro D; Marcello A; Rajasekharan S; Segat L
EPI_ISL_417419, EPI_ISL_417421, EPI_ISL_417423, EPI_ISL_428854	Laboratory of Molecular Virology International Center for Genetic Engineering and Biotechnology (ICGEB)	ARGO Open Lab Platform for Genome sequencing	D'Agaro P; Dal Monego S; Licastro D; Marcello A; Rajasekharan; Rajasekharan S; Segat L
EPI_ISL_417418	Laboratory of Molecular Virology International Center fro Genetic Engineering and Biotechnology (ICGEB)	ARGO Open Lab Platform for Genome sequencing	D'Agaro P; Dal Monego S; Licastro D; Marcello A; Rajasekharan S; Segat L
EPI_ISL_479616, EPI_ISL_479617, EPI_ISL_479618, EPI_ISL_479619, EPI_ISL_479790, EPI_ISL_479791	Laboratory of Molecular Virology of the International Centre for Genetic Engineering and Biotechnology (ICGEB)	ARGO Open Lab Platform for Genome Sequencing	Confalonieri M; Confalonieri M Marcello A; Confalonieri P; D; D'Agaro P; Dal Monego S; Licastro; Marcello A; Rajasekharan S; Salton F; Segat L
EPI_ISL_451298, EPI_ISL_451299, EPI_ISL_451300, EPI_ISL_451301, EPI_ISL_451302, EPI_ISL_451303, EPI_ISL_451304			
see above	Laboratory of Virology, INMI Lazzaro Spallanzani IRCCS	Laboratory of Virology, INMI Lazzaro Spallanzani IRCCS	Antonino Di Caro; Barbara Bartolini; Cesare E.M. Gruber; Francesco Messina; Giuseppe Ippolito; Maria R. Capobianchi; Martina Rueca
EPI_ISL_427292	Laboratório Central de Saúde Pública do Estado de Alagoas (LACEN-AL)	Laboratory of Respiratory Viruses and Measles, Oswaldo Cruz Institute, FIOCRUZ	Aline Mattos; Braulia Caetano; Cristiana Garcia; Fernando Motta; Jonathan Lopes; Luciana Appolinario; Maria Ogrzewalska; Marilda Siqueira on behalf of the Fiocruz COVID-19 Genomic Surveillance Network; Milene Miranda; Paola Resende; Priscila Born; Sunando Roy
EPI_ISL_539169	Leeds Teaching Hospitals NHS Trust and Public Health England, National Infection Service (Leeds laboratory)	Wellcome Sanger Institute for the COVID-19 Genomics UK (COG-UK) consortium	Antony Hale and Alex Alderton; Cordelia Langford; David K. Jackson; Dominic Kwiatkowski; Ewan Harrison; Holli Carden; Ian Johnston; John Sillitoe on behalf of the Wellcome Sanger Institute COVID-19 Surveillance Team; Katherine L. Harper; Louissa Macfarlane-Smith; Roberto Amato; Sonia Goncalves
EPI_ISL_820362, EPI_ISL_878414	Lighthouse Lab in Alderley Park	Wellcome Sanger Institute for the COVID-19 Genomics UK (COG-UK) Consortium	Cordelia Langford; David K. Jackson; Dominic Kwiatkowski; Ewan Harrison; Ian Johnston; Jacquelyn Wynn; John Sillitoe on behalf of the Wellcome Sanger Institute COVID-19 Surveillance Team; Mairead Hyland; Roberto Amato; Sonia Goncalves; The Lighthouse Lab in Alderley Park and Alex Alderton
EPI_ISL_673451, EPI_ISL_945170	Lighthouse Lab in Cambridge	Wellcome Sanger Institute for the COVID-19 Genomics UK (COG-UK) Consortium	Cordelia Langford; David K. Jackson; Dominic Kwiatkowski; Ewan Harrison; Ian Johnston; John Sillitoe on behalf of the Wellcome Sanger Institute COVID-19 Surveillance Team; Rob Howes; Roberto Amato; Sonia Goncalves; The Lighthouse Lab in Cambridge and Alex Alderton
EPI_ISL_556482	Lighthouse Lab in Cambridge	Wellcome Sanger Institute for the COVID-19 Genomics UK (COG-UK) consortium	Cordelia Langford; David K. Jackson; Dominic Kwiatkowski; Ewan Harrison; Ian Johnston; John Sillitoe on behalf of the Wellcome Sanger Institute COVID-19 Surveillance Team; Rob Howes; Roberto Amato; Sonia Goncalves; The Lighthouse Lab in Cambridge and Alex Alderton
EPI_ISL_760452	Lighthouse Lab in Glasgow	Wellcome Sanger Institute for the COVID-19 Genomics UK (COG-UK) Consortium	Anna Dominiczak and Alex Alderton; Carol Clugston; Cordelia Langford; David Gray; David K. Jackson; Dominic Kwiatkowski; Ewan Harrison; Harper VanSteenhouse; Ian Johnston; John Sillitoe on behalf of the Wellcome Sanger Institute COVID-19 Surveillance Team; Roberto Amato; Sonia Goncalves; Yumi Kasai
EPI_ISL_532970, EPI_ISL_589997, EPI_ISL_600995	Lighthouse Lab in Glasgow	Wellcome Sanger Institute for the COVID-19 Genomics UK (COG-UK) consortium	Anna Dominiczak and Alex Alderton; Carol Clugston; Cordelia Langford; David Gray; David K. Jackson; Dominic Kwiatkowski; Ewan Harrison; Harper VanSteenhouse; Ian Johnston; John Sillitoe; John Sillitoe on behalf of the Wellcome Sanger Institute COVID-19 Surveillance Team (http://www.sanger.ac.uk/covid-team); Roberto Amato; Sonia Goncalves; Yumi Kasai
EPI_ISL_777085	Lighthouse Lab in Milton Keynes	Wellcome Sanger Institute for the COVID-19 Genomics UK (COG-UK) Consortium	Cordelia Langford; David K. Jackson; Dominic Kwiatkowski; Ewan Harrison; Ian Johnston; John Sillitoe on behalf of the Wellcome Sanger Institute COVID-19 Surveillance Team; Roberto Amato; Sonia Goncalves; The Lighthouse Lab in Milton Keynes and Alex Alderton
EPI_ISL_557984, EPI_ISL_601443	Lighthouse Lab in Milton Keynes	Wellcome Sanger Institute for the COVID-19 Genomics UK (COG-UK) consortium	Cordelia Langford; David K. Jackson; Dominic Kwiatkowski; Ewan Harrison; Ian Johnston; John Sillitoe on behalf of the Wellcome Sanger Institute COVID-19 Surveillance Team (http://www.sanger.ac.uk/covid-team); Roberto Amato; Sonia Goncalves; The Lighthouse Lab in Milton Keynes and Alex Alderton
EPI_ISL_466901	Max von Pettenkofer Institute, Virology, National Reference Center for Retroviruses, LMU München	Laboratory for Functional Genome Analysis, Dept. Genomics, Gene Center of the LMU Munich	Alexander Graf; Helmut Blum; Max Muenchhoff; Oliver Keppler; Stefan Krebs
EPI_ISL_486646, EPI_ISL_486647, EPI_ISL_486648, EPI_ISL_486649, EPI_ISL_486650, EPI_ISL_486651, EPI_ISL_486652, EPI_ISL_486653, EPI_ISL_486654, EPI_ISL_486655, EPI_ISL_486656, EPI_ISL_486657, EPI_ISL_486658, EPI_ISL_486659, EPI_ISL_486660, EPI_ISL_486661, EPI_ISL_486662, EPI_ISL_486663, EPI_ISL_486664, EPI_ISL_486665			
see above	Microbiology, Virology and Biemergency Laboratory-ASST FBF Sacco	Microbiology, Virology and Biemergency Laboratory-ASST FBF Sacco	Comandatore F; Mancon A; Micheli V; Rimoldi SG; Romeri F
EPI_ISL_487340	Molecular Diagnostics Services (MDS)	KRISP, KZN Research Innovation and Sequencing Platform	Chimukangara B; Giandhari J; Khan S; Lessells R; Mdlalose K; Pillay S; Tegally H; Wilkinson E; York D; de Oliveira T
EPI_ISL_451306, EPI_ISL_451307, EPI_ISL_451308, EPI_ISL_451309, EPI_ISL_460079, EPI_ISL_460080, EPI_ISL_460081, EPI_ISL_460082, EPI_ISL_460083, EPI_ISL_460084, EPI_ISL_460085, EPI_ISL_460086, EPI_ISL_460087, EPI_ISL_460088, EPI_ISL_460089, EPI_ISL_460090, EPI_ISL_460091, EPI_ISL_460092, EPI_ISL_460093, EPI_ISL_460094, EPI_ISL_460095			
see above	Molecular Virology Unit, Fondazione IRCCS Policlinico San Matteo , Pavia	Laboratory of Virology, INMI Lazzaro Spallanzani IRCCS	Antonino Di Caro; Antonio Piralla; Barbara Bartolini; Cesare E.M. Gruber; Fausto Baldanti; Maria R. Capobianchi; Martina Rueca
EPI_ISL_602665	NHLS-IALCH	KRISP, KZN Research Innovation and Sequencing Platform	Giandhari J; Khan S; Lessells R; Mdlalose K; Pillay S; Tegally H; Wilkinson E; York D; de Oliveira T
EPI_ISL_860626	NHLS-IALCH	KRISP, KZn Research Innovation and Sequencing Platform	Giandhari J; Khan S; Lessells R; Mdlalose K; Pillay S; Tegally H; Wilkinson E; York D; de Oliveira T
EPI_ISL_489654, EPI_ISL_532905	NHSGGC West of Scotland Specialist Virology Centre / MRC-University of Glasgow Centre for Virus Research	Wellcome Sanger Institute for the COVID-19 Genomics UK (COG-UK) consortium	Alasdair MacLean; Alice Broos; Ana da Silva Filipe; Antonia Ho; Cordelia Langford; Daniel Mair; David K. Jackson; David L Robertson; Dominic Kwiatkowski; Elihu Aranday-Cortes; Emma Thomson and Alex Alderton; Ewan Harrison; Ian Johnston; James Shepherd; Jenna Nichols; John Sillitoe; John Sillitoe on behalf of the Wellcome Sanger Institute COVID-19 Surveillance Team (http://www.sanger.ac.uk/covid-team); Joseph Hughes; Kathy Li; Kathy Smollett; Kirstyn Brunker; Kyriaki Nomikou; Lily Tong; Marc Niebel; Natasha Jesudason; Natasha Johnson; Patawee Asamaphan; Rajiv Shah; Richard Orton; Roberto Amato; Rory Gunson; Sarah McDonald; Sonia Goncalves; Sreenu Vattipally; Stephen Carmichael; Yasmin Parr
EPI_ISL_766866	NIC Viral Respiratory Unit - Institut Pasteur of Algeria	National Reference Center for Viruses of Respiratory Infections, Institut Pasteur, Paris	Angela Brisebarre; Etienne Simon-Lorière; Fawzi Derrar; Flora Donati; Marion Barbet; Maud Vanpeene; Mélanie Albert; Méline Bizard; Sylvie Behillil; Sylvie van der Werf; Vincent Enouf
EPI_ISL_420793	NYC Department of Health and Mental Hygiene	Pathogen Discovery, Respiratory Viruses Branch, Division of Viral Diseases, Centers for Disease Control and Prevention	Alison S. Laufer Halpin; Anne Uehara; Christopher A. Elkins; Clinton R. Paden; Haibin Wang; Jasmine Padilla; Jing Zhang; Justin Lee; Krista Queen; Mary S. Keckler; Rachel Marine; Suxiang Tong; Yan Li; Ying Tao
EPI_ISL_469060	Narhalsan Sjobo vardcentral	The Public Health Agency of Sweden	Anna Risberg; Anna-Malin Linde; Karin Tegmark-Wisell; Maria Lind Karlberg; Mattias Haukland; Olov Svartstrom; Oskar Karlsson Lindsjo; Petra Edquist; Reza Advani; Sandra Broddesson; Shamam Muradrasoli
EPI_ISL_402125	National Institute for Communicable Disease Control and Prevention (ICDC) Chinese Center for Disease Control and Prevention (China CDC)	National Institute for Communicable Disease Control and Prevention (ICDC) Chinese Center for Disease Control and Prevention (China CDC)	Chen; Dai; F.-H.; Hu, Y.; J.-H.; J.-J.; J.-L. and Zhu; Liu, Y.; Pei; Q.-M.; She; Song; T.-Y.; Tao; Tian; Wang; Wang, W.; Wu, F.; Xu, L.; Y.-L.; Y.-M.; Y.-Y.; Y.-Z.; Yu, B.; Z.-G.; Z.-W.; Zhang; Zhao, S.; Zheng
EPI_ISL_417186	National Institute for Communicable Diseases of the National Health Laboratory Service	National Institute for Communicable Diseases of the National Health Laboratory Service	A; Allam M; Bhiman JN; Ismail A; Khumalo Z; Kwenda S; Mohale T; Subramoney K; van Heusden P; von Gottberg
EPI_ISL_402119	National Institute for Viral Disease Control and Prevention, China CDC	National Institute for Viral Disease Control and Prevention, China CDC	Ji Wang; Weimin Zhou , Peihua Niu , Peipei Liu , Faxian Zhan , Weifeng Shi , Baoying Huang , Jun Liu , Li Zhao , Yao Meng , Xiaozhou He , Fei Ye , Na Zhu , Yang Li , Jing Chen , Wenbo Xu , George F. Gao , Guizhen Wu; Wenjie Tan , Xiang Zhao , Wenling Wang , Xuejun Ma , Yongzhong Jiang , Roujian Lu
EPI_ISL_527364	National Public Health Laboratory, National Centre for Infectious Diseases	National Public Health Laboratory, National Centre for Infectious Diseases	Cui L; Lin RTP; Mak TM; Octavia S; Zhou Z
EPI_ISL_837428	National Virus Reference Laboratory	National Virus Reference Laboratory	Cillian F De Gascun; Gabriel Gonzalez; Jonathan Dean; Michael Carr
EPI_ISL_906277	Nigeria Centre for Disease Control (NCDC)	African Centre of Excellence for Genomics of Infectious Diseases (ACEGID), Redeemer's University	Oluniyi P.E. et al
EPI_ISL_573371	Northumbria University / South Tees Hospitals NHS Foundation Trust / North Cumbria Integrated Care NHS Foundation Trust / North Tees and Hartlepool NHS Foundation Trust / Newcastle Hospitals NHS Foundation Trust	COVID-19 Genomics UK (COG-UK) Consortium	Andrew Nelson; Brendan Payne; Clive Graham; Darren L Smith; Debra Padgett; Edward Barton; Emma Swindells; Garren Scott; Gary Black; Gary Eltringham; Giles S Holt; Greg R Young; Jane Greenaway; Jennifer Collins; John Allan; Joshua Loh; Lynn Dover; Matthew Bashton; Mohammad A Tariq; Paul Baker; Sarah Essex; Steve Liggett; Wen C Yew; Yusri Taha
EPI_ISL_437535, EPI_ISL_667301	OHSU Lab Services Molecular Microbiology Lab	Oregon SARS-CoV-2 Genome Sequencing Center	Alec J. Hirsch; Andrew C. Adey; Benjamin N. Bimber; Brendan L. O'Connell; Brian J. O'Roak; Daniel N. Streblow; Donna Hansel; Guang Fan; Ruth V. Nichols; Sally Grindstaff; William B. Messer
EPI_ISL_889372	Olomouc University Hospital	Institute of Applied Biotechnologies a.s.	Kateřina Kvapilová; Martin Kašný; Ondřej Brzoň; Petr Klempt; Petr Kvapil
EPI_ISL_420564	Ospedale Civile Castel Di Sangro	Istituto Zooprofilattico Sperimentale dell'Abruzzo e Molise "G.Caporale"	Ancora M; Cammà C; Curini V; Di Domenico M; Di Pasquale A; Lorusso A; Mangone I; Marcacci M; Puglia I; Rinaldi A; Savini G
EPI_ISL_420563, EPI_ISL_429228, EPI_ISL_429232	Ospedale Civile Giuseppe Mazzini	Istituto Zooprofilattico Sperimentale dell'Abruzzo e Molise "G. Caporale"	Ancora M; Camma C; Cammà C; Curini V; Di Domenico M; Di Pasquale A; Lorusso A; Mangone I; Marcacci M; Puglia I; Rinaldi A; Savini G

EPI_ISL_420568, EPI_ISL_435145	Ospedale Civile Giuseppe Mazzini	Istituto Zooprofilattico Sperimentale dell'Abruzzo e Molise "G.Caporale"	Ancora M; Cammà C; Curini V; Di Domenico M; Di Pasquale A; Lorusso A; Mangone I; Marcacci M; Puglia I; Rinaldi A; Savini G
EPI_ISL_418257	Ospedale Civile Giuseppe Mazzini, Teramo	Istituto Zooprofilattico Sperimentale dell'Abruzzo e Molise "G.Caporale"	Ancora M; Cammà C; Curini V; Di Domenico M; Di Pasquale A; Lorusso A; Mangone I; Marcacci M; Puglia I; Rinaldi A; Savini G.
EPI_ISL_429236	Ospedale Civile S. Liberatore di Atri	Istituto Zooprofilattico Sperimentale dell'Abruzzo e Molise "G. Caporale"	Ancora M; Camma C; Curini V; Di Domenico M; Di Pasquale A; Lorusso A; Mangone I; Marcacci M; Puglia I; Rinaldi A; Savini G
EPI_ISL_436722, EPI_ISL_436724, EPI_ISL_436732	Ospedale Civile S. Liberatore di Atri	Istituto Zooprofilattico Sperimentale dell'Abruzzo e Molise "G.Caporale"	Ancora M; Cammà C; Curini V; Di Domenico M; Di Pasquale A; Lorusso A; Mangone I; Marcacci M; Puglia I; Rinaldi A; Savini G
EPI_ISL_420567	Ospedale Regionale San Salvatore	Istituto Zooprofilattico Sperimentale dell'Abruzzo e Molise "G.Caporale"	Ancora M; Cammà C; Curini V; Di Domenico M; Di Pasquale A; Lorusso A; Mangone I; Marcacci M; Puglia I; Rinaldi A; Savini G
EPI_ISL_435148, EPI_ISL_435150, EPI_ISL_435151	Ospedale SS Annunziata	Istituto Zooprofilattico Sperimentale dell'Abruzzo e Molise "G.Caporale"	Ancora M; Cammà C; Curini V; Di Domenico M; Di Pasquale A; Lorusso A; Mangone I; Marcacci M; Puglia I; Rinaldi A; Savini G
EPI_ISL_418256	Ospedale "San Liberatore" di Atri	Istituto Zooprofilattico Sperimentale dell'Abruzzo e Molise "G. Caporale"	Ancora M; Cammà C; Curini V; Di Domenico M; Di Pasquale A; Lorusso A; Mangone I; Marcacci M; Puglia I; Rinaldi A; Savini G.
EPI_ISL_492285	PHE South West Regional Laboratory, National Infection Service	Wellcome Sanger Institute for the COVID-19 Genomics UK (COG-UK) consortium	Barry Vipond; Cordelia Langford; David K. Jackson; Dominic Kwiatkowski; Dr Peter Muir; Ewan Harrison; Hannah Pymont; Ian Johnston; John Sillitoe on behalf of the Wellcome Sanger Institute COVID-19 Surveillance Team (http://www.sanger.ac.uk/covid-team); Rich Hopes; Roberto Amato; Sonia Goncalves; Stephanie Hutchings; and Alex Alderton
EPI_ISL_455030	Pathology West - NSW Health Pathology	NSW Health Pathology - Institute of Clinical Pathology and Medical Research; Westmead Hospital; University of Sydney	CIDM-PH et al.
EPI_ISL_418255	Presidio Ospedaliero "S. Spirito" - PESCARA	Istituto Zooprofilattico Sperimentale dell'Abruzzo e Molise "G. Caporale"	Cammà C; Di Pasquale A; Lorusso A; Mangone I; Marcacci M; Monaco F; Puglia I; Rinaldi A; Savini G
EPI_ISL_429226, EPI_ISL_429227	Presidio Ospedaliero Santo Spirito	Istituto Zooprofilattico Sperimentale dell'Abruzzo e Molise "G. Caporale"	Ancora M; Camma C; Curini V; Di Domenico M; Di Pasquale A; Lorusso A; Mangone I; Marcacci M; Puglia I; Rinaldi A; Savini G
EPI_ISL_418258	Presidio ospedaliero "Santo Spirito"	Istituto Zooprofilattico Sperimentale dell'Abruzzo e Molise "G. Caporale"	Ancora M; Cammà C; Curini V; Di Domenico M; Di Pasquale A; Lorusso A; Mangone I; Marcacci M; Puglia I; Rinaldi A; Savini G.
EPI_ISL_477204	Prof. Massimo Zollo CEINGE TASK-FORCE COVID19 - Regione Campania	Prof. Massimo Zollo CEINGE TASK-FORCE COVID19 - Regione Campania	; 2; 2* 1 CEINGE Biotecnologie Avanzate; 8* and Massimo Zollo1; Angelo Boccia2; Azienda Sanitaria Ospedali dei Colli; Claudia Tiberio4; Dae young Kong8; Fatemeh asadzadeh1; Giorgia Borriello3; Giovanna Fusco3; Giovanni Paolella1; Giuseppe Castaldo1; Hong Yeoul Kim 7; Indust; Italia 2 Dipartimento di Medicina Molecolare e Biotecnologie Mediche DMMBM University of Naples Federico II; Italia 3 Istituto Zooprofilattico Sperimentale del Mezzogiorno; Italia 4 -U.O.C. di Patologia Clinica Ospedale D. Cotugno; Italia 6 Department of Microbiology; Italy. 5 Università La Sapienza di Roma; Jae Ho Jung6; Jae Myun Lee5; Korea 8 Haim bio co.; Korea 7 Department of Surgery; Kyong Seop Yun7; Laura Marrone1; Lorenzo Chiariotti1; Ltd; Luigi Atripaldi4; Marika Comegna1; Martina Bianchi4; Maurizio Viscardi3; Naples; Rino Cerino3; Roberto Siciliano1; Seoul; Sergio Brandi3; Stefano Pascarella4; Veronica Ferrucci1; Yonsei University College of Medicine
EPI_ISL_513529	Programa de Oncovirologia, Instituto Nacional de Câncer	Programa de Oncovirologia, Instituto Nacional de Câncer	Andreia C. de Melo; Brunna M. Alves; Claudia Cicala; James Arthos; João P.B. Viola; Juliana D. Siqueira; Livia R. Goes; Marcelo A. Soares
EPI_ISL_448279	Quadram Institute Bioscience	COVID-19 Genomics UK (COG-UK) Consortium	Alexander J Trotter; Alison E. Mather; Alp Aydin; Ana P. Tedim; Anastasia Kolyva; Andrew Bell; Andrew J. Page; Claire Stuart; Dave J. Baker; Gemma L. Kay; John Wain; Justin O'Grady; Leonardo de Oliveira Martins; Lizzie Meadows; Maria Diaz; Mark Webber; Muhammed Yasir; Nabil-Fareed Alikhan; Ngozi Elumogo; Nicholas M. Thomson; Rachael Stanley; Rachel Gilroy; Reenesh Prakash; Samir Dervisevic; Samuel Bloomfield; Steven Rudder; Thanh Le-Viet
EPI_ISL_693282	Queensland Health Forensic and Scientific Services	Queensland Health Forensic and Scientific Services	Son Nguyen et al
EPI_ISL_571604	Quest Diagnostics	Quest Diagnostics	Anderson, B.; D.F.; Gerasimova, A.; Grover, D.; Hua, M.; K.E.; Kagan; Lacbawan, F.; Liu Y.; Livingston; Owen, R.; R.M.; Rosenthal; S.H.; Shalhout
EPI_ISL_413575	RIVM	Erasmus Medical Center	Anne van der Linden; Annemiek van der Eijk; Aura Timen; Bas Oude Munnink; Claudia Schapendonk; Corien Swaan; Corine GeurtsvanKessel; David Nieuwenhuijse; Irina Chestakova; Jeroen van Kampen; Jolanda Voermans; Madelief Mollers; Manon Haverkate; Marion Koopmans; Mark Pronk; Mart Stein; Pascal Lexmond; Reina Sikkema; Richard Molenkamp; Sandra Kengne Kamga Mobou; on behalf of the Dutch national COVID-19 response team.
EPI_ISL_436725	RSA/RP Villa San Giovanni - Gruppo Edos	Istituto Zooprofilattico Sperimentale dell'Abruzzo e Molise "G.Caporale"	Ancora M; Cammà C; Curini V; Di Domenico M; Di Pasquale A; Lorusso A; Mangone I; Marcacci M; Puglia I; Rinaldi A; Savini G
EPI_ISL_466532, EPI_ISL_485604	Respiratory Virus Unit, Microbiology Services Colindale, Public Health England	Respiratory Virus Unit, Microbiology Services Colindale, Public Health England	PHE Covid Sequencing Team
EPI_ISL_804348	Respiratory Virus Unit, National Infection Service, Public Health England	COVID-19 Genomics UK (COG-UK) Consortium	PHE Covid Sequencing Team
EPI_ISL_435149, EPI_ISL_435153, EPI_ISL_435154, EPI_ISL_435155, EPI_ISL_436726	SERVIZIO DI IGIENE E SANITÀ PUBBLICA ASL Teramo	Istituto Zooprofilattico Sperimentale dell'Abruzzo e Molise "G.Caporale"	Ancora M; Cammà C; Curini V; Di Domenico M; Di Pasquale A; Lorusso A; Mangone I; Marcacci M; Puglia I; Rinaldi A; Savini G
EPI_ISL_833106	SIESP DIPARTIMENTO DI PREVENZIONE TERAMO C.DA CASALENA	Istituto Zooprofilattico Sperimentale dell'Abruzzo e Molise "G. Caporale"	Ancora M; Calistri P; Cammà C; Curini V; Delli Compagni E; Di Domenico M; Di Pasquale A; Lorusso A; Mangone I; Marcacci M; Puglia I; Rinaldi A; Savini G
EPI_ISL_875566	SIESP L'AQUILA	Istituto Zooprofilattico Sperimentale dell'Abruzzo e Molise "G. Caporale"	Ancora M; Calistri P; Cammà C; Curini V; Di Domenico M; Di Pasquale A; Lorusso A; Mangone I; Marcacci M; Puglia I; Rinaldi A; Savini G; Scialabba S
EPI_ISL_660307	Servicio de Microbiología, Laboratori Clínic Metropolitana Nord. Hospital Universitari Germans Trias i Pujol. Institut d'Investigació en Ciències de la Salut Germans Trias i Pujol (IGTP)	SeqCOVID-SPAIN consortium/IBV(CSIC)	Adrián Antuori; Anabel Fernández; Anna Not; Antoni E. Bordoy; Elisa Martró; Nona Romaní and SeqCOVID-SPAIN consortium
EPI_ISL_796110	Servicio de Microbiología. Hospital General Universitario de Castellón	SeqCOVID-SPAIN consortium/IBV(CSIC)	María Dolores Tirado Balaguer and SeqCOVID-SPAIN consortium; Rosario Moreno Muñoz
EPI_ISL_1014733	Servicio de Microbiología. Hospital Ramón y Cajal. (CIBERESP)	SeqCOVID-SPAIN consortium/IBV(CSIC)	Jose Mª González-Alba; Juan C Galán and SeqCOVID-SPAIN consortium; L. Olavarrieta; Val Fernández
EPI_ISL_435152	Servizio di Igiene, Epidemiologia e Sanità Pubblica (SIESP) Avezzano	Istituto Zooprofilattico Sperimentale dell'Abruzzo e Molise "G.Caporale"	Ancora M; Cammà C; Curini V; Di Domenico M; Di Pasquale A; Lorusso A; Mangone I; Marcacci M; Puglia I; Rinaldi A; Savini G
EPI_ISL_436730	Servizio di igiene epidemiologia e sanità pubblica (Siesp) Chieti	Istituto Zooprofilattico Sperimentale dell'Abruzzo e Molise "G.Caporale"	Ancora M; Cammà C; Curini V; Di Domenico M; Di Pasquale A; Lorusso A; Mangone I; Marcacci M; Puglia I; Rinaldi A; Savini G
EPI_ISL_412912	State Health Office Baden-Württemberg	Charité Universitätsmedizin Berlin, Institute of Virology	Barbara Mühlemann; Christian Drosten; Julia Schneider; Jörn Beheim-Schwarzbach; Rainer Oehme; Silke Fischer; Talitha Veith; Terry Jones; Victor M Corman
EPI_ISL_491043	Suceava County Emergency Hospital	"Stefan cel Mare" University Metagenomics Lab	Antoniadis Panagiotis et al.; Lobiuc Andrei
EPI_ISL_529150	Technology Centre, Guangzhou Customs	Technology Centre, Guangzhou Customs	Dai, J.; Huang, J.; Huang, S.; Li, X.; Shi, Y.; Sun, F.; Sun, J.; Wang, Y.; Zhang, Z.; Zheng, K.; Zhu, .; Zhuang, Z.
EPI_ISL_577630	The National Institute of Public Health	State Veterinary Institute Prague	A; D; H; J; Jirincova; L; Nagy; Novakova; Trnka; Vecerova
EPI_ISL_685438	Tokyo Metropolitan Institute of Public Health	Pathogen Genomics Center, National Institute of Infectious Diseases	Kentaro Itokawa; Makoto Kuroda; Masanori Hashino; Rina Tanaka; Tsuyoshi Sekizuka
EPI_ISL_977235	ULSS 5 Polesana	Istituto Zooprofilattico Sperimentale delle Venezie	Adelaide Milani; Alessia Schivo; Alice Fusaro; Ambra Pastori; Annalisa Salviato; Antonia Ricci; Bianca Zecchin; Calogero Terregino; Erika Giorgia Quaranta; Isabella Monne
EPI_ISL_422437, EPI_ISL_422438, EPI_ISL_452181, EPI_ISL_452182, EPI_ISL_452183, EPI_ISL_452184, EPI_ISL_452185, EPI_ISL_452186, EPI_ISL_452187, EPI_ISL_452188, EPI_ISL_452189			
see above	ULSS9 Distretto di Bussolengo	Istituto Zooprofilattico Sperimentale delle Venezie	Adelaide Milani; Alessia Schivo; Alice Fusaro; Ambra Pastori; Annalisa Salviato; Antonia Ricci; Bianca Zecchin; Calogero Terregino; Erika Giorgia Quaranta; Isabella Monne
EPI_ISL_452190, EPI_ISL_452191	ULSS9 Distretto di San Bonifacio	Istituto Zooprofilattico Sperimentale delle Venezie	Adelaide Milani; Alessia Schivo; Alice Fusaro; Ambra Pastori; Annalisa Salviato; Antonia Ricci; Bianca Zecchin; Calogero Terregino; Erika Giorgia Quaranta; Isabella Monne
EPI_ISL_427238	UW Virology Lab	UW Virology Lab	Alexander Greninger; Hong Xie; Keith Jerome; Pavitra Roychoudhury
EPI_ISL_734790, EPI_ISL_735249	UZ Leuven, National Reference Laboratory for Coronaviruses, Laboratory Medicine, Leuven, Belgium	KU Leuven, Rega Institute, Clinical and Epidemiological Virology	Bert Vanmechelen; Joan Marti-Carerras; Piet Maes; Tony Wawina-Bokalanga
EPI_ISL_738021	Uganda Central Public Health Lab and Uganda Virus Research Institute	MRC/UVRI & LSHTM Uganda Research Unit	Dan Lule Bugembe; Matthew Cotten; My V.T. Phan; Pontiano Kaleebu et al.
EPI_ISL_776452	University Medical Center Hamburg Eppendorf	Heinrich Pette Institute, Leibniz Institute for Experimental Virology	Adam Grundhoff; Alexis Robitaille; Johannes Knobloch; Martin Aepfelbacher; Nicole Fischer; Thomas Günther
EPI_ISL_1168420	VA Connecticut Healthcare System	Grubaugh Lab - Yale School of Public Health	Chantal Vogels; Danielle Plank; Isabel Ott; Joseph Fauver; Mary Petrone; Nathan Grubaugh; Shaili Gupta; Tara Alpert
EPI_ISL_426783	Victorian Infectious Diseases Reference Laboratory (VIDRL)	Microbiological Diagnostic Unit Public Health Laboratory and Victorian Infectious Diseases Reference Laboratory, Doherty Institute	Caly L.; Druce J.; Sait, M.; Schultz M.; Seemann T.; Sherry, N.
EPI_ISL_480707	Victorian Infectious Diseases Reference Laboratory (VIDRL)	VIDRL and MDU-PHL	Caly L.; Druce J.; Sait, M.; Schultz M.; Seemann T.; Sherry, N.
EPI_ISL_435146, EPI_ISL_435147	Villa Serena del Dr. Leonardo Petruzzi	Istituto Zooprofilattico Sperimentale dell'Abruzzo e Molise "G.Caporale"	Ancora M; Cammà C; Curini V; Di Domenico M; Di Pasquale A; Lorusso A; Mangone I; Marcacci M; Puglia I; Rinaldi A; Savini G
EPI_ISL_451792, EPI_ISL_476114, EPI_ISL_483654, EPI_ISL_498623, EPI_ISL_500906, EPI_ISL_539376, EPI_ISL_603737, EPI_ISL_796577, EPI_ISL_1002504			
see above	Viollier AG	Department of Biosystems Science and Engineering, ETH Zürich	Andrea Patrignani; Andreia Cabral de Gouvea; Catharine Aquino; Chaoran Chen; Christian Beisel; Christiane Beckmann; Christoph Noppen; David Dreifuss; Doris Popovic; Elodie Burcklen; Griffin White; Ina Nissen; Ivan Topolsky; Jay Tracy; Katharina Jahn; Lara Fuhrmann; Laura Neff; Lennart Opitz; Maria Domenica Moccia; Maurice Redondo; Natascha Santacroce; Niko Beerenwinkel; Noemie Santamaria de Souza; Olivier Kobel; Pedro Ferreira; Philipp Jablonski; Ralph Schlapbach; Sarah Nadeau; Simon Grüter; Sophie Seidel; Susana Posada-Céspedes; Tanja Stadler; Timothy Sykes; Tobias Schär
EPI_ISL_609976	Virginia DCLS	Virginia DCLS	Virginia DCLS
EPI_ISL_433123	Virology Department, Royal Infirmary of Edinburgh, NHS Lothian / School of Biological Sciences, University of Edinburgh / Institute of Genetics and Molecular Medicine, University of Edinburgh	COVID-19 Genomics UK (COG-UK) Consortium	Balcaza C; Colquhoun R; Dewar R; Gallagher M; Hill V; Jackson B; McCrone JT; McHugh M; O'Toole A; Rambaut A; Rooke S; Templeton K; Williams TC; Yu X

EPI_ISL_417491	Virology Laboratory, Department of Biomedical Sciences and Public Health, University Politecnica delle Marche	Virology and Legal Medicine Laboratories, Department of Biomedical Sciences and Public Health, University Politecnica delle Marche	Alessandrini, F.; Bagnarelli, P.; Caucci, S.; Di Sante, L.; Menzo, S.; Onofri, V.; Tagliabracci, A.; Turchi, C.
EPI_ISL_507266	WHO National Influenza Centre Russian Federation	WHO National Influenza Centre Russian Federation	Andrey Komissarov; Anna Ivanova; Artem Fadeev; Daria Danilenko; Mariia Sergeeva
EPI_ISL_525588	Wadsworth Center, New York State Department of Health	Wadsworth Center, New York State Department of Health	Daryl M. Lamson; Erica Lasek-Nesselquist; Jonathan Plitnick; Kirsten St. George; Matthew D. Shudt; Navjot Singh; Sara Griesemer
EPI_ISL_474755	Wales Specialist Virology Centre Sequencing lab: Pathogen Genomics Unit	COVID-19 Genomics UK (COG-UK) Consortium	Alec Birchley; Alexander Adams; Amy Gaskin; Angela Marchbank; Bree Gatica-Wilcox; Catherine Moore; Jason Coombes; Joanne Watkins; Joel Southgate; Johnathan Evans; Laura Gifford; Lauren Gilbert; Lee Graham; Malorie Perry; Matthew Bull; Nicole Pacchiarini; Sally Corden; Sara Kumziene-Summerhayes; Sara Rey; Sarah Taylor; Simon Cottrell; Sophie Jones; Tom Connor
EPI_ISL_451345	West China Hospital of Sichuan University	State Key Laboratory of Biotherapy of Sichuan University	Baowen Du; Binwu Ying; Chao Tang; Chuan Chen; Hancheng Wei; Jia Geng; Jing-wen Lin; Lu Chen; Mingxia Yu; Minjin Wang; Weimin Li; Yongzhao Zhou
EPI_ISL_402124	Wuhan Jinyintan Hospital	Wuhan Institute of Virology, Chinese Academy of Sciences	Ding-Yu Zhang; Hao-Rui Si; Lei Zhang; Peng Zhou; Xing-Lou Yang; Yan Zhu; Zhengli Shi
EPI_ISL_861509	ZOTZ KLIMAS MVZ Düsseldorf-Centrum GbR ÜBAG für Labormedizin, Genetik, Zytologie, Pathologie	Center of Medical Microbiology, Virology, and Hospital Hygiene, University of Duesseldorf	Alexander Dilthey; Andreas Walker; Ashley-Jane Duplessis; Daniel Strelow; Jessica Nicolai; Jörg Timm; Katrin Hoffmann; Klaus Pfeffer; Lisanna Hülse; Malte Kohns Vasconcelos; Marek Korencak; Maximilian Damagnez; Nadine Lübke; Patrick Finzer; Rainer Zotz; Tobias Wienemann; Torsten Houwaart
