# Supplementary material for: On the Origin and Propagation of the COVID-19 Outbreak in the Italian Province of Trento, a Tourist Region of Northern Italy
Source: Viruses. 2022 Mar 11;14(3):580. doi: 10.3390/v14030580 (PMC8951735; doi:10.3390/v14030580)
Supplement: Supplementary file 1 [file viruses-14-00580-s001.zip › Supplementary_materials.pdf]

Supplementary materials

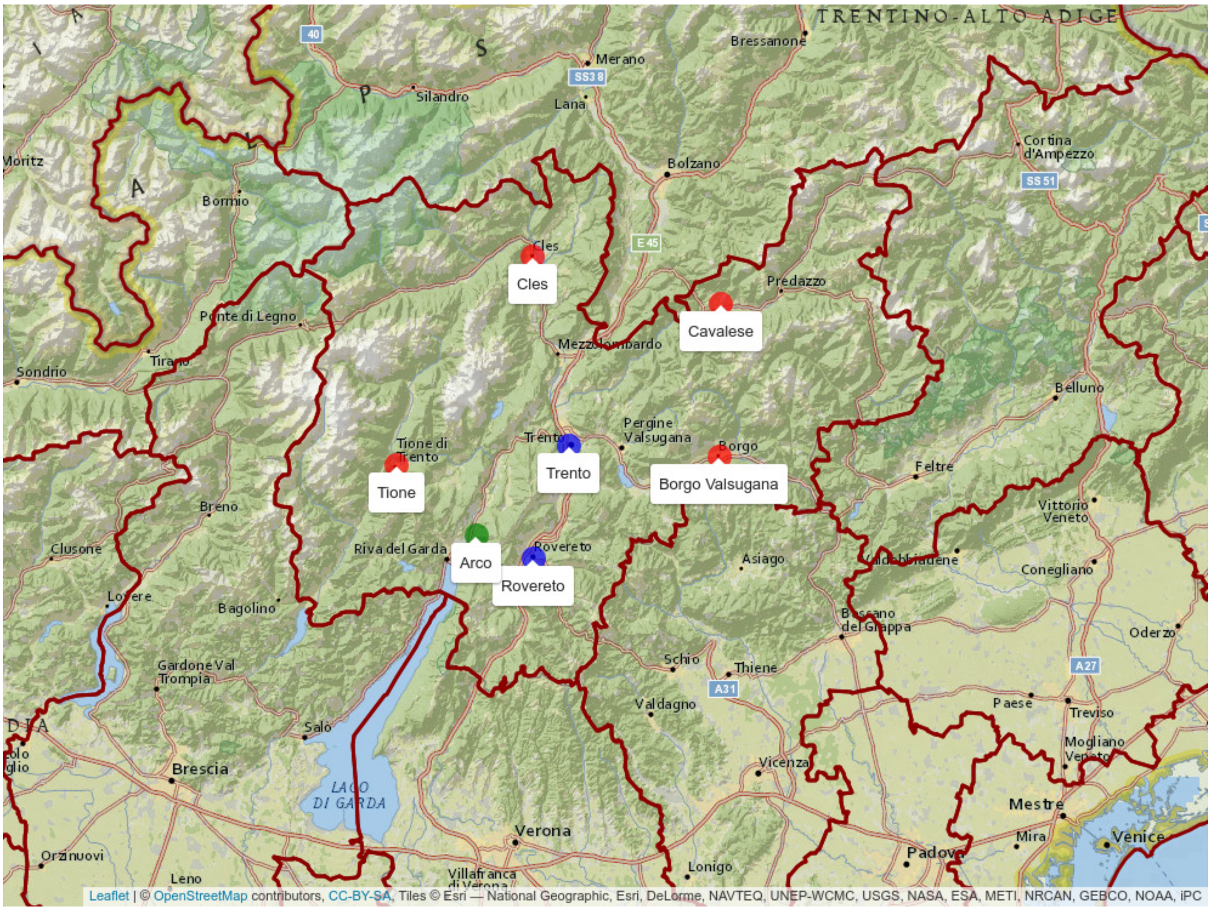

Supplementary Figure S1. Geographic location of the seven ERs present in Trentino.

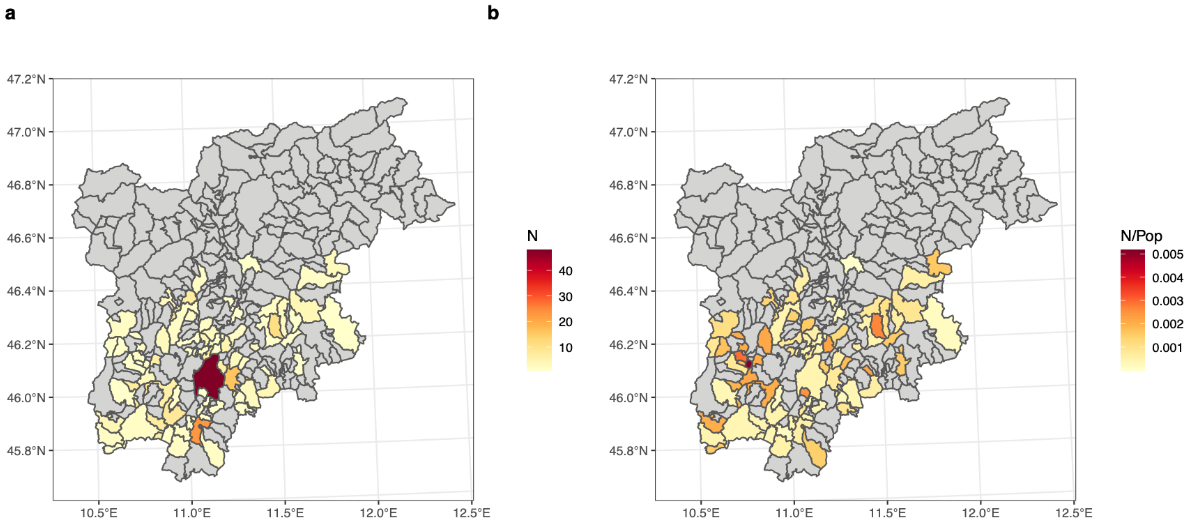

Supplementary Figure S2 a) Number of sequences analyzed for each municipality. b) same as a), normalized by the number of inhabitants.

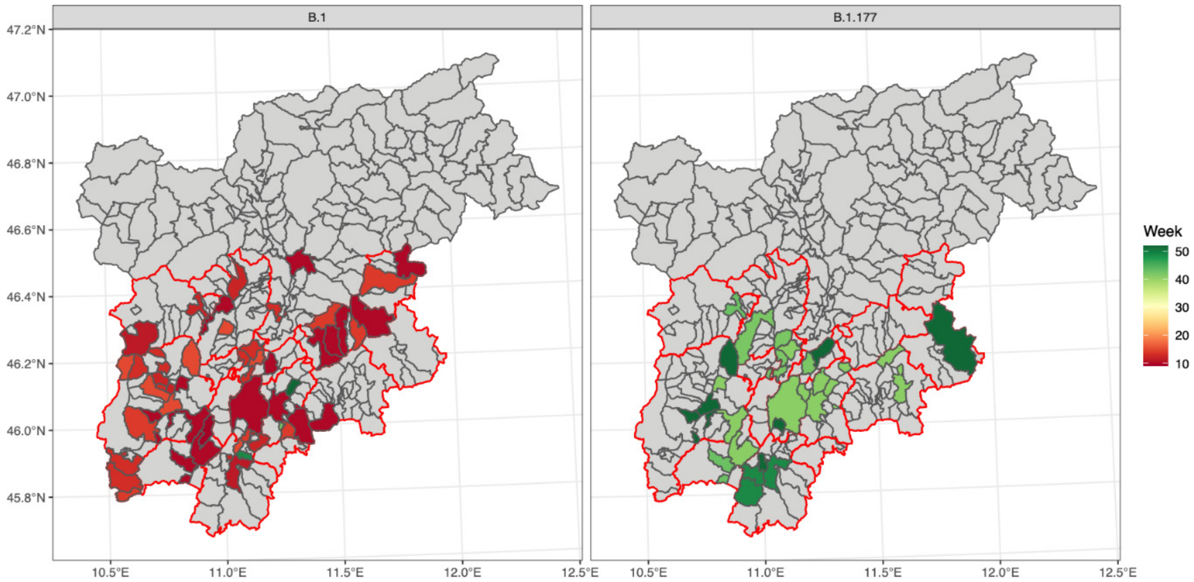

Supplementary Figure S3. First isolation of sequences from Pangolin clade B.1. and B.1.177. For each geographical area, we show the week in which the clade was first identified. While in most locations lineage B.1 appeared early in the first epidemic wave in many geographically unrelated areas, lineage B.1.177 spread in the second wave starting from the areas in the neighborhood of Trento and diffusing to more peripheral areas.

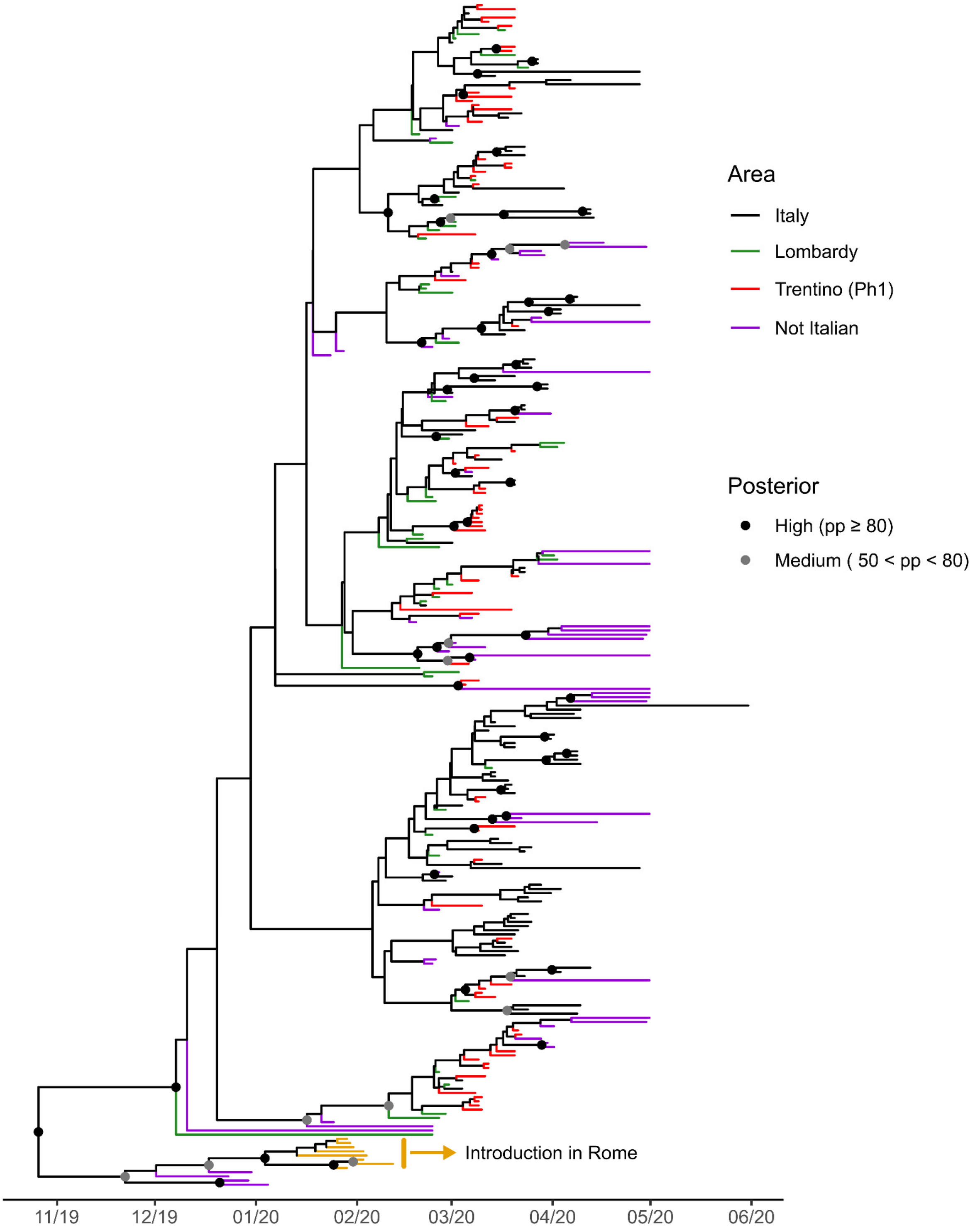

Supplementary Figure S4. Phylogenomic reconstruction of the early spread of SARS-CoV-2 in Trentino. Red branches identify the first batch of sequences from Trentino from samples collected in the first epidemic wave (Ph1; spring 2020); green branches and black branches identify Italian sequences, respectively from Lombardy or from other regions of Italy, while violet branches are used to represent sequences which are not of Italian origin. A well distinct and secondary introduction of the virus in the area of Rome is highlighted in yellow. All samples which are not coming from the Trentino batch (n=72) were downloaded from GISAID (n=212). Black dots mark nodes with high posterior probability (pp ≥ 80%), while gray dots mark nodes with intermediate

posterior probability (50% < pp < 80%). A timescale at the bottom of the figure indicates the month and year of the sample.

Tables

Supplementary Table S1: Ancillary information of the analysed samples.

Supplementary Table S2: Number of reads identified, through sequence alignment to the reference genome, for each amplicon in the 253 sequenced samples.

Supplementary Table S3: For each sample, the number of reads is reported alongside the number of unknown bases in the obtained consensus sequence (N), variants identified compared to the Whuan's reference genome (MN908947.3) and the Ct value.

Supplementary Table S4. Number of sequences for each Pangolin lineage in each of the two sampling periods (Lineage summary sheet). Pangolin classification and mutations found for the newly sequenced samples (Pangolin classification sheet).

Supplementary Table S5. Acknowledgement of Laboratories that contributed by submitting the sequences to GISAID for public use.
